# Supplementary material for: Geometric morphometric analysis of spore shapes improves identification of fungi
Source: PLoS One. 2021 Aug 5;16(8):e0250477. doi: 10.1371/journal.pone.0250477 (PMC8341628; doi:10.1371/journal.pone.0250477)
Supplement: S1 Appendix — (ZIP) [file pone.0250477.s002.zip › 3_latest/1_code/sizeVsShape_20210217.nb.html]

Spore shape vs. size


Code 

- Show All Code
- Hide All Code
- Download Rmd

# Spore shape vs. size

This is an R Notebook containing code and results of comparing the performance of shape and size traits for discrimination of species in *Subulicystidium*.

**New in this version**:  
\* this is largely a duplicate of the version 20210129 but now based on the (for sureness) re-run PCA from 17.02.2021  
\* removed the non-relevant here description of how to create a single NEF file. \* make illustrations and tables writing to the files with desired dimensions, into kind of ‘out’ folder

**To do in the next version**:  
\* implement report() in the end of the script \* move PCA analysis of NEFD to R  
\* I may also adjust longisporum name in the next analysis version when the data from Christina will be coming. Then my longisporum will become longisporum\_7

**Most important changes in the past**: \* analyses on image-level data, N of observations = 401. Each observation is a data from a single image, with information on min 1 spore and max 4 spores  
\* custom function for discriminant analysis that allows to shorten the script

## Setting the work

### All is pre-set correctly now

All code, data and results are organized into RStudio R project. After clicking the .Rproj file, this Notebook will be opened and working directory will be understood by R correctly as a directory which is parent for the code, data and results directories.

additionally, loading package “here” should notify on the correct working directory. “here” will be also very helpful for accessing files in the child directories along the script.


```
library(here)
```


```
here() starts at C:/Users/aordy/OneDrive/Documents/Projects/ARTS/2_studies/1_size_vs_shape/3_latest
```


```
library(conflicted)
conflict_prefer("here", "here")
```


```
[conflicted] Will prefer here::here over any other package
```


```
conflicted::conflict_prefer("rename", "dplyr")
```


```
[conflicted] Will prefer dplyr::rename over any other package
```

### This is how it was earlier

At the very start, assign in R studio as a working directory the directory which is parent for the code, data and results directories. Then *set\_here* function of the “here” package will pick this assignment by creating the file .here. Then all subsequent R sessions on this project will start with the right directory. To see more examples of “here” visit this link

## Load and proceed spore shape data

### Summarize symmetric variation

Load and process the table with PCA scores of the spores (symmetric variation)


```
scores_symm_raw <- read.csv(here("2_3_data_shape", "pca_202102", "1_symm", "30specimens_clean_symm.pcs"),  sep="\t")

# add columns to be filled in later with relevant IDs
scores_symm_raw <- cbind(Specimen_ID = NA, scores_symm_raw)
scores_symm_raw <- cbind(Image_ID = NA, scores_symm_raw)

# fill in column Specimen_ID based on the content of the column DATA_NAME
library(stringr)
strings <- c("^ARAN", "^CWU", "^KHL", "^LR", "^LY", "^Ordynets")
scores_symm_raw$Specimen_ID <- ifelse(str_detect(scores_symm_raw$DATA_NAME, paste(strings, collapse = "|")), gsub("^([^_]*_[^_]*)_.*$", "\\1", scores_symm_raw$DATA_NAME), gsub("^([^_]*_[^_]*_[^_]*)_.*$", "\\1", scores_symm_raw$DATA_NAME))

# fill in column Image_ID based on the content of the column DATA_NAME
scores_symm_raw$Image_ID <- ifelse(str_detect(scores_symm_raw$DATA_NAME, paste(strings, collapse = "|")), gsub("^([^_]*_[^_]*_[^_]*_[^_]*)_.*$", "\\1", scores_symm_raw$DATA_NAME), gsub("^([^_]*_[^_]*_[^_]*_[^_]*_[^_]*)_.*$", "\\1", scores_symm_raw$DATA_NAME))
```


Plot PCA scores as scatterplots (symmetric variation)


```
library(ggplot2)
pca_symm_plot<-ggplot(scores_symm_raw, aes(x=Specimen_ID, y=PC1, color=Specimen_ID)) +
geom_point()+
theme(axis.text.x = element_text(angle = 90))
plot(pca_symm_plot)
```


Summarize symmetric variation at the image ID level `.groups = 'drop'` argument in `summarise()` will help to remove a “friendly warning”  
`` `summarise()` ungrouping output (override with `.groups` argument) ``  
The explanation is available here


```
library(dplyr)
scores_symm_summ <- scores_symm_raw %>% 
  group_by(Image_ID) %>% 
  summarise(across(Specimen_ID, dplyr::first),
            across(PC1, mean),
            .groups = 'drop')
scores_symm_summ <- dplyr::rename(scores_symm_summ, PC1_symm_mean = PC1)
```

### Summarize asymmetric variation

Load and process the table with PCA scores for spores (asymmetric variation)


```
scores_asym_raw <- read.csv(here("2_3_data_shape", "pca_202102", "2_asym", "30specimens_clean_asym.pcs"),  sep="\t")

# add columns to be filled in later with relevant IDs
scores_asym_raw <- cbind(Specimen_ID = NA, scores_asym_raw)
scores_asym_raw <- cbind(Image_ID = NA, scores_asym_raw)

# fill in column Specimen_ID based on the content of the column DATA_NAME.
# "strings" object was created above and is valid here as is.
# Package "stringr" is necessary and was loaded earlier.
scores_asym_raw$Specimen_ID <- ifelse(str_detect(scores_asym_raw$DATA_NAME, paste(strings, collapse = "|")), gsub("^([^_]*_[^_]*)_.*$", "\\1", scores_asym_raw$DATA_NAME), gsub("^([^_]*_[^_]*_[^_]*)_.*$", "\\1", scores_asym_raw$DATA_NAME))

# fill in column Image_ID based on the content of the column DATA_NAME.
scores_asym_raw$Image_ID <- ifelse(str_detect(scores_asym_raw$DATA_NAME, paste(strings, collapse = "|")), gsub("^([^_]*_[^_]*_[^_]*_[^_]*)_.*$", "\\1", scores_asym_raw$DATA_NAME), gsub("^([^_]*_[^_]*_[^_]*_[^_]*_[^_]*)_.*$", "\\1", scores_asym_raw$DATA_NAME))
```


Plot PCA scores as scatterplots (asymmetric variation): PC1 vs PC2


```
library(ggplot2)
pca_asym_plot<-ggplot(scores_asym_raw, aes(x=PC1, y=PC2, color=Specimen_ID)) +
geom_point()
plot(pca_asym_plot)
```


Plotting PCA scores as scatterplots (asymmetric variation): PC1 vs PC3


```
library(ggplot2)
pca_asym_plot<-ggplot(scores_asym_raw, aes(x=PC1, y=PC3, color=Specimen_ID)) +
geom_point()
plot(pca_asym_plot)
```


Summarize asymmetric variation at image level


```
library(dplyr)
scores_asym_summ <- scores_asym_raw %>% 
  group_by(Image_ID) %>% 
  summarise(PC1_asym_mean=mean(PC1),
            PC2_asym_mean=mean(PC2),
            PC3_asym_mean=mean(PC3), 
            .groups = 'drop')
```

### Summarize global variation

Load and process the table with PCA scores for spores (global variation)


```
scores_glob_raw <- read.csv(here("2_3_data_shape", "pca_202102", "3_glob", "30specimens_clean_glob.pcs"),  sep="\t")

# add columns to be filled in later with relevant IDs
scores_glob_raw <- cbind(Specimen_ID = NA, scores_glob_raw)
scores_glob_raw <- cbind(Image_ID = NA, scores_glob_raw)

# fill in column Specimen_ID based on content of the column DATA_NAME
# "strings" object was created above and is valid here as is
# Package "stringr" is necessary and was loaded earlier
scores_glob_raw$Specimen_ID <- ifelse(str_detect(scores_glob_raw$DATA_NAME, paste(strings, collapse = "|")), gsub("^([^_]*_[^_]*)_.*$", "\\1", scores_glob_raw$DATA_NAME), gsub("^([^_]*_[^_]*_[^_]*)_.*$", "\\1", scores_glob_raw$DATA_NAME))

scores_glob_raw$Image_ID <- ifelse(str_detect(scores_glob_raw$DATA_NAME, paste(strings, collapse = "|")), gsub("^([^_]*_[^_]*_[^_]*_[^_]*)_.*$", "\\1", scores_glob_raw$DATA_NAME), gsub("^([^_]*_[^_]*_[^_]*_[^_]*_[^_]*)_.*$", "\\1", scores_glob_raw$DATA_NAME))
```


Plotting PCA scores as scatterplots (global variation): PC1 vs PC2


```
library(ggplot2)
pca_glob_plot<-ggplot(scores_glob_raw, aes(x=PC1, y=PC2, color=Specimen_ID)) +
geom_point()
plot(pca_glob_plot)
```


Summarize global variation at the image level


```
library(dplyr)
scores_glob_summ <- scores_glob_raw %>% 
  group_by(Image_ID) %>% 
  summarise(PC1_glob_mean=mean(PC1),
            PC2_glob_mean=mean(PC2), 
            .groups = 'drop')
```

## Load and proceed spore size data

Spore size data from separate specimens get pooled.  
I will suppress the warning message “Missing column names filled in: ‘X1’ [1]” that is generated by “read.csv” function which is forced to give a name to the unnamed column.


```
library(here)
library(readr)
import.size <- dir(here("2_2_data_size", "v20201215"), pattern = "*.csv", full.names = T)
data_size_raw <- suppressWarnings(plyr::ldply(import.size, read_csv))
```


Then the spreadsheet is transformed to put length and width of each spore into separate columns of one row.  
Warning message from dplyr when binding length and width columns “New names: \* X1 -> X1…1…” that appears after the code chunk can be ignored.  
Then, length to width ratio is added as an additional column.  
Finally, column “Image\_ID” is created.


```
library(dplyr)
# find spore lengths
data_size_raw_odd <- data_size_raw %>% dplyr::slice(which(row_number() %% 2 == 1))
colnames(data_size_raw_odd)[which(names(data_size_raw_odd) == "Label")] <- "Photo_ID"

# find spore widths
data_size_raw_even <- data_size_raw %>% dplyr::slice(which(row_number() %% 2 == 0)) 
colnames(data_size_raw_even)[which(names(data_size_raw_even) == "Length")] <- "Width"

# bind length and width values and leave just necessary values
data_size_tiny <- bind_cols(data_size_raw_odd, data_size_raw_even)
```


```
New names:
* X1 -> X1...1
* Min -> Min...3
* Max -> Max...4
* Angle -> Angle...5
* X1 -> X1...7
* ...
```


```
data_size_tiny_simple <-data_size_tiny[, c("Photo_ID", "Length", "Width")]

# add length to width ratio as an additional trait
data_size_tiny_simple <- transform(data_size_tiny_simple, Length_to_width_ratio = Length / Width)
data_size_tiny_simple$Length_to_width_ratio <- round(data_size_tiny_simple$Length_to_width_ratio, 2)

# add column for image IDs
data_size_tiny_simple <- cbind(Image_ID = NA, data_size_tiny_simple)

# fill the column for image IDs
## code that uses regex is based on the solution for gsub 
## ## https://stackoverflow.com/questions/39366759/regex-to-extract-values-between-2-underscores-including-a-value-that-is-an-unde
## https://stackoverflow.com/questions/7449564/regex-return-all-before-the-second-occurrence 

library(stringr)
strings <- c("^ARAN", "^CWU", "^KHL", "^LR", "^LY", "^Ordynets")
data_size_tiny_simple$Image_ID <- ifelse(str_detect(data_size_tiny_simple$Photo_ID, paste(strings, collapse = "|")), gsub("^([^_]*_[^_]*_[^_]*_[^_]*)_.*$", "\\1", scores_symm_raw$DATA_NAME), gsub("^([^_]*_[^_]*_[^_]*_[^_]*_[^_]*)_.*$", "\\1", data_size_tiny_simple$Photo_ID))

#remove ".bmp" string pattern from some image IDs 
data_size_tiny_simple$Image_ID <- gsub(pattern = ".bmp*", replacement = "", x = data_size_tiny_simple$Image_ID)
```


Summarize size data at the image level


```
library(dplyr) 
size_summ <- data_size_tiny_simple %>% 
  group_by(Image_ID) %>% 
  summarise(Length_mean=mean(Length),
            Width_mean=mean(Width),
            Length_to_width_ratio_mean=mean(Length_to_width_ratio),
            N_spores = n(),
              .groups = 'drop')
```


The chunk below allows to add a variable with an alternative size definition, following Claude 2008 book (p. 98-99).  
It is a possible alternative to the single length and width measurements.  
This measure of size would correlate less with shape variables, which is a desired behavior.  
However, if used in in discriminant analysis, it did not allow to gain in species prediction (tried in Feb 2020, not shown in the code below but can be easily added).


```
## needs dplyr
# size_summ$Size_scaled <- NA
# size_summ$Size_scaled <- sqrt(size_summ$Length_mean * size_summ$Width_mean)
# size_summ <- size_summ %>% relocate(Size_scaled, .before = 'Length_mean') # cosmetic changes
```

## Unite shape and size data

### Check that Images IDs are in the same order in all objects. They will serve as grouping variable

Check for image level data (n=401 in Dez 2020). Here, no need to sort the data in each group because they are all in the sam order after applying `summarise()` function


```
sapply(list(scores_symm_summ$Image_ID, scores_asym_summ$Image_ID, scores_glob_summ$Image_ID),
            FUN = all.equal.list, size_summ$Image_ID)
```


```
[1] TRUE TRUE TRUE
```

### Create a united table with all traits

Join separate dataframes


```
library(dplyr)
traits_merged <- scores_symm_summ %>% right_join(scores_asym_summ, by = "Image_ID") %>% right_join(scores_glob_summ, by = "Image_ID") %>% right_join(size_summ, by = "Image_ID")
```


In this way (chunk below) I exported specimen IDs (n = 30) and assigned them manualy to species for further analysis. If the new specimens will be added to the dataset after summer 2020, the table with species name labels should be updated.


```
# not run
readr::write_csv(as.data.frame(table(traits_merged$Specimen_ID)), file = here("3_results", "species_names_raw.csv"))
```


Add a column with species names based on data from another table


```
traits_merged <- cbind(Species_ID = NA, traits_merged)
traits_merged$Species_ID <- traits_merged$Specimen_ID
sp_names <- read.csv(here("3_results", "species_names.csv"),  sep=",", stringsAsFactors=FALSE)

library(data.table)
```


```
Registered S3 method overwritten by 'data.table':
  method           from
  print.data.table     
data.table 1.13.4 using 4 threads (see ?getDTthreads).  Latest news: r-datatable.com
```


```
traits_merged_dt <- data.table(traits_merged)
traits_merged_dt[, Species_ID := as.character(factor(Species_ID, labels = sp_names$Species_ID))]
traits_merged <- data.frame(traits_merged_dt)
```


Export the table with traits on image level for supplementary


```
readr::write_csv(traits_merged, file = here("3_results", "traits_per_image.csv"))
```

## Data exploration

### Scatterplots for separate traits


```
library(ggplot2)
# Visualize asymmetric variation at specimen level: PC1 vs PC2
gg_asym_PC1vPC2 <- ggplot(traits_merged, aes(x=PC1_asym_mean, y=PC2_asym_mean, color=Species_ID)) +
  geom_point(size = 1, alpha = 0.7) +
  labs(tag = "A", x = "PC1 asymmetric", y = "PC2 asymmetric", color ="Species")

# Visualize asymmetric variation at specimen level: PC1 vs PC3
gg_asym_PC1vPC3 <- ggplot(traits_merged, aes(x=PC1_asym_mean, y=PC3_asym_mean, color=Species_ID)) +
  geom_point(size = 1, alpha = 0.7) + 
  labs(tag = "B", x = "PC1 asymmetric", y = "PC3 asymmetric")

# Visualize symmetric shape variation at specimen level: PC1
# Note that the values on axis y (actually x before flipping) are in reverse order for comparability with the values for Q 
gg_symm_PC1 <- ggplot(traits_merged, aes(x=PC1_symm_mean, color=Species_ID)) +
geom_boxplot() +
coord_flip() +
scale_x_reverse() + 
labs(tag = "C", x = "PC1 symmetric")

# Visualize global variation at specimen level: PC1 vs PC2
gg_glob_PC1vPC2 <-ggplot(traits_merged, aes(x=PC1_glob_mean, y=PC2_glob_mean, color=Species_ID)) + 
  geom_point(size = 1, alpha = 0.7) +
  labs(tag = "D", x = "PC1 global", y = "PC2 global")

# Visualize size variation at specimen level: Length vs width
gg_size_LvW <-ggplot(traits_merged, aes(x=Length_mean, y=Width_mean, color=Species_ID)) +
  geom_point(size = 1, alpha = 0.7) +
  labs(tag = "E", x = "Length", y = "Width")

# Visualize Q variation at specimen level
gg_size_Q <-ggplot(traits_merged, aes(x=Length_to_width_ratio_mean, color=Species_ID)) +
  geom_boxplot() + 
  coord_flip() + 
  labs(tag = "F", x = "Length to width ratio")
```

### Scatterplots for traits in a single combined plot


```
#conflicted::conflict_prefer("rename", "dplyr")
library(ggpubr)
gg_pcas <- ggarrange(gg_asym_PC1vPC2, gg_asym_PC1vPC3, gg_symm_PC1, gg_glob_PC1vPC2, gg_size_LvW, gg_size_Q, common.legend = TRUE, legend = "bottom", align = "hv") + 
  theme(plot.margin = margin(0.3, 0.6, 0.3, 0.3, 'cm'))
```


Print the plots to a file(s)


```
# tiff
tiff(file = here::here("3_results", "fig_pca.tiff"), height= 13, width=19, units = 'cm', res = 600, 
     compression = "lzw", family = "sans")
print(gg_pcas)
dev.off()
```


```
null device 
          1
```


```
# # pdf
# pdf(file = here::here("3_results", "fig_pca.pdf"), height=5.0, width=7.5)
# print(gg_pcas)
# dev.off()
# 
# # png
# png(file = here::here("3_results", "fig_pca.png"), height= 13, width=19, units = 'cm', res = 500)
# print(gg_pcas)
# dev.off()
```

### Trait data: normality test for each level of the grouping variable


```
shL <- apply(traits_merged[,4:12], 2,  function(x) {RVAideMemoire::byf.shapiro(x ~ Species_ID, data = traits_merged)$tab}) # creates list
shD <- data.table::rbindlist(shL, idcol = T) # converts series of lists to dataframe
# lines just below require dplyr
shD$Species_ID <- rep(levels(factor(traits_merged$Species_ID)), 9) # re-create columnsof species IDs
shD %>% relocate(Species_ID, .after = 1) %>% rename(Trait = .id, Shapiro_W = W, Shapiro_p = 'p-value') -> shD # cosmetic changes 
shD$Shapiro_p <- as.numeric(format(shD$Shapiro_p, scientific=FALSE)) # show p values as decimals
shD
```


In how many % of cases (traits by species) the normality was not met?


```
shapiroNN <- shD$Shapiro_p <= 0.05
nrow(shD[shapiroNN,]) / nrow(shD)
```


```
[1] 0.2444444
```


There is no universal multivariate normality for levels (defined by species) within traits.

### Trait data: test of variance equality in trait variables between species


```
heplots::leveneTests(traits_merged[,4:12], factor(traits_merged$Species_ID))
```


```
Levene's Tests for Homogeneity of Variance (center = median)

                           df1 df2 F value    Pr(>F)    
PC1_symm_mean                9 391  7.3153 7.248e-10 ***
PC1_asym_mean                9 391 10.5964 9.896e-15 ***
PC2_asym_mean                9 391  6.5503 1.024e-08 ***
PC3_asym_mean                9 391  6.4990 1.224e-08 ***
PC1_glob_mean                9 391  7.6874 2.005e-10 ***
PC2_glob_mean                9 391  9.5768 3.099e-13 ***
Length_mean                  9 391 13.9178 < 2.2e-16 ***
Width_mean                   9 391 18.6954 < 2.2e-16 ***
Length_to_width_ratio_mean   9 391 18.9704 < 2.2e-16 ***
---
Signif. codes:  0 ‘***’ 0.001 ‘**’ 0.01 ‘*’ 0.05 ‘.’ 0.1 ‘ ’ 1
```


For each of the traits, covariance between the groups defined by species are not equal.

### Trait data: overall distributions


```
par(mfrow = c(3,3))
hist(traits_merged$PC1_symm_mean, main = NULL, xlab = "PC1 symmetric")
hist(traits_merged$PC1_asym_mean, main = NULL, xlab = "PC1 asymmetric", ylab = NULL)
```


```
hist(traits_merged$PC2_asym_mean, main = NULL, xlab = "PC2 asymmetric", ylab = NULL)

hist(traits_merged$PC3_asym_mean, main = NULL, xlab = "PC3 asymmetric")
```


```
hist(traits_merged$PC1_glob_mean, main = NULL, xlab = "PC1 global", ylab = NULL) 
hist(traits_merged$PC2_glob_mean, main = NULL, xlab = "PC2 global", ylab = NULL)
```


```
hist(traits_merged$Length_mean, main = NULL, xlab = "Length")
hist(traits_merged$Width_mean, main = NULL, xlab = "Width", ylab = NULL)
```


```
hist(traits_merged$Length_to_width_ratio_mean, main = NULL, xlab = "Length to width ratio", ylab = NULL)
```


```
# if work from the console, reset graphic settings back with dev.off()
```

### Multicollinearity check: overall correlations between variables

#### Way 1

Multicollinearity check: corrplot  
Get correlation values (Spearman coefficient)


```
traits_merged_forCorr <- dplyr::select(traits_merged,-c("Species_ID", "Image_ID", "Specimen_ID", "N_spores"))
#names(traits_merged_forCorr) this is to get the actual names and to costumize them manually as I do below
names(traits_merged_forCorr) <- c("PC1 symmetric", "PC1 asymmetric", "PC2 asymmetric", 
                                  "PC3 asymmetric", "PC1 global", "PC2 global",
                                  "Length", "Width", "Length to width ratio")
M <- cor(traits_merged_forCorr, method="spearman")
```


Computing the p-value of correlations  
To compute the matrix of p-value, a custom R function is used. Source is here


```
# mat : is a matrix of data
# ... : further arguments to pass to the native R cor.test function
cor.mtest <- function(mat, ...) {
    mat <- as.matrix(mat)
    n <- ncol(mat)
    p.mat<- matrix(NA, n, n)
    diag(p.mat) <- 0
    for (i in 1:(n - 1)) {
        for (j in (i + 1):n) {
            tmp <- cor.test(mat[, i], mat[, j], ...)
            p.mat[i, j] <- p.mat[j, i] <- tmp$p.value
        }
    }
  colnames(p.mat) <- rownames(p.mat) <- colnames(mat)
  p.mat
}
```


Get p (significance) values for Spearman correlation coefficient These could not be estimated for all cross-comparisons, apparently due to small data size.


```
p.mat <- cor.mtest(M, method="spearman", exact=FALSE)
```


Plot correlation values with highlighting only significant (at p=0.05) correlation values


```
library(corrplot)
```


```
corrplot 0.84 loaded
```


```
col <- colorRampPalette(c("#4477AA", "#77AADD", "#FFFFFF", "#EE9988", "#BB4444"))


tiff(file = here::here("3_results", "fig_corr.tiff"), height = 16.5, width = 16.5, units = 'cm', res = 600,
     compression = "lzw", family = "sans")
corrplot(M, method="color", col=col(200),  
         type="upper", 
         addCoef.col = "black", # Add coefficient of correlation
         tl.col="black", tl.srt=45, #Text label color and rotation
         # Combine with significance
         p.mat = p.mat, sig.level = 0.05, insig = "blank", 
         # hide correlation coefficient on the principal diagonal
         diag=FALSE)
dev.off()
```


```
null device 
          1
```


```
# # pdf
# pdf(file = here::here("3_results", "fig_corr.pdf"), height=6.5, width=6.5)
# corrplot(M, method="color", col=col(200),  
#          type="upper", 
#          addCoef.col = "black", # Add coefficient of correlation
#          tl.col="black", tl.srt=45, #Text label color and rotation
#          # Combine with significance
#          p.mat = p.mat, sig.level = 0.05, insig = "blank", 
#          # hide correlation coefficient on the principal diagonal
#          diag=FALSE)
# dev.off()
# 
# 
# # png
# png(file = here::here("3_results", "fig_corr.png"), height = 16.5, width = 16.5, units = 'cm', res = 500)
# corrplot(M, method="color", col=col(200),  
#          type="upper", 
#          addCoef.col = "black", # Add coefficient of correlation
#          tl.col="black", tl.srt=45, #Text label color and rotation
#          # Combine with significance
#          p.mat = p.mat, sig.level = 0.05, insig = "blank", 
#          # hide correlation coefficient on the principal diagonal
#          diag=FALSE)
# dev.off()
```

#### Way 2

Multicollinearity check: original data: psych


```
psych::pairs.panels(traits_merged[,4:12], gap = 0, bg = c(1:10)[factor(traits_merged$Species_ID)], pch = 21)
```


Multicollinearity check:log-transformed data: psych: does not change much and kept just for documentation


```
# psych::pairs.panels(log2(traits_merged[,4:12]), gap = 0, pch = 21, bg = c(1:10)[factor(traits_merged$Species_ID)])
```

#### Way 3

Multicollinearity check: original data: ggplot version


```
# GGally::ggpairs(traits_merged[,4:12])
```

## Discriminant analysis

The non-normal distribution and unequal variance are present. This prevents from using linear discriminant analysis or quadratic discriminant analysis. I will use flexible discriminant analysis.

My code is inspired by this post

Universal function for my discriminant analysis of fungal spores:


```
myFDA <- function(y, x, mydata, part, rep){
  # seed for reproducibility
  set.seed(12345)
 
  succ <- dim(rep)
  for (k in 1:rep) {
    
  # define train and test data
  training_samples <- caret::createDataPartition(mydata[, y], p = part, list = FALSE)  
  
  train_data <- mydata[training_samples, ]
  test_data <- mydata[-training_samples, ]
  
  # flexible discriminant analysis
  m <- mda::fda(paste(y, '~',  x), data = train_data)
  # table with predictions for test data
  tablin <- table(factor(test_data[, y]), predict(m, test_data))
  
  # count corrrect predictions = values that are not in the diagonal of "tablin"
  succ[k] <- sum(diag(tablin))/nrow(test_data)
  }
  return (mean(succ))
}
```


Now apply the function to particular spore traits:


```
# Symmetric shape variation
succ_S <- myFDA('Species_ID', 'PC1_symm_mean', mydata = traits_merged, part = 0.7, rep = 1000)

# Asymmetric shape variation
succ_A <- myFDA('Species_ID', 'PC1_asym_mean + PC2_asym_mean + PC3_asym_mean', mydata = traits_merged, part = 0.7, rep = 1000)

# Global shape variation
succ_G <- myFDA('Species_ID', 'PC1_glob_mean + PC2_glob_mean', mydata = traits_merged, part = 0.7, rep = 1000)

# Length + width
succ_LW <- myFDA('Species_ID', 'Length_mean + Width_mean', mydata = traits_merged, part = 0.7, rep = 1000)

#  Length to width ratio
succ_Q <- myFDA('Species_ID', 'Length_to_width_ratio_mean', mydata = traits_merged, part = 0.7, rep = 1000)

# Total variation as Q & size
succ_QLW <- myFDA('Species_ID', 'Length_to_width_ratio_mean + Length_mean + Width_mean', mydata = traits_merged, part = 0.7, rep = 1000)

# Total variation as global shape + size
succ_GLW <- myFDA('Species_ID', 'PC1_glob_mean + PC2_glob_mean + Length_mean + Width_mean', mydata = traits_merged, part = 0.7, rep = 1000)

# Total variation shape symmetric + asymmetric & size
succ_SALW <- myFDA('Species_ID', 'PC1_symm_mean + 
               PC1_asym_mean + PC2_asym_mean + PC3_asym_mean + 
               Length_mean + Width_mean', mydata = traits_merged, part = 0.7, rep = 1000)
```


All success rates in a single table


```
succ_all <- t(data.frame(succ_S,
                         succ_A,
                         succ_G,
                         succ_LW,
                         succ_Q,
                         succ_QLW,
                         succ_GLW,
                         succ_SALW))
succ_all
```


```
               [,1]
succ_S    0.5794741
succ_A    0.4689569
succ_G    0.6152328
succ_LW   0.5905603
succ_Q    0.5381552
succ_QLW  0.5941121
succ_GLW  0.6243362
succ_SALW 0.6469397
```


Re-format and plot the identification success rates


```
succ_all_df <- data.frame(succ_all)
# row.names(data.frame(succ_all)) # to retrieve the names of the predictors and adjust them
# succ_all_df$Predictors <- row.names(data.frame(succ_all))
succ_all_df$Predictors <- c('S', 'A', 'G', 'LW', 'Q', 'QLW',  'GLW', 'SALW')

names(succ_all_df)[1] <- 'Success' 
succ_all_df[1] <- round(succ_all_df[1]*100, 1) # switch to percents

# make predicors to be factors, instead of characters. This will keep their original order while plotting. 
succ_all_df$Predictors <- factor(succ_all_df$Predictors, levels = succ_all_df$Predictors)
p <- ggplot(succ_all_df, aes(x=reorder(Predictors, Success), y=Success)) +
  geom_bar(stat="identity", fill="steelblue") +
  xlab('Predictors') +
  ylab('Identification success rate, %') + 
  coord_flip(ylim = c(45, 65)) + 
  geom_text(aes(label=Success), vjust=0.5, hjust = 1.4, size = 4, color="white")
  theme(axis.title = element_text(size = 16), axis.text = element_text(size = 12))
```


```
List of 2
 $ axis.title:List of 11
  ..$ family       : NULL
  ..$ face         : NULL
  ..$ colour       : NULL
  ..$ size         : num 16
  ..$ hjust        : NULL
  ..$ vjust        : NULL
  ..$ angle        : NULL
  ..$ lineheight   : NULL
  ..$ margin       : NULL
  ..$ debug        : NULL
  ..$ inherit.blank: logi FALSE
  ..- attr(*, "class")= chr [1:2] "element_text" "element"
 $ axis.text :List of 11
  ..$ family       : NULL
  ..$ face         : NULL
  ..$ colour       : NULL
  ..$ size         : num 12
  ..$ hjust        : NULL
  ..$ vjust        : NULL
  ..$ angle        : NULL
  ..$ lineheight   : NULL
  ..$ margin       : NULL
  ..$ debug        : NULL
  ..$ inherit.blank: logi FALSE
  ..- attr(*, "class")= chr [1:2] "element_text" "element"
 - attr(*, "class")= chr [1:2] "theme" "gg"
 - attr(*, "complete")= logi FALSE
 - attr(*, "validate")= logi TRUE
```


```
p
```


Save id success rates plot


```
# tiff
tiff(file = here::here("3_results", "fig_success_1000rep.tiff"), height= 9, width=12, units = 'cm', res = 600,
     compression = "lzw", family = "sans")
print(p)
dev.off()
```


```
null device 
          1
```


```
# png(file = here::here("3_results", "bar_dis.png"), height= 9, width=12, units = 'cm', res = 300)
# print(p)
# dev.off()
```

## Phylogenetic tree that justifies the assignment of specimens to species

### Load DNA sequences to R memory


```
library(Biostrings)
conflicted::conflict_prefer("strsplit", "Biostrings")
seq <- readDNAStringSet(here::here("2_1_data_dna", "202103", "its_30seq.fasta"))
```


```
reading FASTA file C:/Users/aordy/OneDrive/Documents/Projects/ARTS/2_studies/1_size_vs_shape/3_latest/2_1_data_dna/202103/its_30seq.fasta: ignored 5 invalid one-letter sequence codes
```


```
# seq@ranges@NAMES # seq names are here, work on names later if necessary
```

### Multiple sequence alignment (MSA)


```
library(msa)
ali_XString <- msa::msa(seq, type="dna", method="Muscle")
#the run took 11.45 seconds for 48 ITS sequences on my Dell laptop in Aug 2020

library(ape)
# convert the alignment to DNAbin
ali_bin <- as.DNAbin(ali_XString)
```

### Plot MSA

#### Untrimmed MSA


```
par(mar=c(3,7,3,3))
image(ali_bin, cex.lab = 0.8)
```

#### Trimmed MSA

Trimm the ends of aligment and plot again


```
# package ips is required
# to make ips working properly, I had to install XML package from binary in the following way:
# install.packages("XML", type = "binary")
library(ips)
ali_trimmed <- trimEnds(ali_bin, min.n.seq = 15)
par(mar=c(3,10,3,9))
image(ali_trimmed, cex.lab = 0.8)
```

### Maximum Likelihood phylogenetic analysis


```
library(phangorn)
conflicted::conflict_prefer("rename", "S4Vectors")
```


```
[conflicted] Removing existing preference
[conflicted] Will prefer S4Vectors::rename over any other package
```


```
mt <- modelTest(phyDat(ali_trimmed), model="all", control = pml.control(trace = 0))
```


```
negative edges length changed to 0!
```


```
# choose best model to your preferred information criteria
bestModel <- mt$Model[which.min(mt$AIC)]
# "some R magic" - citation from Klaus Schliep
env <- attr(mt, "env")
fitStart <- eval(get(bestModel, env), env=env)
# optimize model
fit.nni <- optim.pml(fitStart, rearrangement="NNI", control = pml.control(trace = 0))
# bootstrap analysis
bs <- bootstrap.pml(fit.nni, bs=1000, optNni=TRUE, control = pml.control(trace = 0))
```


```
Bedingung hat L攼㸴nge > 1 und nur das erste Element wird benutztBedingung hat L攼㸴nge > 1 und nur das erste Element wird benutztBedingung hat L攼㸴nge > 1 und nur das erste Element wird benutzt
```


```
Final p-score 699 after  3 nni operations 
Final p-score 689 after  1 nni operations 
Final p-score 684 after  2 nni operations 
Final p-score 768 after  2 nni operations 
Final p-score 737 after  3 nni operations 
Final p-score 639 after  2 nni operations 
Final p-score 701 after  2 nni operations 
Final p-score 680 after  2 nni operations 
Final p-score 684 after  4 nni operations 
Final p-score 723 after  2 nni operations 
Final p-score 682 after  5 nni operations 
Final p-score 822 after  2 nni operations 
Final p-score 761 after  1 nni operations 
Final p-score 752 after  3 nni operations 
Final p-score 740 after  3 nni operations 
Final p-score 702 after  1 nni operations 
Final p-score 703 after  3 nni operations 
Final p-score 723 after  4 nni operations 
Final p-score 723 after  3 nni operations 
Final p-score 779 after  4 nni operations 
Final p-score 682 after  3 nni operations 
Final p-score 713 after  2 nni operations 
Final p-score 797 after  3 nni operations 
Final p-score 662 after  1 nni operations 
Final p-score 717 after  2 nni operations 
Final p-score 704 after  3 nni operations 
Final p-score 699 after  0 nni operations 
Final p-score 668 after  6 nni operations 
Final p-score 682 after  4 nni operations 
Final p-score 728 after  4 nni operations 
Final p-score 729 after  3 nni operations 
Final p-score 714 after  3 nni operations 
Final p-score 738 after  2 nni operations 
Final p-score 777 after  5 nni operations 
Final p-score 666 after  4 nni operations 
Final p-score 809 after  2 nni operations 
Final p-score 646 after  1 nni operations 
Final p-score 656 after  2 nni operations 
Final p-score 743 after  4 nni operations 
Final p-score 791 after  5 nni operations 
Final p-score 741 after  3 nni operations 
Final p-score 723 after  2 nni operations 
Final p-score 643 after  5 nni operations 
Final p-score 812 after  3 nni operations 
Final p-score 705 after  3 nni operations 
Final p-score 717 after  0 nni operations 
Final p-score 756 after  3 nni operations 
Final p-score 625 after  3 nni operations 
Final p-score 698 after  4 nni operations 
Final p-score 792 after  4 nni operations 
Final p-score 750 after  2 nni operations 
Final p-score 769 after  6 nni operations 
Final p-score 636 after  5 nni operations 
Final p-score 679 after  2 nni operations 
Final p-score 726 after  2 nni operations 
Final p-score 774 after  4 nni operations 
Final p-score 740 after  2 nni operations 
Final p-score 750 after  3 nni operations 
Final p-score 693 after  5 nni operations 
Final p-score 796 after  4 nni operations 
Final p-score 729 after  3 nni operations 
Final p-score 769 after  1 nni operations 
Final p-score 756 after  1 nni operations 
Final p-score 720 after  5 nni operations 
Final p-score 672 after  2 nni operations 
Final p-score 691 after  1 nni operations 
Final p-score 709 after  2 nni operations 
Final p-score 721 after  3 nni operations 
Final p-score 800 after  2 nni operations 
Final p-score 678 after  2 nni operations 
Final p-score 723 after  2 nni operations 
Final p-score 700 after  5 nni operations 
Final p-score 763 after  1 nni operations 
Final p-score 727 after  0 nni operations 
Final p-score 652 after  1 nni operations 
Final p-score 719 after  1 nni operations 
Final p-score 646 after  0 nni operations 
Final p-score 684 after  6 nni operations 
Final p-score 658 after  2 nni operations 
Final p-score 731 after  7 nni operations 
Final p-score 673 after  3 nni operations 
Final p-score 665 after  6 nni operations 
Final p-score 712 after  4 nni operations 
Final p-score 719 after  2 nni operations 
Final p-score 700 after  3 nni operations 
Final p-score 671 after  0 nni operations 
Final p-score 649 after  5 nni operations 
Final p-score 753 after  5 nni operations 
Final p-score 646 after  1 nni operations 
Final p-score 721 after  1 nni operations 
Final p-score 684 after  2 nni operations 
Final p-score 777 after  3 nni operations 
Final p-score 725 after  4 nni operations 
Final p-score 713 after  2 nni operations 
Final p-score 747 after  4 nni operations 
Final p-score 722 after  6 nni operations 
Final p-score 712 after  2 nni operations 
Final p-score 796 after  9 nni operations 
Final p-score 668 after  4 nni operations 
Final p-score 752 after  2 nni operations 
Final p-score 668 after  5 nni operations 
Final p-score 702 after  2 nni operations 
Final p-score 688 after  2 nni operations 
Final p-score 746 after  5 nni operations 
Final p-score 718 after  1 nni operations 
Final p-score 755 after  3 nni operations 
Final p-score 708 after  1 nni operations 
Final p-score 778 after  5 nni operations 
Final p-score 720 after  2 nni operations 
Final p-score 737 after  6 nni operations 
Final p-score 673 after  1 nni operations 
Final p-score 739 after  3 nni operations 
Final p-score 659 after  3 nni operations 
Final p-score 715 after  5 nni operations 
Final p-score 695 after  1 nni operations 
Final p-score 763 after  14 nni operations 
Final p-score 821 after  2 nni operations 
Final p-score 754 after  4 nni operations 
Final p-score 718 after  0 nni operations 
Final p-score 690 after  1 nni operations 
Final p-score 613 after  3 nni operations 
Final p-score 611 after  3 nni operations 
Final p-score 766 after  3 nni operations 
Final p-score 615 after  2 nni operations 
Final p-score 682 after  5 nni operations 
Final p-score 760 after  3 nni operations 
Final p-score 678 after  3 nni operations 
Final p-score 606 after  2 nni operations 
Final p-score 749 after  2 nni operations 
Final p-score 731 after  1 nni operations 
Final p-score 664 after  5 nni operations 
Final p-score 685 after  7 nni operations 
Final p-score 704 after  1 nni operations 
Final p-score 749 after  1 nni operations 
Final p-score 699 after  0 nni operations 
Final p-score 732 after  1 nni operations 
Final p-score 630 after  3 nni operations 
Final p-score 772 after  3 nni operations 
Final p-score 670 after  3 nni operations 
Final p-score 850 after  1 nni operations 
Final p-score 708 after  2 nni operations 
Final p-score 693 after  9 nni operations 
Final p-score 640 after  6 nni operations 
Final p-score 751 after  0 nni operations 
Final p-score 758 after  3 nni operations 
Final p-score 705 after  5 nni operations 
Final p-score 692 after  3 nni operations 
Final p-score 775 after  3 nni operations 
Final p-score 683 after  5 nni operations 
Final p-score 778 after  5 nni operations 
Final p-score 714 after  5 nni operations 
Final p-score 666 after  2 nni operations 
Final p-score 617 after  4 nni operations 
Final p-score 655 after  4 nni operations 
Final p-score 758 after  5 nni operations 
Final p-score 725 after  2 nni operations 
Final p-score 693 after  2 nni operations 
Final p-score 784 after  7 nni operations 
Final p-score 709 after  1 nni operations 
Final p-score 762 after  0 nni operations 
Final p-score 721 after  1 nni operations 
Final p-score 690 after  3 nni operations 
Final p-score 715 after  0 nni operations 
Final p-score 629 after  3 nni operations 
Final p-score 708 after  2 nni operations 
Final p-score 737 after  4 nni operations 
Final p-score 716 after  4 nni operations 
Final p-score 815 after  0 nni operations 
Final p-score 781 after  3 nni operations 
Final p-score 760 after  3 nni operations 
Final p-score 733 after  3 nni operations 
Final p-score 711 after  3 nni operations 
Final p-score 602 after  2 nni operations 
Final p-score 677 after  3 nni operations 
Final p-score 678 after  5 nni operations 
Final p-score 704 after  4 nni operations 
Final p-score 696 after  0 nni operations 
Final p-score 676 after  2 nni operations 
Final p-score 747 after  1 nni operations 
Final p-score 676 after  5 nni operations 
Final p-score 723 after  5 nni operations 
Final p-score 734 after  5 nni operations 
Final p-score 707 after  2 nni operations 
Final p-score 676 after  6 nni operations 
Final p-score 634 after  0 nni operations 
Final p-score 698 after  0 nni operations 
Final p-score 667 after  5 nni operations 
Final p-score 699 after  4 nni operations 
Final p-score 651 after  1 nni operations 
Final p-score 731 after  1 nni operations 
Final p-score 765 after  4 nni operations 
Final p-score 654 after  0 nni operations 
Final p-score 735 after  1 nni operations 
Final p-score 675 after  3 nni operations 
Final p-score 688 after  3 nni operations 
Final p-score 758 after  1 nni operations 
Final p-score 706 after  5 nni operations 
Final p-score 730 after  4 nni operations 
Final p-score 768 after  4 nni operations 
Final p-score 711 after  1 nni operations 
Final p-score 720 after  0 nni operations 
Final p-score 686 after  2 nni operations 
Final p-score 693 after  4 nni operations 
Final p-score 712 after  6 nni operations 
Final p-score 651 after  9 nni operations 
Final p-score 640 after  5 nni operations 
Final p-score 672 after  1 nni operations 
Final p-score 717 after  2 nni operations 
Final p-score 680 after  2 nni operations 
Final p-score 716 after  1 nni operations 
Final p-score 695 after  0 nni operations 
Final p-score 701 after  2 nni operations 
Final p-score 754 after  2 nni operations 
Final p-score 725 after  8 nni operations 
Final p-score 700 after  3 nni operations 
Final p-score 704 after  2 nni operations 
Final p-score 750 after  5 nni operations 
Final p-score 717 after  3 nni operations 
Final p-score 828 after  1 nni operations 
Final p-score 685 after  4 nni operations 
Final p-score 707 after  3 nni operations 
Final p-score 633 after  1 nni operations 
Final p-score 669 after  0 nni operations 
Final p-score 727 after  1 nni operations 
Final p-score 742 after  6 nni operations 
Final p-score 718 after  1 nni operations 
Final p-score 749 after  1 nni operations 
Final p-score 613 after  5 nni operations 
Final p-score 795 after  0 nni operations 
Final p-score 743 after  0 nni operations 
Final p-score 650 after  4 nni operations 
Final p-score 793 after  1 nni operations 
Final p-score 712 after  1 nni operations 
Final p-score 708 after  1 nni operations 
Final p-score 760 after  4 nni operations 
Final p-score 704 after  2 nni operations 
Final p-score 734 after  4 nni operations 
Final p-score 730 after  2 nni operations 
Final p-score 676 after  5 nni operations 
Final p-score 672 after  5 nni operations 
Final p-score 685 after  9 nni operations 
Final p-score 733 after  2 nni operations 
Final p-score 651 after  4 nni operations 
Final p-score 782 after  2 nni operations 
Final p-score 710 after  2 nni operations 
Final p-score 712 after  7 nni operations 
Final p-score 734 after  2 nni operations 
Final p-score 681 after  2 nni operations 
Final p-score 727 after  1 nni operations 
Final p-score 789 after  0 nni operations 
Final p-score 718 after  9 nni operations 
Final p-score 735 after  4 nni operations 
Final p-score 673 after  3 nni operations 
Final p-score 764 after  2 nni operations 
Final p-score 635 after  3 nni operations 
Final p-score 646 after  2 nni operations 
Final p-score 722 after  1 nni operations 
Final p-score 753 after  1 nni operations 
Final p-score 676 after  4 nni operations 
Final p-score 671 after  1 nni operations 
Final p-score 703 after  3 nni operations 
Final p-score 743 after  9 nni operations 
Final p-score 769 after  3 nni operations 
Final p-score 711 after  0 nni operations 
Final p-score 736 after  3 nni operations 
Final p-score 722 after  4 nni operations 
Final p-score 680 after  3 nni operations 
Final p-score 728 after  1 nni operations 
Final p-score 672 after  3 nni operations 
Final p-score 642 after  5 nni operations 
Final p-score 711 after  2 nni operations 
Final p-score 676 after  4 nni operations 
Final p-score 654 after  1 nni operations 
Final p-score 757 after  3 nni operations 
Final p-score 801 after  3 nni operations 
Final p-score 648 after  2 nni operations 
Final p-score 781 after  5 nni operations 
Final p-score 715 after  9 nni operations 
Final p-score 729 after  4 nni operations 
Final p-score 705 after  5 nni operations 
Final p-score 709 after  4 nni operations 
Final p-score 671 after  1 nni operations 
Final p-score 712 after  3 nni operations 
Final p-score 662 after  7 nni operations 
Final p-score 640 after  1 nni operations 
Final p-score 720 after  5 nni operations 
Final p-score 719 after  2 nni operations 
Final p-score 697 after  1 nni operations 
Final p-score 714 after  3 nni operations 
Final p-score 711 after  0 nni operations 
Final p-score 746 after  7 nni operations 
Final p-score 687 after  3 nni operations 
Final p-score 672 after  3 nni operations 
Final p-score 806 after  3 nni operations 
Final p-score 729 after  2 nni operations 
Final p-score 706 after  0 nni operations 
Final p-score 741 after  1 nni operations 
Final p-score 787 after  5 nni operations 
Final p-score 642 after  1 nni operations 
Final p-score 763 after  0 nni operations 
Final p-score 685 after  3 nni operations 
Final p-score 710 after  4 nni operations 
Final p-score 772 after  1 nni operations 
Final p-score 697 after  6 nni operations 
Final p-score 684 after  2 nni operations 
Final p-score 765 after  2 nni operations 
Final p-score 689 after  3 nni operations 
Final p-score 753 after  4 nni operations 
Final p-score 749 after  0 nni operations 
Final p-score 700 after  8 nni operations 
Final p-score 727 after  4 nni operations 
Final p-score 822 after  9 nni operations 
Final p-score 778 after  2 nni operations 
Final p-score 772 after  2 nni operations 
Final p-score 697 after  3 nni operations 
Final p-score 701 after  1 nni operations 
Final p-score 708 after  1 nni operations 
Final p-score 643 after  1 nni operations 
Final p-score 685 after  2 nni operations 
Final p-score 659 after  0 nni operations 
Final p-score 761 after  7 nni operations 
Final p-score 658 after  1 nni operations 
Final p-score 716 after  6 nni operations 
Final p-score 686 after  4 nni operations 
Final p-score 656 after  2 nni operations 
Final p-score 656 after  6 nni operations 
Final p-score 717 after  2 nni operations 
Final p-score 755 after  0 nni operations 
Final p-score 704 after  5 nni operations 
Final p-score 676 after  1 nni operations 
Final p-score 722 after  2 nni operations 
Final p-score 662 after  1 nni operations 
Final p-score 790 after  5 nni operations 
Final p-score 706 after  1 nni operations 
Final p-score 662 after  0 nni operations 
Final p-score 666 after  0 nni operations 
Final p-score 760 after  0 nni operations 
Final p-score 773 after  4 nni operations 
Final p-score 687 after  0 nni operations 
Final p-score 703 after  2 nni operations 
Final p-score 673 after  7 nni operations 
Final p-score 743 after  1 nni operations 
Final p-score 670 after  13 nni operations 
Final p-score 678 after  7 nni operations 
Final p-score 761 after  1 nni operations 
Final p-score 715 after  0 nni operations 
Final p-score 719 after  3 nni operations 
Final p-score 749 after  4 nni operations 
Final p-score 674 after  2 nni operations 
Final p-score 738 after  3 nni operations 
Final p-score 770 after  4 nni operations 
Final p-score 685 after  1 nni operations 
Final p-score 624 after  8 nni operations 
Final p-score 766 after  1 nni operations 
Final p-score 720 after  0 nni operations 
Final p-score 711 after  2 nni operations 
Final p-score 696 after  4 nni operations 
Final p-score 774 after  1 nni operations 
Final p-score 705 after  3 nni operations 
Final p-score 720 after  1 nni operations 
Final p-score 807 after  0 nni operations 
Final p-score 828 after  2 nni operations 
Final p-score 741 after  4 nni operations 
Final p-score 655 after  6 nni operations 
Final p-score 731 after  2 nni operations 
Final p-score 626 after  3 nni operations 
Final p-score 686 after  0 nni operations 
Final p-score 679 after  3 nni operations 
Final p-score 796 after  2 nni operations 
Final p-score 685 after  6 nni operations 
Final p-score 830 after  1 nni operations 
Final p-score 676 after  5 nni operations 
Final p-score 692 after  5 nni operations 
Final p-score 712 after  0 nni operations 
Final p-score 646 after  5 nni operations 
Final p-score 692 after  4 nni operations 
Final p-score 707 after  1 nni operations 
Final p-score 698 after  2 nni operations 
Final p-score 687 after  3 nni operations 
Final p-score 681 after  1 nni operations 
Final p-score 672 after  6 nni operations 
Final p-score 764 after  5 nni operations 
Final p-score 760 after  2 nni operations 
Final p-score 741 after  2 nni operations 
Final p-score 714 after  4 nni operations 
Final p-score 757 after  6 nni operations 
Final p-score 679 after  0 nni operations 
Final p-score 750 after  3 nni operations 
Final p-score 676 after  0 nni operations 
Final p-score 691 after  3 nni operations 
Final p-score 720 after  5 nni operations 
Final p-score 745 after  5 nni operations 
Final p-score 676 after  3 nni operations 
Final p-score 644 after  0 nni operations 
Final p-score 754 after  1 nni operations 
Final p-score 666 after  2 nni operations 
Final p-score 694 after  3 nni operations 
Final p-score 688 after  1 nni operations 
Final p-score 732 after  5 nni operations 
Final p-score 711 after  1 nni operations 
Final p-score 734 after  8 nni operations 
Final p-score 685 after  8 nni operations 
Final p-score 696 after  3 nni operations 
Final p-score 673 after  0 nni operations 
Final p-score 683 after  1 nni operations 
Final p-score 686 after  3 nni operations 
Final p-score 734 after  4 nni operations 
Final p-score 649 after  9 nni operations 
Final p-score 722 after  1 nni operations 
Final p-score 696 after  2 nni operations 
Final p-score 722 after  1 nni operations 
Final p-score 764 after  2 nni operations 
Final p-score 757 after  2 nni operations 
Final p-score 653 after  0 nni operations 
Final p-score 710 after  4 nni operations 
Final p-score 704 after  0 nni operations 
Final p-score 696 after  2 nni operations 
Final p-score 826 after  0 nni operations 
Final p-score 714 after  1 nni operations 
Final p-score 681 after  5 nni operations 
Final p-score 634 after  2 nni operations 
Final p-score 660 after  4 nni operations 
Final p-score 709 after  2 nni operations 
Final p-score 752 after  1 nni operations 
Final p-score 643 after  1 nni operations 
Final p-score 731 after  1 nni operations 
Final p-score 707 after  5 nni operations 
Final p-score 726 after  0 nni operations 
Final p-score 770 after  2 nni operations 
Final p-score 785 after  4 nni operations 
Final p-score 663 after  2 nni operations 
Final p-score 714 after  6 nni operations 
Final p-score 700 after  4 nni operations 
Final p-score 760 after  6 nni operations 
Final p-score 699 after  4 nni operations 
Final p-score 783 after  3 nni operations 
Final p-score 772 after  2 nni operations 
Final p-score 747 after  7 nni operations 
Final p-score 785 after  9 nni operations 
Final p-score 696 after  3 nni operations 
Final p-score 722 after  1 nni operations 
Final p-score 768 after  5 nni operations 
Final p-score 753 after  0 nni operations 
Final p-score 717 after  2 nni operations 
Final p-score 761 after  0 nni operations 
Final p-score 706 after  1 nni operations 
Final p-score 683 after  1 nni operations 
Final p-score 657 after  1 nni operations 
Final p-score 698 after  2 nni operations 
Final p-score 702 after  2 nni operations 
Final p-score 678 after  5 nni operations 
Final p-score 676 after  0 nni operations 
Final p-score 772 after  3 nni operations 
Final p-score 679 after  3 nni operations 
Final p-score 742 after  6 nni operations 
Final p-score 728 after  0 nni operations 
Final p-score 711 after  3 nni operations 
Final p-score 590 after  3 nni operations 
Final p-score 727 after  6 nni operations 
Final p-score 694 after  6 nni operations 
Final p-score 736 after  3 nni operations 
Final p-score 609 after  1 nni operations 
Final p-score 651 after  2 nni operations 
Final p-score 649 after  3 nni operations 
Final p-score 708 after  3 nni operations 
Final p-score 724 after  1 nni operations 
Final p-score 740 after  1 nni operations 
Final p-score 754 after  2 nni operations 
Final p-score 662 after  6 nni operations 
Final p-score 670 after  2 nni operations 
Final p-score 723 after  4 nni operations 
Final p-score 750 after  5 nni operations 
Final p-score 711 after  3 nni operations 
Final p-score 663 after  3 nni operations 
Final p-score 696 after  0 nni operations 
Final p-score 773 after  1 nni operations 
Final p-score 687 after  2 nni operations 
Final p-score 650 after  3 nni operations 
Final p-score 729 after  3 nni operations 
Final p-score 644 after  3 nni operations 
Final p-score 690 after  3 nni operations 
Final p-score 712 after  1 nni operations 
Final p-score 624 after  2 nni operations 
Final p-score 733 after  0 nni operations 
Final p-score 701 after  10 nni operations 
Final p-score 728 after  2 nni operations 
Final p-score 762 after  3 nni operations 
Final p-score 645 after  2 nni operations 
Final p-score 756 after  3 nni operations 
Final p-score 765 after  4 nni operations 
Final p-score 781 after  6 nni operations 
Final p-score 675 after  1 nni operations 
Final p-score 701 after  2 nni operations 
Final p-score 707 after  6 nni operations 
Final p-score 751 after  0 nni operations 
Final p-score 757 after  0 nni operations 
Final p-score 678 after  5 nni operations 
Final p-score 726 after  0 nni operations 
Final p-score 610 after  3 nni operations 
Final p-score 714 after  4 nni operations 
Final p-score 735 after  0 nni operations 
Final p-score 741 after  0 nni operations 
Final p-score 687 after  3 nni operations 
Final p-score 741 after  2 nni operations 
Final p-score 695 after  0 nni operations 
Final p-score 721 after  1 nni operations 
Final p-score 654 after  3 nni operations 
Final p-score 743 after  6 nni operations 
Final p-score 722 after  2 nni operations 
Final p-score 734 after  1 nni operations 
Final p-score 752 after  2 nni operations 
Final p-score 722 after  5 nni operations 
Final p-score 737 after  3 nni operations 
Final p-score 737 after  5 nni operations 
Final p-score 725 after  0 nni operations 
Final p-score 671 after  3 nni operations 
Final p-score 608 after  0 nni operations 
Final p-score 732 after  0 nni operations 
Final p-score 704 after  0 nni operations 
Final p-score 669 after  0 nni operations 
Final p-score 825 after  2 nni operations 
Final p-score 725 after  3 nni operations 
Final p-score 725 after  0 nni operations 
Final p-score 794 after  6 nni operations 
Final p-score 664 after  6 nni operations 
Final p-score 682 after  4 nni operations 
Final p-score 691 after  3 nni operations 
Final p-score 740 after  2 nni operations 
Final p-score 659 after  2 nni operations 
Final p-score 714 after  3 nni operations 
Final p-score 673 after  1 nni operations 
Final p-score 742 after  1 nni operations 
Final p-score 648 after  1 nni operations 
Final p-score 778 after  0 nni operations 
Final p-score 697 after  3 nni operations 
Final p-score 723 after  4 nni operations 
Final p-score 675 after  4 nni operations 
Final p-score 784 after  3 nni operations 
Final p-score 722 after  4 nni operations 
Final p-score 708 after  6 nni operations 
Final p-score 692 after  0 nni operations 
Final p-score 683 after  3 nni operations 
Final p-score 702 after  2 nni operations 
Final p-score 802 after  0 nni operations 
Final p-score 724 after  7 nni operations 
Final p-score 703 after  4 nni operations 
Final p-score 635 after  3 nni operations 
Final p-score 712 after  3 nni operations 
Final p-score 673 after  4 nni operations 
Final p-score 735 after  0 nni operations 
Final p-score 728 after  5 nni operations 
Final p-score 702 after  9 nni operations 
Final p-score 738 after  0 nni operations 
Final p-score 687 after  0 nni operations 
Final p-score 759 after  1 nni operations 
Final p-score 721 after  4 nni operations 
Final p-score 748 after  1 nni operations 
Final p-score 665 after  8 nni operations 
Final p-score 727 after  1 nni operations 
Final p-score 730 after  1 nni operations 
Final p-score 612 after  1 nni operations 
Final p-score 715 after  3 nni operations 
Final p-score 713 after  3 nni operations 
Final p-score 705 after  5 nni operations 
Final p-score 695 after  1 nni operations 
Final p-score 719 after  3 nni operations 
Final p-score 672 after  2 nni operations 
Final p-score 675 after  2 nni operations 
Final p-score 761 after  1 nni operations 
Final p-score 727 after  3 nni operations 
Final p-score 811 after  2 nni operations 
Final p-score 660 after  2 nni operations 
Final p-score 679 after  0 nni operations 
Final p-score 734 after  0 nni operations 
Final p-score 672 after  7 nni operations 
Final p-score 662 after  0 nni operations 
Final p-score 746 after  1 nni operations 
Final p-score 716 after  8 nni operations 
Final p-score 703 after  0 nni operations 
Final p-score 657 after  0 nni operations 
Final p-score 638 after  6 nni operations 
Final p-score 681 after  3 nni operations 
Final p-score 655 after  2 nni operations 
Final p-score 631 after  2 nni operations 
Final p-score 773 after  7 nni operations 
Final p-score 714 after  3 nni operations 
Final p-score 820 after  6 nni operations 
Final p-score 640 after  1 nni operations 
Final p-score 742 after  3 nni operations 
Final p-score 738 after  3 nni operations 
Final p-score 726 after  2 nni operations 
Final p-score 755 after  2 nni operations 
Final p-score 667 after  3 nni operations 
Final p-score 685 after  1 nni operations 
Final p-score 759 after  2 nni operations 
Final p-score 780 after  1 nni operations 
Final p-score 730 after  5 nni operations 
Final p-score 695 after  10 nni operations 
Final p-score 727 after  3 nni operations 
Final p-score 751 after  1 nni operations 
Final p-score 709 after  2 nni operations 
Final p-score 637 after  0 nni operations 
Final p-score 720 after  3 nni operations 
Final p-score 739 after  5 nni operations 
Final p-score 693 after  4 nni operations 
Final p-score 775 after  4 nni operations 
Final p-score 682 after  5 nni operations 
Final p-score 701 after  2 nni operations 
Final p-score 692 after  2 nni operations 
Final p-score 701 after  7 nni operations 
Final p-score 727 after  4 nni operations 
Final p-score 765 after  3 nni operations 
Final p-score 714 after  0 nni operations 
Final p-score 778 after  3 nni operations 
Final p-score 725 after  2 nni operations 
Final p-score 715 after  4 nni operations 
Final p-score 721 after  5 nni operations 
Final p-score 747 after  1 nni operations 
Final p-score 676 after  2 nni operations 
Final p-score 725 after  1 nni operations 
Final p-score 716 after  3 nni operations 
Final p-score 667 after  3 nni operations 
Final p-score 772 after  0 nni operations 
Final p-score 704 after  2 nni operations 
Final p-score 674 after  5 nni operations 
Final p-score 685 after  1 nni operations 
Final p-score 688 after  5 nni operations 
Final p-score 705 after  6 nni operations 
Final p-score 701 after  3 nni operations 
Final p-score 710 after  2 nni operations 
Final p-score 684 after  3 nni operations 
Final p-score 711 after  4 nni operations 
Final p-score 784 after  6 nni operations 
Final p-score 672 after  3 nni operations 
Final p-score 681 after  2 nni operations 
Final p-score 660 after  4 nni operations 
Final p-score 728 after  3 nni operations 
Final p-score 675 after  2 nni operations 
Final p-score 799 after  3 nni operations 
Final p-score 732 after  5 nni operations 
Final p-score 735 after  2 nni operations 
Final p-score 696 after  4 nni operations 
Final p-score 697 after  2 nni operations 
Final p-score 833 after  2 nni operations 
Final p-score 616 after  2 nni operations 
Final p-score 699 after  1 nni operations 
Final p-score 684 after  2 nni operations 
Final p-score 646 after  5 nni operations 
Final p-score 622 after  5 nni operations 
Final p-score 657 after  2 nni operations 
Final p-score 718 after  2 nni operations 
Final p-score 776 after  4 nni operations 
Final p-score 704 after  0 nni operations 
Final p-score 732 after  3 nni operations 
Final p-score 717 after  1 nni operations 
Final p-score 703 after  0 nni operations 
Final p-score 668 after  6 nni operations 
Final p-score 703 after  2 nni operations 
Final p-score 794 after  3 nni operations 
Final p-score 728 after  6 nni operations 
Final p-score 752 after  9 nni operations 
Final p-score 696 after  1 nni operations 
Final p-score 675 after  3 nni operations 
Final p-score 702 after  1 nni operations 
Final p-score 701 after  1 nni operations 
Final p-score 704 after  3 nni operations 
Final p-score 652 after  2 nni operations 
Final p-score 754 after  2 nni operations 
Final p-score 773 after  3 nni operations 
Final p-score 781 after  1 nni operations 
Final p-score 720 after  5 nni operations 
Final p-score 739 after  0 nni operations 
Final p-score 812 after  3 nni operations 
Final p-score 723 after  1 nni operations 
Final p-score 707 after  5 nni operations 
Final p-score 702 after  4 nni operations 
Final p-score 749 after  2 nni operations 
Final p-score 661 after  6 nni operations 
Final p-score 730 after  1 nni operations 
Final p-score 771 after  0 nni operations 
Final p-score 606 after  3 nni operations 
Final p-score 690 after  0 nni operations 
Final p-score 709 after  2 nni operations 
Final p-score 803 after  7 nni operations 
Final p-score 717 after  3 nni operations 
Final p-score 773 after  2 nni operations 
Final p-score 763 after  1 nni operations 
Final p-score 726 after  2 nni operations 
Final p-score 716 after  2 nni operations 
Final p-score 783 after  3 nni operations 
Final p-score 693 after  5 nni operations 
Final p-score 742 after  3 nni operations 
Final p-score 695 after  2 nni operations 
Final p-score 758 after  2 nni operations 
Final p-score 770 after  5 nni operations 
Final p-score 686 after  5 nni operations 
Final p-score 656 after  2 nni operations 
Final p-score 614 after  2 nni operations 
Final p-score 743 after  3 nni operations 
Final p-score 705 after  1 nni operations 
Final p-score 707 after  6 nni operations 
Final p-score 651 after  1 nni operations 
Final p-score 692 after  2 nni operations 
Final p-score 706 after  4 nni operations 
Final p-score 685 after  7 nni operations 
Final p-score 739 after  4 nni operations 
Final p-score 733 after  5 nni operations 
Final p-score 715 after  1 nni operations 
Final p-score 759 after  3 nni operations 
Final p-score 777 after  3 nni operations 
Final p-score 654 after  2 nni operations 
Final p-score 727 after  4 nni operations 
Final p-score 704 after  3 nni operations 
Final p-score 719 after  1 nni operations 
Final p-score 703 after  2 nni operations 
Final p-score 660 after  3 nni operations 
Final p-score 657 after  1 nni operations 
Final p-score 715 after  4 nni operations 
Final p-score 742 after  3 nni operations 
Final p-score 718 after  3 nni operations 
Final p-score 708 after  3 nni operations 
Final p-score 719 after  2 nni operations 
Final p-score 685 after  2 nni operations 
Final p-score 689 after  1 nni operations 
Final p-score 701 after  4 nni operations 
Final p-score 718 after  2 nni operations 
Final p-score 652 after  2 nni operations 
Final p-score 685 after  1 nni operations 
Final p-score 706 after  5 nni operations 
Final p-score 767 after  2 nni operations 
Final p-score 677 after  5 nni operations 
Final p-score 746 after  2 nni operations 
Final p-score 673 after  2 nni operations 
Final p-score 730 after  1 nni operations 
Final p-score 671 after  0 nni operations 
Final p-score 664 after  0 nni operations 
Final p-score 755 after  7 nni operations 
Final p-score 651 after  0 nni operations 
Final p-score 789 after  2 nni operations 
Final p-score 682 after  1 nni operations 
Final p-score 680 after  4 nni operations 
Final p-score 731 after  1 nni operations 
Final p-score 744 after  1 nni operations 
Final p-score 722 after  0 nni operations 
Final p-score 668 after  2 nni operations 
Final p-score 703 after  7 nni operations 
Final p-score 708 after  2 nni operations 
Final p-score 652 after  0 nni operations 
Final p-score 737 after  5 nni operations 
Final p-score 746 after  3 nni operations 
Final p-score 693 after  3 nni operations 
Final p-score 813 after  6 nni operations 
Final p-score 803 after  4 nni operations 
Final p-score 652 after  1 nni operations 
Final p-score 702 after  5 nni operations 
Final p-score 710 after  2 nni operations 
Final p-score 684 after  1 nni operations 
Final p-score 710 after  1 nni operations 
Final p-score 789 after  0 nni operations 
Final p-score 739 after  5 nni operations 
Final p-score 693 after  1 nni operations 
Final p-score 722 after  6 nni operations 
Final p-score 601 after  1 nni operations 
Final p-score 721 after  1 nni operations 
Final p-score 697 after  2 nni operations 
Final p-score 708 after  2 nni operations 
Final p-score 630 after  2 nni operations 
Final p-score 767 after  2 nni operations 
Final p-score 696 after  0 nni operations 
Final p-score 806 after  3 nni operations 
Final p-score 660 after  4 nni operations 
Final p-score 713 after  3 nni operations 
Final p-score 723 after  2 nni operations 
Final p-score 675 after  5 nni operations 
Final p-score 806 after  1 nni operations 
Final p-score 609 after  3 nni operations 
Final p-score 659 after  2 nni operations 
Final p-score 700 after  3 nni operations 
Final p-score 756 after  4 nni operations 
Final p-score 678 after  2 nni operations 
Final p-score 665 after  2 nni operations 
Final p-score 671 after  2 nni operations 
Final p-score 754 after  5 nni operations 
Final p-score 755 after  2 nni operations 
Final p-score 687 after  5 nni operations 
Final p-score 704 after  6 nni operations 
Final p-score 750 after  0 nni operations 
Final p-score 710 after  2 nni operations 
Final p-score 724 after  0 nni operations 
Final p-score 655 after  1 nni operations 
Final p-score 692 after  4 nni operations 
Final p-score 722 after  1 nni operations 
Final p-score 710 after  3 nni operations 
Final p-score 689 after  1 nni operations 
Final p-score 752 after  3 nni operations 
Final p-score 711 after  4 nni operations 
Final p-score 733 after  3 nni operations 
Final p-score 703 after  3 nni operations 
Final p-score 736 after  1 nni operations 
Final p-score 687 after  2 nni operations 
Final p-score 727 after  7 nni operations 
Final p-score 667 after  0 nni operations 
Final p-score 767 after  3 nni operations 
Final p-score 715 after  4 nni operations 
Final p-score 698 after  2 nni operations 
Final p-score 678 after  3 nni operations 
Final p-score 738 after  0 nni operations 
Final p-score 664 after  1 nni operations 
Final p-score 819 after  0 nni operations 
Final p-score 711 after  4 nni operations 
Final p-score 716 after  7 nni operations 
Final p-score 662 after  3 nni operations 
Final p-score 723 after  6 nni operations 
Final p-score 681 after  3 nni operations 
Final p-score 774 after  1 nni operations 
Final p-score 650 after  0 nni operations 
Final p-score 732 after  2 nni operations 
Final p-score 633 after  1 nni operations 
Final p-score 760 after  2 nni operations 
Final p-score 670 after  4 nni operations 
Final p-score 698 after  4 nni operations 
Final p-score 750 after  2 nni operations 
Final p-score 752 after  3 nni operations 
Final p-score 717 after  2 nni operations 
Final p-score 776 after  5 nni operations 
Final p-score 706 after  0 nni operations 
Final p-score 768 after  1 nni operations 
Final p-score 701 after  6 nni operations 
Final p-score 680 after  2 nni operations 
Final p-score 626 after  7 nni operations 
Final p-score 679 after  1 nni operations 
Final p-score 753 after  3 nni operations 
Final p-score 675 after  1 nni operations 
Final p-score 665 after  3 nni operations 
Final p-score 718 after  4 nni operations 
Final p-score 697 after  5 nni operations 
Final p-score 736 after  3 nni operations 
Final p-score 662 after  3 nni operations 
Final p-score 689 after  0 nni operations 
Final p-score 714 after  3 nni operations 
Final p-score 687 after  0 nni operations 
Final p-score 732 after  5 nni operations 
Final p-score 600 after  0 nni operations 
Final p-score 704 after  3 nni operations 
Final p-score 656 after  4 nni operations 
Final p-score 717 after  1 nni operations 
Final p-score 677 after  3 nni operations 
Final p-score 743 after  4 nni operations 
Final p-score 766 after  4 nni operations 
Final p-score 807 after  2 nni operations 
Final p-score 658 after  1 nni operations 
Final p-score 799 after  4 nni operations 
Final p-score 676 after  5 nni operations 
Final p-score 678 after  1 nni operations 
Final p-score 724 after  3 nni operations 
Final p-score 697 after  5 nni operations 
Final p-score 724 after  0 nni operations 
Final p-score 665 after  1 nni operations 
Final p-score 690 after  9 nni operations 
Final p-score 746 after  1 nni operations 
Final p-score 727 after  0 nni operations 
Final p-score 742 after  1 nni operations 
Final p-score 746 after  4 nni operations 
Final p-score 704 after  3 nni operations 
Final p-score 737 after  3 nni operations 
Final p-score 696 after  2 nni operations 
Final p-score 645 after  2 nni operations 
Final p-score 781 after  6 nni operations 
Final p-score 715 after  1 nni operations 
Final p-score 733 after  6 nni operations 
Final p-score 658 after  0 nni operations 
Final p-score 670 after  4 nni operations 
Final p-score 743 after  1 nni operations 
Final p-score 743 after  1 nni operations 
Final p-score 762 after  1 nni operations 
Final p-score 680 after  4 nni operations 
Final p-score 595 after  5 nni operations 
Final p-score 704 after  2 nni operations 
Final p-score 611 after  0 nni operations 
Final p-score 749 after  4 nni operations 
Final p-score 732 after  5 nni operations 
Final p-score 632 after  1 nni operations 
Final p-score 660 after  3 nni operations 
Final p-score 687 after  2 nni operations 
Final p-score 667 after  5 nni operations 
Final p-score 685 after  2 nni operations 
Final p-score 740 after  2 nni operations 
Final p-score 754 after  1 nni operations 
Final p-score 713 after  2 nni operations 
Final p-score 664 after  4 nni operations 
Final p-score 748 after  2 nni operations 
Final p-score 666 after  3 nni operations 
Final p-score 726 after  0 nni operations 
Final p-score 701 after  1 nni operations 
Final p-score 776 after  2 nni operations 
Final p-score 638 after  6 nni operations 
Final p-score 716 after  2 nni operations 
Final p-score 669 after  3 nni operations 
Final p-score 768 after  4 nni operations 
Final p-score 691 after  4 nni operations 
Final p-score 717 after  0 nni operations 
Final p-score 723 after  5 nni operations 
Final p-score 741 after  7 nni operations 
Final p-score 705 after  8 nni operations 
Final p-score 762 after  1 nni operations 
Final p-score 724 after  5 nni operations 
Final p-score 679 after  2 nni operations 
Final p-score 695 after  1 nni operations 
Final p-score 685 after  5 nni operations 
Final p-score 706 after  8 nni operations 
Final p-score 653 after  2 nni operations 
Final p-score 745 after  3 nni operations 
Final p-score 660 after  5 nni operations 
Final p-score 781 after  3 nni operations 
Final p-score 746 after  1 nni operations 
Final p-score 784 after  7 nni operations 
Final p-score 651 after  5 nni operations 
Final p-score 707 after  2 nni operations 
Final p-score 731 after  5 nni operations 
Final p-score 700 after  3 nni operations 
Final p-score 661 after  1 nni operations 
Final p-score 758 after  3 nni operations 
Final p-score 621 after  1 nni operations 
Final p-score 767 after  5 nni operations 
Final p-score 803 after  3 nni operations 
Final p-score 733 after  3 nni operations 
Final p-score 716 after  1 nni operations 
Final p-score 698 after  5 nni operations 
Final p-score 728 after  7 nni operations 
Final p-score 712 after  1 nni operations 
Final p-score 747 after  6 nni operations 
Final p-score 696 after  2 nni operations 
Final p-score 699 after  3 nni operations 
Final p-score 748 after  3 nni operations 
Final p-score 755 after  2 nni operations 
Final p-score 725 after  4 nni operations 
Final p-score 767 after  2 nni operations 
Final p-score 759 after  5 nni operations 
Final p-score 720 after  4 nni operations 
Final p-score 749 after  3 nni operations 
Final p-score 777 after  5 nni operations 
Final p-score 815 after  5 nni operations 
Final p-score 662 after  5 nni operations 
Final p-score 676 after  1 nni operations 
Final p-score 769 after  3 nni operations 
Final p-score 675 after  5 nni operations 
Final p-score 768 after  3 nni operations 
Final p-score 678 after  1 nni operations 
Final p-score 721 after  6 nni operations 
Final p-score 649 after  2 nni operations 
Final p-score 694 after  0 nni operations 
Final p-score 701 after  6 nni operations 
Final p-score 754 after  1 nni operations 
Final p-score 734 after  3 nni operations 
Final p-score 682 after  5 nni operations 
Final p-score 714 after  2 nni operations 
Final p-score 690 after  2 nni operations 
Final p-score 686 after  4 nni operations 
Final p-score 729 after  0 nni operations 
Final p-score 685 after  2 nni operations 
Final p-score 696 after  0 nni operations 
Final p-score 699 after  0 nni operations 
Final p-score 747 after  1 nni operations 
Final p-score 648 after  3 nni operations 
Final p-score 751 after  2 nni operations 
Final p-score 681 after  4 nni operations 
Final p-score 711 after  4 nni operations 
Final p-score 718 after  4 nni operations 
Final p-score 738 after  3 nni operations 
Final p-score 752 after  2 nni operations 
Final p-score 688 after  2 nni operations 
Final p-score 727 after  5 nni operations 
Final p-score 758 after  3 nni operations 
Final p-score 734 after  2 nni operations 
Final p-score 763 after  3 nni operations 
Final p-score 689 after  5 nni operations 
Final p-score 714 after  5 nni operations 
Final p-score 721 after  4 nni operations 
Final p-score 739 after  1 nni operations 
Final p-score 668 after  8 nni operations 
Final p-score 765 after  1 nni operations 
Final p-score 698 after  2 nni operations 
Final p-score 691 after  0 nni operations 
Final p-score 795 after  6 nni operations 
Final p-score 656 after  1 nni operations 
Final p-score 650 after  1 nni operations 
Final p-score 661 after  4 nni operations 
Final p-score 689 after  1 nni operations 
Final p-score 752 after  1 nni operations 
Final p-score 669 after  1 nni operations 
Final p-score 664 after  6 nni operations 
Final p-score 675 after  2 nni operations 
Final p-score 741 after  3 nni operations 
Final p-score 769 after  2 nni operations 
Final p-score 735 after  2 nni operations 
Final p-score 804 after  0 nni operations 
Final p-score 705 after  5 nni operations 
Final p-score 709 after  4 nni operations 
Final p-score 637 after  2 nni operations 
Final p-score 766 after  3 nni operations
```


```
# save the tree with bootstrap supports as node labels in phylo object
tree.bs <- plotBS(fit.nni$tree, bs, type="phylo")
```


What was the best substitution model?


```
bestModel
```


```
[1] "TrN+G+I"
```


Save the tree to file


```
ape::write.tree(tree.bs, file = here::here("3_results", "tree_1000bs.newick"))
```

### Custom plotting of the tree


```
# reroot the tree
tree.bs <- root(tree.bs, c("KHL_10272", "KHL_10780", "KHL_10813"), resolve.root = T)

library(ggtree)
```


```
Registered S3 method overwritten by 'treeio':
  method     from
  root.phylo ape 
ggtree v2.4.1  For help: https://yulab-smu.top/treedata-book/

If you use ggtree in published research, please cite the most appropriate paper(s):

- Guangchuang Yu. Using ggtree to visualize data on tree-like structures. Current Protocols in Bioinformatics, 2020, 69:e96. doi:10.1002/cpbi.96
- Guangchuang Yu, Tommy Tsan-Yuk Lam, Huachen Zhu, Yi Guan. Two methods for mapping and visualizing associated data on phylogeny using ggtree. Molecular Biology and Evolution 2018, 35(12):3041-3043. doi:10.1093/molbev/msy194
- Guangchuang Yu, David Smith, Huachen Zhu, Yi Guan, Tommy Tsan-Yuk Lam. ggtree: an R package for visualization and annotation of phylogenetic trees with their covariates and other associated data. Methods in Ecology and Evolution 2017, 8(1):28-36. doi:10.1111/2041-210X.12628
```


```
library(ggplot2)

# add species names if not already done in the morphometric part of the script
sp_names <- read.csv(here::here("3_results", "species_names.csv"),  sep=",", stringsAsFactors=FALSE)
# now a complex operator will add the species names to the object
# it aligns the species names to sequence labels correctly - I've checked this manually
# there will be an error message that the number of replaced elements does not equal the
# total number of elements in the labels, but it is OK because we only assign the species names to the 
# terminal node labels = tip labels, and not to internal node labels (which are bootstrap supports here)
p0 <- ggtree(tree.bs) %<+% sp_names
```


Now setting all aestetics


```
p1 <- p0 + 
  geom_tree(size=0.1) +
  geom_tiplab(aes(color = Species_ID), key_glyph = draw_key_point, size=2, align=F, hjust=-0.01) + 
  geom_text2(aes(subset = !isTip, label = round(as.numeric(label), digits = 0)), size = 2, vjust = -0.7, hjust = 1.2) + 
  geom_treescale(linesize = 0.5, fontsize = 2, x = 0.85, y = 16.5) + 
  geom_text(label="substitutions per site", x=1, y=16.0, 
            size = 6 / .pt, fontface = 'plain', family = 'sans', stat = "unique") + 
  # this 'stat = 'unique'' makes the text font the same as inner ggploting
  xlim(-0.04, 1.2) + 
  theme(legend.position=c(0.8, 0.81),
        legend.background = element_rect(), 
        legend.key = element_blank(), # removes the border
        legend.key.size = unit(0.4, 'cm'),# sets overall area/size of the legend 
        legend.text = element_text(size = 6), # text size 
        title = element_text(size = 7)) + 
  theme(plot.margin = unit(c(0.1, 0, 0.1, 0.2), "cm")) 
  # +labs(col="Subulicystidium \n species") # would change legend title
```


Print the tree to a file(s)


```
# tiff via tiff()
tiff(filename = here::here("3_results", "fig_tree_1000bs.tiff"), height= 12, width=9, units = 'cm', res = 600, 
     compression = "lzw", family = "sans")
print(p1)
dev.off()
```


```
null device 
          1
```


```
# tiff via ggsave
# ggsave(filename = here::here("3_results", "fig_tree_20BS.tiff"), plot = p1, height= 12, width=9, units = 'cm', dpi = 400)

# # pdf
# pdf(file = here::here("3_results", "fig_tree.pdf"), height=5.0, width=3.5)
# print(p1)
# dev.off()
# 
# # png
# png(file = here::here("3_results", "fig_tree.png"), height= 12, width=9, units = 'cm', res = 500)
# print(p1)
# dev.off()
```

## References

Generates the bibliography of all used R packages and R version, and a paragraph for the Methods part of thesis (or paper)


```
# package "report" has to be installed https://easystats.github.io/report/articles/report.html
report::report(sessionInfo())
```


```
Analyses were conducted using the R Statistical language (version 4.0.3; R Core Team, 2020) on Windows 10 x64 (build 19041), using the packages ggpubr (version 0.4.0; Alboukadel Kassambara, 2020), ggtree (version 2.4.1; Guangchuang Yu. Using ggtree to visualize data on tree-like structures. Current Protocols in Bioinformatics, 2020, 69:e96. doi: 10.1002/cpbi.96), S4Vectors (version 0.28.0; Pagès, Lawrence and Aboyoun, 2020), Biostrings (version 2.58.0; Pagès et al., 2020), ggplot2 (version 3.3.3; Wickham. ggplot2: Elegant Graphics for Data Analysis. Springer-Verlag New York, 2016.), conflicted (version 1.0.4; Hadley Wickham, 2019), stringr (version 1.4.0; Hadley Wickham, 2019), readr (version 1.4.0; Hadley Wickham and Jim Hester, 2020), dplyr (version 1.0.3; Hadley Wickham et al., 2021), ips (version 0.0.11; Heibl 2008 onwards. PHYLOCH: R language tree plotting tools and interfaces to diverse phylogenetic software packages. http://www.christophheibl.de/Rpackages.html.), XVector (version 0.30.0; Hervé Pagès and Patrick Aboyoun, 2020), here (version 1.0.1; Kirill Müller, 2020), IRanges (version 2.24.0; Lawrence M et al., 2013), data.table (version 1.13.4; Matt Dowle and Arun Srinivasan, 2020), BiocGenerics (version 0.36.0; Orchestrating high-throughput genomic analysis with Bioconductor. Huber, V.Carey, Gentleman, ..., Morgan Nature Methods, 2015:12, 115.), ape (version 5.4.1; Paradis & Schliep 2019. ape 5.0: an environment for modern phylogenetics and evolutionary analyses in Bioinformatics 35: 526-528.), phangorn (version 2.5.5; Schliep K.2011. phangorn: phylogenetic analysis in Bioinformatics, 27(4), corrplot (version 0.84; Taiyun Wei and Viliam Simko, 2017) and msa (version 1.22.0; Bodenhofer et al., 2015).

References
----------
  - Alboukadel Kassambara (2020). ggpubr: 'ggplot2' Based Publication Ready Plots. R package version 0.4.0. https://CRAN.R-project.org/package=ggpubr
  - Guangchuang Yu. Using ggtree to visualize data on tree-like structures. Current Protocols in Bioinformatics, 2020, 69:e96. doi: 10.1002/cpbi.96
  - H. Pagès, M. Lawrence and P. Aboyoun (2020). S4Vectors: Foundation of vector-like and list-like containers in Bioconductor. R package version 0.28.0. https://bioconductor.org/packages/S4Vectors
  - H. Pagès, P. Aboyoun, R. Gentleman and S. DebRoy (2020). Biostrings: Efficient manipulation of biological strings. R package version 2.58.0. https://bioconductor.org/packages/Biostrings
  - H. Wickham. ggplot2: Elegant Graphics for Data Analysis. Springer-Verlag New York, 2016.
  - Hadley Wickham (2019). conflicted: An Alternative Conflict Resolution Strategy. R package version 1.0.4. https://CRAN.R-project.org/package=conflicted
  - Hadley Wickham (2019). stringr: Simple, Consistent Wrappers for Common String Operations. R package version 1.4.0. https://CRAN.R-project.org/package=stringr
  - Hadley Wickham and Jim Hester (2020). readr: Read Rectangular Text Data. R package version 1.4.0. https://CRAN.R-project.org/package=readr
  - Hadley Wickham, Romain François, Lionel Henry and Kirill Müller (2021). dplyr: A Grammar of Data Manipulation. R package version 1.0.3. https://CRAN.R-project.org/package=dplyr
  - Heibl C. 2008 onwards. PHYLOCH: R language tree plotting tools and interfaces to diverse phylogenetic software packages. http://www.christophheibl.de/Rpackages.html.
  - Hervé Pagès and Patrick Aboyoun (2020). XVector: Foundation of external vector representation and manipulation in Bioconductor. R package version 0.30.0. https://bioconductor.org/packages/XVector
  - Kirill Müller (2020). here: A Simpler Way to Find Your Files. R package version 1.0.1. https://CRAN.R-project.org/package=here
  - Lawrence M, Huber W, Pag\`es H, Aboyoun P, Carlson M, et al. (2013) Software for Computing and Annotating Genomic Ranges. PLoS Comput Biol 9(8): e1003118. doi:10.1371/journal.pcbi.1003118
  - Matt Dowle and Arun Srinivasan (2020). data.table: Extension of `data.frame`. R package version 1.13.4. https://CRAN.R-project.org/package=data.table
  - Orchestrating high-throughput genomic analysis with Bioconductor. W. Huber, V.J. Carey, R. Gentleman, ..., M. Morgan Nature Methods, 2015:12, 115.
  - Paradis E. & Schliep K. 2019. ape 5.0: an environment for modern phylogenetics and evolutionary analyses in R. Bioinformatics 35: 526-528.
  - R Core Team (2020). R: A language and environment for statistical computing. R Foundation for Statistical Computing, Vienna, Austria. URL https://www.R-project.org/.
  - Schliep K.P. 2011. phangorn: phylogenetic analysis in R. Bioinformatics, 27(4) 592-593
  - Taiyun Wei and Viliam Simko (2017). R package "corrplot": Visualization of a Correlation Matrix (Version 0.84). Available from https://github.com/taiyun/corrplot
  - U. Bodenhofer, E. Bonatesta, C. Horejs-Kainrath, and S. Hochreiter (2015) msa: an R package for multiple sequence alignment. Bioinformatics 31(24):3997-9999. DOI: 10.1093/bioinformatics/btv176.
```


End of the script.

LS0tDQp0aXRsZTogIlNwb3JlIHNoYXBlIHZzLiBzaXplIg0Kb3V0cHV0OiBodG1sX25vdGVib29rDQotLS0NCg0KVGhpcyBpcyBhbiBSIE5vdGVib29rIGNvbnRhaW5pbmcgY29kZSBhbmQgcmVzdWx0cyBvZiBjb21wYXJpbmcgdGhlIHBlcmZvcm1hbmNlICBvZiBzaGFwZSBhbmQgc2l6ZSB0cmFpdHMgZm9yIGRpc2NyaW1pbmF0aW9uIG9mIHNwZWNpZXMgaW4gKlN1YnVsaWN5c3RpZGl1bSouIA0KICAgDQoqKk5ldyBpbiB0aGlzIHZlcnNpb24qKjogIA0KKiB0aGlzIGlzIGxhcmdlbHkgYSBkdXBsaWNhdGUgb2YgdGhlIHZlcnNpb24gMjAyMTAxMjkgYnV0IG5vdyBiYXNlZCBvbiB0aGUgKGZvciBzdXJlbmVzcykgcmUtcnVuIFBDQSBmcm9tIDE3LjAyLjIwMjEgIA0KKiByZW1vdmVkIHRoZSBub24tcmVsZXZhbnQgaGVyZSBkZXNjcmlwdGlvbiBvZiBob3cgdG8gY3JlYXRlIGEgc2luZ2xlIE5FRiBmaWxlLiANCiogbWFrZSBpbGx1c3RyYXRpb25zIGFuZCB0YWJsZXMgd3JpdGluZyB0byB0aGUgZmlsZXMgd2l0aCBkZXNpcmVkIGRpbWVuc2lvbnMsIGludG8ga2luZCBvZiAnb3V0JyBmb2xkZXIgIA0KICANCiAgDQoqKlRvIGRvIGluIHRoZSBuZXh0IHZlcnNpb24qKjogIA0KKiBpbXBsZW1lbnQgcmVwb3J0KCkgaW4gdGhlIGVuZCBvZiB0aGUgc2NyaXB0DQoqIG1vdmUgUENBIGFuYWx5c2lzIG9mIE5FRkQgdG8gUiAgDQoqIEkgbWF5IGFsc28gYWRqdXN0IGxvbmdpc3BvcnVtIG5hbWUgaW4gdGhlIG5leHQgYW5hbHlzaXMgdmVyc2lvbiB3aGVuIHRoZSBkYXRhIGZyb20gQ2hyaXN0aW5hIHdpbGwgYmUgY29taW5nLiBUaGVuIG15IGxvbmdpc3BvcnVtIHdpbGwgYmVjb21lIGxvbmdpc3BvcnVtXzcNCiAgDQogIA0KKipNb3N0IGltcG9ydGFudCBjaGFuZ2VzIGluIHRoZSBwYXN0Kio6IA0KKiBhbmFseXNlcyBvbiBpbWFnZS1sZXZlbCBkYXRhLCBOIG9mIG9ic2VydmF0aW9ucyA9IDQwMS4gRWFjaCBvYnNlcnZhdGlvbiBpcyBhIGRhdGEgZnJvbSBhIHNpbmdsZSBpbWFnZSwgd2l0aCBpbmZvcm1hdGlvbiBvbiBtaW4gMSBzcG9yZSBhbmQgbWF4IDQgc3BvcmVzICANCiogY3VzdG9tIGZ1bmN0aW9uIGZvciBkaXNjcmltaW5hbnQgYW5hbHlzaXMgdGhhdCBhbGxvd3MgdG8gc2hvcnRlbiB0aGUgc2NyaXB0ICANCiAgDQogIA0KIyMgU2V0dGluZyB0aGUgd29yaw0KIyMjIEFsbCBpcyBwcmUtc2V0IGNvcnJlY3RseSBub3cNCkFsbCBjb2RlLCBkYXRhIGFuZCByZXN1bHRzIGFyZSBvcmdhbml6ZWQgaW50byBSU3R1ZGlvIFIgcHJvamVjdC4gQWZ0ZXIgY2xpY2tpbmcgdGhlIC5ScHJvaiBmaWxlLCB0aGlzIE5vdGVib29rIHdpbGwgYmUgb3BlbmVkIGFuZCB3b3JraW5nIGRpcmVjdG9yeSB3aWxsIGJlIHVuZGVyc3Rvb2QgYnkgUiBjb3JyZWN0bHkgYXMgYSBkaXJlY3Rvcnkgd2hpY2ggaXMgcGFyZW50IGZvciB0aGUgY29kZSwgZGF0YSBhbmQgcmVzdWx0cyBkaXJlY3Rvcmllcy4gDQogIA0KYWRkaXRpb25hbGx5LCBsb2FkaW5nIHBhY2thZ2UgImhlcmUiIHNob3VsZCAgbm90aWZ5IG9uIHRoZSBjb3JyZWN0IHdvcmtpbmcgZGlyZWN0b3J5LiAiaGVyZSIgIHdpbGwgYmUgYWxzbyB2ZXJ5IGhlbHBmdWwgZm9yIGFjY2Vzc2luZyBmaWxlcyBpbiB0aGUgY2hpbGQgIGRpcmVjdG9yaWVzIGFsb25nIHRoZSBzY3JpcHQuDQpgYGB7cn0NCmxpYnJhcnkoaGVyZSkNCmxpYnJhcnkoY29uZmxpY3RlZCkNCmNvbmZsaWN0X3ByZWZlcigiaGVyZSIsICJoZXJlIikNCmNvbmZsaWN0ZWQ6OmNvbmZsaWN0X3ByZWZlcigicmVuYW1lIiwgImRwbHlyIikNCmBgYA0KICANCiAgDQojIyMgVGhpcyBpcyBob3cgaXQgd2FzIGVhcmxpZXINCkF0IHRoZSB2ZXJ5IHN0YXJ0LCBhc3NpZ24gaW4gUiBzdHVkaW8gYXMgYSB3b3JraW5nIGRpcmVjdG9yeSB0aGUgZGlyZWN0b3J5IHdoaWNoIGlzIHBhcmVudCBmb3IgdGhlIGNvZGUsIGRhdGEgYW5kIHJlc3VsdHMgZGlyZWN0b3JpZXMuIFRoZW4gKnNldF9oZXJlKiBmdW5jdGlvbiBvZiB0aGUgImhlcmUiIHBhY2thZ2Ugd2lsbCBwaWNrIHRoaXMgYXNzaWdubWVudCBieSBjcmVhdGluZyB0aGUgZmlsZSAuaGVyZS4gVGhlbiBhbGwgc3Vic2VxdWVudCBSIHNlc3Npb25zIG9uIHRoaXMgcHJvamVjdCB3aWxsIHN0YXJ0IHdpdGggdGhlIHJpZ2h0IGRpcmVjdG9yeS4gDQpUbyBzZWUgbW9yZSBleGFtcGxlcyBvZiAgImhlcmUiIHZpc2l0IHRoaXMgW2xpbmtdKGh0dHA6Ly9qZW5yaWNobW9uZC5yYmluZC5pby9wb3N0L3doZXJlLWlzLWhlcmUvKQ0KDQoNCg0KIyMgTG9hZCBhbmQgcHJvY2VlZCBzcG9yZSBzaGFwZSBkYXRhDQoNCiMjIyBTdW1tYXJpemUgc3ltbWV0cmljIHZhcmlhdGlvbg0KTG9hZCBhbmQgcHJvY2VzcyB0aGUgdGFibGUgd2l0aCBQQ0Egc2NvcmVzIG9mIHRoZSBzcG9yZXMgKHN5bW1ldHJpYyB2YXJpYXRpb24pDQpgYGB7cn0NCnNjb3Jlc19zeW1tX3JhdyA8LSByZWFkLmNzdihoZXJlKCIyXzNfZGF0YV9zaGFwZSIsICJwY2FfMjAyMTAyIiwgIjFfc3ltbSIsICIzMHNwZWNpbWVuc19jbGVhbl9zeW1tLnBjcyIpLCAgc2VwPSJcdCIpDQoNCiMgYWRkIGNvbHVtbnMgdG8gYmUgZmlsbGVkIGluIGxhdGVyIHdpdGggcmVsZXZhbnQgSURzDQpzY29yZXNfc3ltbV9yYXcgPC0gY2JpbmQoU3BlY2ltZW5fSUQgPSBOQSwgc2NvcmVzX3N5bW1fcmF3KQ0Kc2NvcmVzX3N5bW1fcmF3IDwtIGNiaW5kKEltYWdlX0lEID0gTkEsIHNjb3Jlc19zeW1tX3JhdykNCg0KIyBmaWxsIGluIGNvbHVtbiBTcGVjaW1lbl9JRCBiYXNlZCBvbiB0aGUgY29udGVudCBvZiB0aGUgY29sdW1uIERBVEFfTkFNRQ0KbGlicmFyeShzdHJpbmdyKQ0Kc3RyaW5ncyA8LSBjKCJeQVJBTiIsICJeQ1dVIiwgIl5LSEwiLCAiXkxSIiwgIl5MWSIsICJeT3JkeW5ldHMiKQ0Kc2NvcmVzX3N5bW1fcmF3JFNwZWNpbWVuX0lEIDwtIGlmZWxzZShzdHJfZGV0ZWN0KHNjb3Jlc19zeW1tX3JhdyREQVRBX05BTUUsIHBhc3RlKHN0cmluZ3MsIGNvbGxhcHNlID0gInwiKSksIGdzdWIoIl4oW15fXSpfW15fXSopXy4qJCIsICJcXDEiLCBzY29yZXNfc3ltbV9yYXckREFUQV9OQU1FKSwgZ3N1YigiXihbXl9dKl9bXl9dKl9bXl9dKilfLiokIiwgIlxcMSIsIHNjb3Jlc19zeW1tX3JhdyREQVRBX05BTUUpKQ0KDQojIGZpbGwgaW4gY29sdW1uIEltYWdlX0lEIGJhc2VkIG9uIHRoZSBjb250ZW50IG9mIHRoZSBjb2x1bW4gREFUQV9OQU1FDQpzY29yZXNfc3ltbV9yYXckSW1hZ2VfSUQgPC0gaWZlbHNlKHN0cl9kZXRlY3Qoc2NvcmVzX3N5bW1fcmF3JERBVEFfTkFNRSwgcGFzdGUoc3RyaW5ncywgY29sbGFwc2UgPSAifCIpKSwgZ3N1YigiXihbXl9dKl9bXl9dKl9bXl9dKl9bXl9dKilfLiokIiwgIlxcMSIsIHNjb3Jlc19zeW1tX3JhdyREQVRBX05BTUUpLCBnc3ViKCJeKFteX10qX1teX10qX1teX10qX1teX10qX1teX10qKV8uKiQiLCAiXFwxIiwgc2NvcmVzX3N5bW1fcmF3JERBVEFfTkFNRSkpDQpgYGANCg0KDQpQbG90IFBDQSBzY29yZXMgYXMgc2NhdHRlcnBsb3RzIChzeW1tZXRyaWMgdmFyaWF0aW9uKQ0KYGBge3J9DQpsaWJyYXJ5KGdncGxvdDIpDQpwY2Ffc3ltbV9wbG90PC1nZ3Bsb3Qoc2NvcmVzX3N5bW1fcmF3LCBhZXMoeD1TcGVjaW1lbl9JRCwgeT1QQzEsIGNvbG9yPVNwZWNpbWVuX0lEKSkgKw0KZ2VvbV9wb2ludCgpKw0KdGhlbWUoYXhpcy50ZXh0LnggPSBlbGVtZW50X3RleHQoYW5nbGUgPSA5MCkpDQpwbG90KHBjYV9zeW1tX3Bsb3QpDQpgYGANCg0KU3VtbWFyaXplIHN5bW1ldHJpYyB2YXJpYXRpb24gYXQgdGhlIGltYWdlIElEIGxldmVsDQpgLmdyb3VwcyA9ICdkcm9wJ2AgYXJndW1lbnQgaW4gYGBgc3VtbWFyaXNlKClgYGAgd2lsbCBoZWxwIHRvIHJlbW92ZSBhICJmcmllbmRseSB3YXJuaW5nIiAgDQpgYGAgYHN1bW1hcmlzZSgpYCB1bmdyb3VwaW5nIG91dHB1dCAob3ZlcnJpZGUgd2l0aCBgLmdyb3Vwc2AgYXJndW1lbnQpYGBgICANClRoZSBleHBsYW5hdGlvbiBpcyBhdmFpbGFibGUgW2hlcmVdKGh0dHBzOi8vc3RhY2tvdmVyZmxvdy5jb20vcXVlc3Rpb25zLzYyMTQwNDgzL2hvdy10by1pbnRlcnByZXQtZHBseXItbWVzc2FnZS1zdW1tYXJpc2UtcmVncm91cGluZy1vdXRwdXQtYnkteC1vdmVycmlkZSkNCmBgYHtyfQ0KbGlicmFyeShkcGx5cikNCnNjb3Jlc19zeW1tX3N1bW0gPC0gc2NvcmVzX3N5bW1fcmF3ICU+JSANCiAgZ3JvdXBfYnkoSW1hZ2VfSUQpICU+JSANCiAgc3VtbWFyaXNlKGFjcm9zcyhTcGVjaW1lbl9JRCwgZHBseXI6OmZpcnN0KSwNCiAgICAgICAgICAgIGFjcm9zcyhQQzEsIG1lYW4pLA0KICAgICAgICAgICAgLmdyb3VwcyA9ICdkcm9wJykNCnNjb3Jlc19zeW1tX3N1bW0gPC0gZHBseXI6OnJlbmFtZShzY29yZXNfc3ltbV9zdW1tLCBQQzFfc3ltbV9tZWFuID0gUEMxKQ0KYGBgDQoNCg0KDQojIyMgU3VtbWFyaXplIGFzeW1tZXRyaWMgdmFyaWF0aW9uDQpMb2FkIGFuZCBwcm9jZXNzIHRoZSB0YWJsZSB3aXRoIFBDQSBzY29yZXMgZm9yIHNwb3JlcyAoYXN5bW1ldHJpYyB2YXJpYXRpb24pDQoNCmBgYHtyfQ0Kc2NvcmVzX2FzeW1fcmF3IDwtIHJlYWQuY3N2KGhlcmUoIjJfM19kYXRhX3NoYXBlIiwgInBjYV8yMDIxMDIiLCAiMl9hc3ltIiwgIjMwc3BlY2ltZW5zX2NsZWFuX2FzeW0ucGNzIiksICBzZXA9Ilx0IikNCg0KIyBhZGQgY29sdW1ucyB0byBiZSBmaWxsZWQgaW4gbGF0ZXIgd2l0aCByZWxldmFudCBJRHMNCnNjb3Jlc19hc3ltX3JhdyA8LSBjYmluZChTcGVjaW1lbl9JRCA9IE5BLCBzY29yZXNfYXN5bV9yYXcpDQpzY29yZXNfYXN5bV9yYXcgPC0gY2JpbmQoSW1hZ2VfSUQgPSBOQSwgc2NvcmVzX2FzeW1fcmF3KQ0KDQojIGZpbGwgaW4gY29sdW1uIFNwZWNpbWVuX0lEIGJhc2VkIG9uIHRoZSBjb250ZW50IG9mIHRoZSBjb2x1bW4gREFUQV9OQU1FLg0KIyAic3RyaW5ncyIgb2JqZWN0IHdhcyBjcmVhdGVkIGFib3ZlIGFuZCBpcyB2YWxpZCBoZXJlIGFzIGlzLg0KIyBQYWNrYWdlICJzdHJpbmdyIiBpcyBuZWNlc3NhcnkgYW5kIHdhcyBsb2FkZWQgZWFybGllci4NCnNjb3Jlc19hc3ltX3JhdyRTcGVjaW1lbl9JRCA8LSBpZmVsc2Uoc3RyX2RldGVjdChzY29yZXNfYXN5bV9yYXckREFUQV9OQU1FLCBwYXN0ZShzdHJpbmdzLCBjb2xsYXBzZSA9ICJ8IikpLCBnc3ViKCJeKFteX10qX1teX10qKV8uKiQiLCAiXFwxIiwgc2NvcmVzX2FzeW1fcmF3JERBVEFfTkFNRSksIGdzdWIoIl4oW15fXSpfW15fXSpfW15fXSopXy4qJCIsICJcXDEiLCBzY29yZXNfYXN5bV9yYXckREFUQV9OQU1FKSkNCg0KIyBmaWxsIGluIGNvbHVtbiBJbWFnZV9JRCBiYXNlZCBvbiB0aGUgY29udGVudCBvZiB0aGUgY29sdW1uIERBVEFfTkFNRS4NCnNjb3Jlc19hc3ltX3JhdyRJbWFnZV9JRCA8LSBpZmVsc2Uoc3RyX2RldGVjdChzY29yZXNfYXN5bV9yYXckREFUQV9OQU1FLCBwYXN0ZShzdHJpbmdzLCBjb2xsYXBzZSA9ICJ8IikpLCBnc3ViKCJeKFteX10qX1teX10qX1teX10qX1teX10qKV8uKiQiLCAiXFwxIiwgc2NvcmVzX2FzeW1fcmF3JERBVEFfTkFNRSksIGdzdWIoIl4oW15fXSpfW15fXSpfW15fXSpfW15fXSpfW15fXSopXy4qJCIsICJcXDEiLCBzY29yZXNfYXN5bV9yYXckREFUQV9OQU1FKSkNCmBgYA0KDQpQbG90IFBDQSBzY29yZXMgYXMgc2NhdHRlcnBsb3RzIChhc3ltbWV0cmljIHZhcmlhdGlvbik6IFBDMSB2cyBQQzINCmBgYHtyfQ0KbGlicmFyeShnZ3Bsb3QyKQ0KcGNhX2FzeW1fcGxvdDwtZ2dwbG90KHNjb3Jlc19hc3ltX3JhdywgYWVzKHg9UEMxLCB5PVBDMiwgY29sb3I9U3BlY2ltZW5fSUQpKSArDQpnZW9tX3BvaW50KCkNCnBsb3QocGNhX2FzeW1fcGxvdCkNCmBgYA0KDQpQbG90dGluZyBQQ0Egc2NvcmVzIGFzIHNjYXR0ZXJwbG90cyAoYXN5bW1ldHJpYyB2YXJpYXRpb24pOiBQQzEgdnMgUEMzDQpgYGB7cn0NCmxpYnJhcnkoZ2dwbG90MikNCnBjYV9hc3ltX3Bsb3Q8LWdncGxvdChzY29yZXNfYXN5bV9yYXcsIGFlcyh4PVBDMSwgeT1QQzMsIGNvbG9yPVNwZWNpbWVuX0lEKSkgKw0KZ2VvbV9wb2ludCgpDQpwbG90KHBjYV9hc3ltX3Bsb3QpDQpgYGANCg0KU3VtbWFyaXplIGFzeW1tZXRyaWMgdmFyaWF0aW9uIGF0IGltYWdlIGxldmVsDQpgYGB7cn0NCmxpYnJhcnkoZHBseXIpDQpzY29yZXNfYXN5bV9zdW1tIDwtIHNjb3Jlc19hc3ltX3JhdyAlPiUgDQogIGdyb3VwX2J5KEltYWdlX0lEKSAlPiUgDQogIHN1bW1hcmlzZShQQzFfYXN5bV9tZWFuPW1lYW4oUEMxKSwNCiAgICAgICAgICAgIFBDMl9hc3ltX21lYW49bWVhbihQQzIpLA0KICAgICAgICAgICAgUEMzX2FzeW1fbWVhbj1tZWFuKFBDMyksIA0KICAgICAgICAgICAgLmdyb3VwcyA9ICdkcm9wJykNCmBgYA0KDQoNCiMjIyBTdW1tYXJpemUgZ2xvYmFsIHZhcmlhdGlvbg0KTG9hZCBhbmQgcHJvY2VzcyB0aGUgdGFibGUgd2l0aCBQQ0Egc2NvcmVzIGZvciBzcG9yZXMgKGdsb2JhbCB2YXJpYXRpb24pDQpgYGB7cn0NCnNjb3Jlc19nbG9iX3JhdyA8LSByZWFkLmNzdihoZXJlKCIyXzNfZGF0YV9zaGFwZSIsICJwY2FfMjAyMTAyIiwgIjNfZ2xvYiIsICIzMHNwZWNpbWVuc19jbGVhbl9nbG9iLnBjcyIpLCAgc2VwPSJcdCIpDQoNCiMgYWRkIGNvbHVtbnMgdG8gYmUgZmlsbGVkIGluIGxhdGVyIHdpdGggcmVsZXZhbnQgSURzDQpzY29yZXNfZ2xvYl9yYXcgPC0gY2JpbmQoU3BlY2ltZW5fSUQgPSBOQSwgc2NvcmVzX2dsb2JfcmF3KQ0Kc2NvcmVzX2dsb2JfcmF3IDwtIGNiaW5kKEltYWdlX0lEID0gTkEsIHNjb3Jlc19nbG9iX3JhdykNCg0KIyBmaWxsIGluIGNvbHVtbiBTcGVjaW1lbl9JRCBiYXNlZCBvbiBjb250ZW50IG9mIHRoZSBjb2x1bW4gREFUQV9OQU1FDQojICJzdHJpbmdzIiBvYmplY3Qgd2FzIGNyZWF0ZWQgYWJvdmUgYW5kIGlzIHZhbGlkIGhlcmUgYXMgaXMNCiMgUGFja2FnZSAic3RyaW5nciIgaXMgbmVjZXNzYXJ5IGFuZCB3YXMgbG9hZGVkIGVhcmxpZXINCnNjb3Jlc19nbG9iX3JhdyRTcGVjaW1lbl9JRCA8LSBpZmVsc2Uoc3RyX2RldGVjdChzY29yZXNfZ2xvYl9yYXckREFUQV9OQU1FLCBwYXN0ZShzdHJpbmdzLCBjb2xsYXBzZSA9ICJ8IikpLCBnc3ViKCJeKFteX10qX1teX10qKV8uKiQiLCAiXFwxIiwgc2NvcmVzX2dsb2JfcmF3JERBVEFfTkFNRSksIGdzdWIoIl4oW15fXSpfW15fXSpfW15fXSopXy4qJCIsICJcXDEiLCBzY29yZXNfZ2xvYl9yYXckREFUQV9OQU1FKSkNCg0Kc2NvcmVzX2dsb2JfcmF3JEltYWdlX0lEIDwtIGlmZWxzZShzdHJfZGV0ZWN0KHNjb3Jlc19nbG9iX3JhdyREQVRBX05BTUUsIHBhc3RlKHN0cmluZ3MsIGNvbGxhcHNlID0gInwiKSksIGdzdWIoIl4oW15fXSpfW15fXSpfW15fXSpfW15fXSopXy4qJCIsICJcXDEiLCBzY29yZXNfZ2xvYl9yYXckREFUQV9OQU1FKSwgZ3N1YigiXihbXl9dKl9bXl9dKl9bXl9dKl9bXl9dKl9bXl9dKilfLiokIiwgIlxcMSIsIHNjb3Jlc19nbG9iX3JhdyREQVRBX05BTUUpKQ0KYGBgDQoNClBsb3R0aW5nIFBDQSBzY29yZXMgYXMgc2NhdHRlcnBsb3RzIChnbG9iYWwgdmFyaWF0aW9uKTogUEMxIHZzIFBDMg0KYGBge3J9DQpsaWJyYXJ5KGdncGxvdDIpDQpwY2FfZ2xvYl9wbG90PC1nZ3Bsb3Qoc2NvcmVzX2dsb2JfcmF3LCBhZXMoeD1QQzEsIHk9UEMyLCBjb2xvcj1TcGVjaW1lbl9JRCkpICsNCmdlb21fcG9pbnQoKQ0KcGxvdChwY2FfZ2xvYl9wbG90KQ0KYGBgDQoNClN1bW1hcml6ZSBnbG9iYWwgdmFyaWF0aW9uIGF0IHRoZSBpbWFnZSBsZXZlbA0KYGBge3J9DQpsaWJyYXJ5KGRwbHlyKQ0Kc2NvcmVzX2dsb2Jfc3VtbSA8LSBzY29yZXNfZ2xvYl9yYXcgJT4lIA0KICBncm91cF9ieShJbWFnZV9JRCkgJT4lIA0KICBzdW1tYXJpc2UoUEMxX2dsb2JfbWVhbj1tZWFuKFBDMSksDQogICAgICAgICAgICBQQzJfZ2xvYl9tZWFuPW1lYW4oUEMyKSwgDQogICAgICAgICAgICAuZ3JvdXBzID0gJ2Ryb3AnKQ0KYGBgDQoNCiANCg0KIyMgTG9hZCBhbmQgcHJvY2VlZCBzcG9yZSBzaXplIGRhdGENClNwb3JlIHNpemUgZGF0YSBmcm9tIHNlcGFyYXRlIHNwZWNpbWVucyBnZXQgcG9vbGVkLiAgDQpJIHdpbGwgc3VwcHJlc3MgdGhlIHdhcm5pbmcgbWVzc2FnZSAiTWlzc2luZyBjb2x1bW4gbmFtZXMgZmlsbGVkIGluOiAnWDEnIFsxXSIgdGhhdCBpcyBnZW5lcmF0ZWQgYnkgInJlYWQuY3N2IiBmdW5jdGlvbiB3aGljaCBpcyBmb3JjZWQgdG8gZ2l2ZSBhIG5hbWUgdG8gdGhlIHVubmFtZWQgY29sdW1uLiANCmBgYHtyIG1lc3NhZ2UgPSBGQUxTRX0NCmxpYnJhcnkoaGVyZSkNCmxpYnJhcnkocmVhZHIpDQppbXBvcnQuc2l6ZSA8LSBkaXIoaGVyZSgiMl8yX2RhdGFfc2l6ZSIsICJ2MjAyMDEyMTUiKSwgcGF0dGVybiA9ICIqLmNzdiIsIGZ1bGwubmFtZXMgPSBUKQ0KZGF0YV9zaXplX3JhdyA8LSBzdXBwcmVzc1dhcm5pbmdzKHBseXI6OmxkcGx5KGltcG9ydC5zaXplLCByZWFkX2NzdikpDQpgYGANCg0KDQpUaGVuIHRoZSBzcHJlYWRzaGVldCBpcyB0cmFuc2Zvcm1lZCB0byBwdXQgbGVuZ3RoIGFuZCB3aWR0aCAgb2YgZWFjaCBzcG9yZSBpbnRvIHNlcGFyYXRlIGNvbHVtbnMgb2Ygb25lIHJvdy4gIA0KV2FybmluZyBtZXNzYWdlIGZyb20gZHBseXIgd2hlbiBiaW5kaW5nIGxlbmd0aCBhbmQgd2lkdGggY29sdW1ucyAiTmV3IG5hbWVzOiAqIFgxIC0+IFgxLi4uMS4uLiIgdGhhdCBhcHBlYXJzIGFmdGVyIHRoZSBjb2RlIGNodW5rIGNhbiBiZSBpZ25vcmVkLiAgDQpUaGVuLCBsZW5ndGggdG8gd2lkdGggcmF0aW8gaXMgYWRkZWQgYXMgYW4gYWRkaXRpb25hbCBjb2x1bW4uICAgDQpGaW5hbGx5LCBjb2x1bW4gIkltYWdlX0lEIiBpcyBjcmVhdGVkLiAgDQoNCmBgYHtyfQ0KbGlicmFyeShkcGx5cikNCiMgZmluZCBzcG9yZSBsZW5ndGhzDQpkYXRhX3NpemVfcmF3X29kZCA8LSBkYXRhX3NpemVfcmF3ICU+JSBkcGx5cjo6c2xpY2Uod2hpY2gocm93X251bWJlcigpICUlIDIgPT0gMSkpDQpjb2xuYW1lcyhkYXRhX3NpemVfcmF3X29kZClbd2hpY2gobmFtZXMoZGF0YV9zaXplX3Jhd19vZGQpID09ICJMYWJlbCIpXSA8LSAiUGhvdG9fSUQiDQoNCiMgZmluZCBzcG9yZSB3aWR0aHMNCmRhdGFfc2l6ZV9yYXdfZXZlbiA8LSBkYXRhX3NpemVfcmF3ICU+JSBkcGx5cjo6c2xpY2Uod2hpY2gocm93X251bWJlcigpICUlIDIgPT0gMCkpIA0KY29sbmFtZXMoZGF0YV9zaXplX3Jhd19ldmVuKVt3aGljaChuYW1lcyhkYXRhX3NpemVfcmF3X2V2ZW4pID09ICJMZW5ndGgiKV0gPC0gIldpZHRoIg0KDQojIGJpbmQgbGVuZ3RoIGFuZCB3aWR0aCB2YWx1ZXMgYW5kIGxlYXZlIGp1c3QgbmVjZXNzYXJ5IHZhbHVlcw0KZGF0YV9zaXplX3RpbnkgPC0gYmluZF9jb2xzKGRhdGFfc2l6ZV9yYXdfb2RkLCBkYXRhX3NpemVfcmF3X2V2ZW4pDQpkYXRhX3NpemVfdGlueV9zaW1wbGUgPC1kYXRhX3NpemVfdGlueVssIGMoIlBob3RvX0lEIiwgIkxlbmd0aCIsICJXaWR0aCIpXQ0KDQojIGFkZCBsZW5ndGggdG8gd2lkdGggcmF0aW8gYXMgYW4gYWRkaXRpb25hbCB0cmFpdA0KZGF0YV9zaXplX3Rpbnlfc2ltcGxlIDwtIHRyYW5zZm9ybShkYXRhX3NpemVfdGlueV9zaW1wbGUsIExlbmd0aF90b193aWR0aF9yYXRpbyA9IExlbmd0aCAvIFdpZHRoKQ0KZGF0YV9zaXplX3Rpbnlfc2ltcGxlJExlbmd0aF90b193aWR0aF9yYXRpbyA8LSByb3VuZChkYXRhX3NpemVfdGlueV9zaW1wbGUkTGVuZ3RoX3RvX3dpZHRoX3JhdGlvLCAyKQ0KDQojIGFkZCBjb2x1bW4gZm9yIGltYWdlIElEcw0KZGF0YV9zaXplX3Rpbnlfc2ltcGxlIDwtIGNiaW5kKEltYWdlX0lEID0gTkEsIGRhdGFfc2l6ZV90aW55X3NpbXBsZSkNCg0KIyBmaWxsIHRoZSBjb2x1bW4gZm9yIGltYWdlIElEcw0KIyMgY29kZSB0aGF0IHVzZXMgcmVnZXggaXMgYmFzZWQgb24gdGhlIHNvbHV0aW9uIGZvciBnc3ViIA0KIyMgIyMgaHR0cHM6Ly9zdGFja292ZXJmbG93LmNvbS9xdWVzdGlvbnMvMzkzNjY3NTkvcmVnZXgtdG8tZXh0cmFjdC12YWx1ZXMtYmV0d2Vlbi0yLXVuZGVyc2NvcmVzLWluY2x1ZGluZy1hLXZhbHVlLXRoYXQtaXMtYW4tdW5kZQ0KIyMgaHR0cHM6Ly9zdGFja292ZXJmbG93LmNvbS9xdWVzdGlvbnMvNzQ0OTU2NC9yZWdleC1yZXR1cm4tYWxsLWJlZm9yZS10aGUtc2Vjb25kLW9jY3VycmVuY2UgDQoNCmxpYnJhcnkoc3RyaW5ncikNCnN0cmluZ3MgPC0gYygiXkFSQU4iLCAiXkNXVSIsICJeS0hMIiwgIl5MUiIsICJeTFkiLCAiXk9yZHluZXRzIikNCmRhdGFfc2l6ZV90aW55X3NpbXBsZSRJbWFnZV9JRCA8LSBpZmVsc2Uoc3RyX2RldGVjdChkYXRhX3NpemVfdGlueV9zaW1wbGUkUGhvdG9fSUQsIHBhc3RlKHN0cmluZ3MsIGNvbGxhcHNlID0gInwiKSksIGdzdWIoIl4oW15fXSpfW15fXSpfW15fXSpfW15fXSopXy4qJCIsICJcXDEiLCBzY29yZXNfc3ltbV9yYXckREFUQV9OQU1FKSwgZ3N1YigiXihbXl9dKl9bXl9dKl9bXl9dKl9bXl9dKl9bXl9dKilfLiokIiwgIlxcMSIsIGRhdGFfc2l6ZV90aW55X3NpbXBsZSRQaG90b19JRCkpDQoNCiNyZW1vdmUgIi5ibXAiIHN0cmluZyBwYXR0ZXJuIGZyb20gc29tZSBpbWFnZSBJRHMgDQpkYXRhX3NpemVfdGlueV9zaW1wbGUkSW1hZ2VfSUQgPC0gZ3N1YihwYXR0ZXJuID0gIi5ibXAqIiwgcmVwbGFjZW1lbnQgPSAiIiwgeCA9IGRhdGFfc2l6ZV90aW55X3NpbXBsZSRJbWFnZV9JRCkNCmBgYA0KDQpTdW1tYXJpemUgc2l6ZSBkYXRhIGF0IHRoZSBpbWFnZSBsZXZlbCANCmBgYHtyfQ0KbGlicmFyeShkcGx5cikgDQpzaXplX3N1bW0gPC0gZGF0YV9zaXplX3Rpbnlfc2ltcGxlICU+JSANCiAgZ3JvdXBfYnkoSW1hZ2VfSUQpICU+JSANCiAgc3VtbWFyaXNlKExlbmd0aF9tZWFuPW1lYW4oTGVuZ3RoKSwNCiAgICAgICAgICAgIFdpZHRoX21lYW49bWVhbihXaWR0aCksDQogICAgICAgICAgICBMZW5ndGhfdG9fd2lkdGhfcmF0aW9fbWVhbj1tZWFuKExlbmd0aF90b193aWR0aF9yYXRpbyksDQogICAgICAgICAgICBOX3Nwb3JlcyA9IG4oKSwNCiAgICAgICAgICAgICAgLmdyb3VwcyA9ICdkcm9wJykNCmBgYA0KDQpUaGUgY2h1bmsgYmVsb3cgYWxsb3dzIHRvIGFkZCBhIHZhcmlhYmxlIHdpdGggYW4gYWx0ZXJuYXRpdmUgc2l6ZSBkZWZpbml0aW9uLCBmb2xsb3dpbmcgQ2xhdWRlIDIwMDggYm9vayAocC4gOTgtOTkpLiAgDQpJdCBpcyBhIHBvc3NpYmxlIGFsdGVybmF0aXZlIHRvIHRoZSBzaW5nbGUgbGVuZ3RoIGFuZCB3aWR0aCBtZWFzdXJlbWVudHMuICANClRoaXMgbWVhc3VyZSBvZiBzaXplIHdvdWxkIGNvcnJlbGF0ZSBsZXNzIHdpdGggc2hhcGUgdmFyaWFibGVzLCB3aGljaCBpcyBhIGRlc2lyZWQgYmVoYXZpb3IuICANCkhvd2V2ZXIsIGlmIHVzZWQgaW4gaW4gZGlzY3JpbWluYW50IGFuYWx5c2lzLCBpdCBkaWQgbm90IGFsbG93IHRvIGdhaW4gaW4gc3BlY2llcyBwcmVkaWN0aW9uICh0cmllZCBpbiBGZWIgMjAyMCwgbm90IHNob3duIGluIHRoZSBjb2RlIGJlbG93IGJ1dCBjYW4gYmUgZWFzaWx5IGFkZGVkKS4gDQpgYGB7cn0NCiMjIG5lZWRzIGRwbHlyDQojIHNpemVfc3VtbSRTaXplX3NjYWxlZCA8LSBOQQ0KIyBzaXplX3N1bW0kU2l6ZV9zY2FsZWQgPC0gc3FydChzaXplX3N1bW0kTGVuZ3RoX21lYW4gKiBzaXplX3N1bW0kV2lkdGhfbWVhbikNCiMgc2l6ZV9zdW1tIDwtIHNpemVfc3VtbSAlPiUgcmVsb2NhdGUoU2l6ZV9zY2FsZWQsIC5iZWZvcmUgPSAnTGVuZ3RoX21lYW4nKSAjIGNvc21ldGljIGNoYW5nZXMgDQpgYGANCg0KDQoNCiMjIFVuaXRlIHNoYXBlIGFuZCBzaXplIGRhdGEgIA0KDQojIyMgQ2hlY2sgdGhhdCBJbWFnZXMgSURzIGFyZSBpbiB0aGUgc2FtZSBvcmRlciBpbiBhbGwgb2JqZWN0cy4gVGhleSB3aWxsIHNlcnZlIGFzIGdyb3VwaW5nIHZhcmlhYmxlDQpDaGVjayBmb3IgaW1hZ2UgbGV2ZWwgZGF0YSAobj00MDEgaW4gRGV6IDIwMjApLiBIZXJlLCBubyBuZWVkIHRvIHNvcnQgdGhlIGRhdGEgaW4gZWFjaCBncm91cCBiZWNhdXNlIHRoZXkgYXJlIGFsbCBpbiB0aGUgc2FtIG9yZGVyIGFmdGVyIGFwcGx5aW5nIGBgYHN1bW1hcmlzZSgpYGBgIGZ1bmN0aW9uDQpgYGB7cn0NCnNhcHBseShsaXN0KHNjb3Jlc19zeW1tX3N1bW0kSW1hZ2VfSUQsIHNjb3Jlc19hc3ltX3N1bW0kSW1hZ2VfSUQsIHNjb3Jlc19nbG9iX3N1bW0kSW1hZ2VfSUQpLA0KICAgICAgICAgICAgRlVOID0gYWxsLmVxdWFsLmxpc3QsIHNpemVfc3VtbSRJbWFnZV9JRCkNCmBgYA0KDQoNCiMjIyBDcmVhdGUgYSB1bml0ZWQgdGFibGUgd2l0aCBhbGwgdHJhaXRzICANCkpvaW4gc2VwYXJhdGUgZGF0YWZyYW1lcw0KYGBge3J9DQpsaWJyYXJ5KGRwbHlyKQ0KdHJhaXRzX21lcmdlZCA8LSBzY29yZXNfc3ltbV9zdW1tICU+JSByaWdodF9qb2luKHNjb3Jlc19hc3ltX3N1bW0sIGJ5ID0gIkltYWdlX0lEIikgJT4lIHJpZ2h0X2pvaW4oc2NvcmVzX2dsb2Jfc3VtbSwgYnkgPSAiSW1hZ2VfSUQiKSAlPiUgcmlnaHRfam9pbihzaXplX3N1bW0sIGJ5ID0gIkltYWdlX0lEIikNCmBgYA0KDQpJbiB0aGlzIHdheSAoY2h1bmsgYmVsb3cpIEkgZXhwb3J0ZWQgc3BlY2ltZW4gSURzIChuID0gMzApIGFuZCBhc3NpZ25lZCB0aGVtIG1hbnVhbHkgdG8gc3BlY2llcyBmb3IgZnVydGhlciBhbmFseXNpcy4NCklmIHRoZSBuZXcgc3BlY2ltZW5zIHdpbGwgYmUgYWRkZWQgdG8gdGhlIGRhdGFzZXQgYWZ0ZXIgc3VtbWVyIDIwMjAsIHRoZSB0YWJsZSB3aXRoIHNwZWNpZXMgbmFtZSBsYWJlbHMgc2hvdWxkIGJlIHVwZGF0ZWQuIA0KYGBge3J9DQojIG5vdCBydW4NCnJlYWRyOjp3cml0ZV9jc3YoYXMuZGF0YS5mcmFtZSh0YWJsZSh0cmFpdHNfbWVyZ2VkJFNwZWNpbWVuX0lEKSksIGZpbGUgPSBoZXJlKCIzX3Jlc3VsdHMiLCAic3BlY2llc19uYW1lc19yYXcuY3N2IikpDQpgYGANCg0KQWRkIGEgY29sdW1uIHdpdGggc3BlY2llcyBuYW1lcyBiYXNlZCBvbiBkYXRhIGZyb20gYW5vdGhlciB0YWJsZQ0KYGBge3J9DQp0cmFpdHNfbWVyZ2VkIDwtIGNiaW5kKFNwZWNpZXNfSUQgPSBOQSwgdHJhaXRzX21lcmdlZCkNCnRyYWl0c19tZXJnZWQkU3BlY2llc19JRCA8LSB0cmFpdHNfbWVyZ2VkJFNwZWNpbWVuX0lEDQpzcF9uYW1lcyA8LSByZWFkLmNzdihoZXJlKCIzX3Jlc3VsdHMiLCAic3BlY2llc19uYW1lcy5jc3YiKSwgIHNlcD0iLCIsIHN0cmluZ3NBc0ZhY3RvcnM9RkFMU0UpDQoNCmxpYnJhcnkoZGF0YS50YWJsZSkNCnRyYWl0c19tZXJnZWRfZHQgPC0gZGF0YS50YWJsZSh0cmFpdHNfbWVyZ2VkKQ0KdHJhaXRzX21lcmdlZF9kdFssIFNwZWNpZXNfSUQgOj0gYXMuY2hhcmFjdGVyKGZhY3RvcihTcGVjaWVzX0lELCBsYWJlbHMgPSBzcF9uYW1lcyRTcGVjaWVzX0lEKSldDQp0cmFpdHNfbWVyZ2VkIDwtIGRhdGEuZnJhbWUodHJhaXRzX21lcmdlZF9kdCkNCmBgYA0KDQpFeHBvcnQgdGhlIHRhYmxlIHdpdGggdHJhaXRzIG9uIGltYWdlIGxldmVsIGZvciBzdXBwbGVtZW50YXJ5DQpgYGB7cn0NCnJlYWRyOjp3cml0ZV9jc3YodHJhaXRzX21lcmdlZCwgZmlsZSA9IGhlcmUoIjNfcmVzdWx0cyIsICJ0cmFpdHNfcGVyX2ltYWdlLmNzdiIpKQ0KYGBgDQoNCg0KDQojIyAgRGF0YSBleHBsb3JhdGlvbg0KDQojIyMgU2NhdHRlcnBsb3RzIGZvciBzZXBhcmF0ZSB0cmFpdHMNCmBgYHtyfQ0KbGlicmFyeShnZ3Bsb3QyKQ0KIyBWaXN1YWxpemUgYXN5bW1ldHJpYyB2YXJpYXRpb24gYXQgc3BlY2ltZW4gbGV2ZWw6IFBDMSB2cyBQQzINCmdnX2FzeW1fUEMxdlBDMiA8LSBnZ3Bsb3QodHJhaXRzX21lcmdlZCwgYWVzKHg9UEMxX2FzeW1fbWVhbiwgeT1QQzJfYXN5bV9tZWFuLCBjb2xvcj1TcGVjaWVzX0lEKSkgKw0KICBnZW9tX3BvaW50KHNpemUgPSAxLCBhbHBoYSA9IDAuNykgKw0KICBsYWJzKHRhZyA9ICJBIiwgeCA9ICJQQzEgYXN5bW1ldHJpYyIsIHkgPSAiUEMyIGFzeW1tZXRyaWMiLCBjb2xvciA9IlNwZWNpZXMiKQ0KDQojIFZpc3VhbGl6ZSBhc3ltbWV0cmljIHZhcmlhdGlvbiBhdCBzcGVjaW1lbiBsZXZlbDogUEMxIHZzIFBDMw0KZ2dfYXN5bV9QQzF2UEMzIDwtIGdncGxvdCh0cmFpdHNfbWVyZ2VkLCBhZXMoeD1QQzFfYXN5bV9tZWFuLCB5PVBDM19hc3ltX21lYW4sIGNvbG9yPVNwZWNpZXNfSUQpKSArDQogIGdlb21fcG9pbnQoc2l6ZSA9IDEsIGFscGhhID0gMC43KSArIA0KICBsYWJzKHRhZyA9ICJCIiwgeCA9ICJQQzEgYXN5bW1ldHJpYyIsIHkgPSAiUEMzIGFzeW1tZXRyaWMiKQ0KDQojIFZpc3VhbGl6ZSBzeW1tZXRyaWMgc2hhcGUgdmFyaWF0aW9uIGF0IHNwZWNpbWVuIGxldmVsOiBQQzENCiMgTm90ZSB0aGF0IHRoZSB2YWx1ZXMgb24gYXhpcyB5IChhY3R1YWxseSB4IGJlZm9yZSBmbGlwcGluZykgYXJlIGluIHJldmVyc2Ugb3JkZXIgZm9yIGNvbXBhcmFiaWxpdHkgd2l0aCB0aGUgdmFsdWVzIGZvciBRIA0KZ2dfc3ltbV9QQzEgPC0gZ2dwbG90KHRyYWl0c19tZXJnZWQsIGFlcyh4PVBDMV9zeW1tX21lYW4sIGNvbG9yPVNwZWNpZXNfSUQpKSArDQpnZW9tX2JveHBsb3QoKSArDQpjb29yZF9mbGlwKCkgKw0Kc2NhbGVfeF9yZXZlcnNlKCkgKyANCmxhYnModGFnID0gIkMiLCB4ID0gIlBDMSBzeW1tZXRyaWMiKQ0KDQojIFZpc3VhbGl6ZSBnbG9iYWwgdmFyaWF0aW9uIGF0IHNwZWNpbWVuIGxldmVsOiBQQzEgdnMgUEMyDQpnZ19nbG9iX1BDMXZQQzIgPC1nZ3Bsb3QodHJhaXRzX21lcmdlZCwgYWVzKHg9UEMxX2dsb2JfbWVhbiwgeT1QQzJfZ2xvYl9tZWFuLCBjb2xvcj1TcGVjaWVzX0lEKSkgKyANCiAgZ2VvbV9wb2ludChzaXplID0gMSwgYWxwaGEgPSAwLjcpICsNCiAgbGFicyh0YWcgPSAiRCIsIHggPSAiUEMxIGdsb2JhbCIsIHkgPSAiUEMyIGdsb2JhbCIpDQoNCiMgVmlzdWFsaXplIHNpemUgdmFyaWF0aW9uIGF0IHNwZWNpbWVuIGxldmVsOiBMZW5ndGggdnMgd2lkdGgNCmdnX3NpemVfTHZXIDwtZ2dwbG90KHRyYWl0c19tZXJnZWQsIGFlcyh4PUxlbmd0aF9tZWFuLCB5PVdpZHRoX21lYW4sIGNvbG9yPVNwZWNpZXNfSUQpKSArDQogIGdlb21fcG9pbnQoc2l6ZSA9IDEsIGFscGhhID0gMC43KSArDQogIGxhYnModGFnID0gIkUiLCB4ID0gIkxlbmd0aCIsIHkgPSAiV2lkdGgiKQ0KDQojIFZpc3VhbGl6ZSBRIHZhcmlhdGlvbiBhdCBzcGVjaW1lbiBsZXZlbA0KZ2dfc2l6ZV9RIDwtZ2dwbG90KHRyYWl0c19tZXJnZWQsIGFlcyh4PUxlbmd0aF90b193aWR0aF9yYXRpb19tZWFuLCBjb2xvcj1TcGVjaWVzX0lEKSkgKw0KICBnZW9tX2JveHBsb3QoKSArIA0KICBjb29yZF9mbGlwKCkgKyANCiAgbGFicyh0YWcgPSAiRiIsIHggPSAiTGVuZ3RoIHRvIHdpZHRoIHJhdGlvIikNCmBgYA0KDQoNCiMjIyBTY2F0dGVycGxvdHMgZm9yIHRyYWl0cyBpbiBhIHNpbmdsZSBjb21iaW5lZCBwbG90DQpgYGB7cn0NCiNjb25mbGljdGVkOjpjb25mbGljdF9wcmVmZXIoInJlbmFtZSIsICJkcGx5ciIpDQpsaWJyYXJ5KGdncHVicikNCmdnX3BjYXMgPC0gZ2dhcnJhbmdlKGdnX2FzeW1fUEMxdlBDMiwgZ2dfYXN5bV9QQzF2UEMzLCBnZ19zeW1tX1BDMSwgZ2dfZ2xvYl9QQzF2UEMyLCBnZ19zaXplX0x2VywgZ2dfc2l6ZV9RLCBjb21tb24ubGVnZW5kID0gVFJVRSwgbGVnZW5kID0gImJvdHRvbSIsIGFsaWduID0gImh2IikgKyANCiAgdGhlbWUocGxvdC5tYXJnaW4gPSBtYXJnaW4oMC4zLCAwLjYsIDAuMywgMC4zLCAnY20nKSkgDQpgYGANCg0KUHJpbnQgdGhlIHBsb3RzIHRvIGEgZmlsZShzKQ0KYGBge3J9DQojIHRpZmYNCnRpZmYoZmlsZSA9IGhlcmU6OmhlcmUoIjNfcmVzdWx0cyIsICJmaWdfcGNhLnRpZmYiKSwgaGVpZ2h0PSAxMywgd2lkdGg9MTksIHVuaXRzID0gJ2NtJywgcmVzID0gNjAwLCANCiAgICAgY29tcHJlc3Npb24gPSAibHp3IiwgZmFtaWx5ID0gInNhbnMiKQ0KcHJpbnQoZ2dfcGNhcykNCmRldi5vZmYoKQ0KDQojICMgcGRmDQojIHBkZihmaWxlID0gaGVyZTo6aGVyZSgiM19yZXN1bHRzIiwgImZpZ19wY2EucGRmIiksIGhlaWdodD01LjAsIHdpZHRoPTcuNSkNCiMgcHJpbnQoZ2dfcGNhcykNCiMgZGV2Lm9mZigpDQojIA0KIyAjIHBuZw0KIyBwbmcoZmlsZSA9IGhlcmU6OmhlcmUoIjNfcmVzdWx0cyIsICJmaWdfcGNhLnBuZyIpLCBoZWlnaHQ9IDEzLCB3aWR0aD0xOSwgdW5pdHMgPSAnY20nLCByZXMgPSA1MDApDQojIHByaW50KGdnX3BjYXMpDQojIGRldi5vZmYoKQ0KYGBgDQoNCg0KIyMjIFRyYWl0IGRhdGE6IG5vcm1hbGl0eSB0ZXN0IGZvciBlYWNoIGxldmVsIG9mIHRoZSBncm91cGluZyB2YXJpYWJsZQ0KYGBge3J9DQpzaEwgPC0gYXBwbHkodHJhaXRzX21lcmdlZFssNDoxMl0sIDIsICBmdW5jdGlvbih4KSB7UlZBaWRlTWVtb2lyZTo6YnlmLnNoYXBpcm8oeCB+IFNwZWNpZXNfSUQsIGRhdGEgPSB0cmFpdHNfbWVyZ2VkKSR0YWJ9KSAjIGNyZWF0ZXMgbGlzdA0Kc2hEIDwtIGRhdGEudGFibGU6OnJiaW5kbGlzdChzaEwsIGlkY29sID0gVCkgIyBjb252ZXJ0cyBzZXJpZXMgb2YgbGlzdHMgdG8gZGF0YWZyYW1lDQojIGxpbmVzIGp1c3QgYmVsb3cgcmVxdWlyZSBkcGx5cg0Kc2hEJFNwZWNpZXNfSUQgPC0gcmVwKGxldmVscyhmYWN0b3IodHJhaXRzX21lcmdlZCRTcGVjaWVzX0lEKSksIDkpICMgcmUtY3JlYXRlIGNvbHVtbnNvZiBzcGVjaWVzIElEcw0Kc2hEICU+JSByZWxvY2F0ZShTcGVjaWVzX0lELCAuYWZ0ZXIgPSAxKSAlPiUgcmVuYW1lKFRyYWl0ID0gLmlkLCBTaGFwaXJvX1cgPSBXLCBTaGFwaXJvX3AgPSAncC12YWx1ZScpIC0+IHNoRCAjIGNvc21ldGljIGNoYW5nZXMgDQpzaEQkU2hhcGlyb19wIDwtIGFzLm51bWVyaWMoZm9ybWF0KHNoRCRTaGFwaXJvX3AsIHNjaWVudGlmaWM9RkFMU0UpKSAjIHNob3cgcCB2YWx1ZXMgYXMgZGVjaW1hbHMNCnNoRA0KYGBgDQoNCkluIGhvdyBtYW55ICUgb2YgY2FzZXMgKHRyYWl0cyBieSBzcGVjaWVzKSB0aGUgbm9ybWFsaXR5IHdhcyBub3QgbWV0Pw0KYGBge3J9DQpzaGFwaXJvTk4gPC0gc2hEJFNoYXBpcm9fcCA8PSAwLjA1DQpucm93KHNoRFtzaGFwaXJvTk4sXSkgLyBucm93KHNoRCkNCmBgYA0KVGhlcmUgaXMgbm8gdW5pdmVyc2FsIG11bHRpdmFyaWF0ZSBub3JtYWxpdHkgZm9yIGxldmVscyAoZGVmaW5lZCBieSBzcGVjaWVzKSB3aXRoaW4gdHJhaXRzLg0KDQoNCiMjIyBUcmFpdCBkYXRhOiB0ZXN0IG9mIHZhcmlhbmNlIGVxdWFsaXR5IGluIHRyYWl0IHZhcmlhYmxlcyBiZXR3ZWVuIHNwZWNpZXMNCmBgYHtyfQ0KaGVwbG90czo6bGV2ZW5lVGVzdHModHJhaXRzX21lcmdlZFssNDoxMl0sIGZhY3Rvcih0cmFpdHNfbWVyZ2VkJFNwZWNpZXNfSUQpKQ0KYGBgDQpGb3IgZWFjaCBvZiB0aGUgdHJhaXRzLCBjb3ZhcmlhbmNlIGJldHdlZW4gdGhlIGdyb3VwcyBkZWZpbmVkIGJ5IHNwZWNpZXMgYXJlIG5vdCBlcXVhbC4gDQoNCg0KIyMjIFRyYWl0IGRhdGE6IG92ZXJhbGwgZGlzdHJpYnV0aW9ucw0KYGBge3J9DQpwYXIobWZyb3cgPSBjKDMsMykpDQpoaXN0KHRyYWl0c19tZXJnZWQkUEMxX3N5bW1fbWVhbiwgbWFpbiA9IE5VTEwsIHhsYWIgPSAiUEMxIHN5bW1ldHJpYyIpDQpoaXN0KHRyYWl0c19tZXJnZWQkUEMxX2FzeW1fbWVhbiwgbWFpbiA9IE5VTEwsIHhsYWIgPSAiUEMxIGFzeW1tZXRyaWMiLCB5bGFiID0gTlVMTCkgDQpoaXN0KHRyYWl0c19tZXJnZWQkUEMyX2FzeW1fbWVhbiwgbWFpbiA9IE5VTEwsIHhsYWIgPSAiUEMyIGFzeW1tZXRyaWMiLCB5bGFiID0gTlVMTCkNCg0KaGlzdCh0cmFpdHNfbWVyZ2VkJFBDM19hc3ltX21lYW4sIG1haW4gPSBOVUxMLCB4bGFiID0gIlBDMyBhc3ltbWV0cmljIikgDQpoaXN0KHRyYWl0c19tZXJnZWQkUEMxX2dsb2JfbWVhbiwgbWFpbiA9IE5VTEwsIHhsYWIgPSAiUEMxIGdsb2JhbCIsIHlsYWIgPSBOVUxMKSANCmhpc3QodHJhaXRzX21lcmdlZCRQQzJfZ2xvYl9tZWFuLCBtYWluID0gTlVMTCwgeGxhYiA9ICJQQzIgZ2xvYmFsIiwgeWxhYiA9IE5VTEwpDQoNCmhpc3QodHJhaXRzX21lcmdlZCRMZW5ndGhfbWVhbiwgbWFpbiA9IE5VTEwsIHhsYWIgPSAiTGVuZ3RoIikNCmhpc3QodHJhaXRzX21lcmdlZCRXaWR0aF9tZWFuLCBtYWluID0gTlVMTCwgeGxhYiA9ICJXaWR0aCIsIHlsYWIgPSBOVUxMKSANCmhpc3QodHJhaXRzX21lcmdlZCRMZW5ndGhfdG9fd2lkdGhfcmF0aW9fbWVhbiwgbWFpbiA9IE5VTEwsIHhsYWIgPSAiTGVuZ3RoIHRvIHdpZHRoIHJhdGlvIiwgeWxhYiA9IE5VTEwpDQojIGlmIHdvcmsgZnJvbSB0aGUgY29uc29sZSwgcmVzZXQgZ3JhcGhpYyBzZXR0aW5ncyBiYWNrIHdpdGggZGV2Lm9mZigpIA0KYGBgDQoNCg0KIyMjIE11bHRpY29sbGluZWFyaXR5IGNoZWNrOiBvdmVyYWxsIGNvcnJlbGF0aW9ucyBiZXR3ZWVuIHZhcmlhYmxlcw0KDQojIyMjIFdheSAxDQpNdWx0aWNvbGxpbmVhcml0eSBjaGVjazogY29ycnBsb3QgIA0KR2V0IGNvcnJlbGF0aW9uIHZhbHVlcyAoU3BlYXJtYW4gY29lZmZpY2llbnQpDQpgYGB7cn0NCnRyYWl0c19tZXJnZWRfZm9yQ29yciA8LSBkcGx5cjo6c2VsZWN0KHRyYWl0c19tZXJnZWQsLWMoIlNwZWNpZXNfSUQiLCAiSW1hZ2VfSUQiLCAiU3BlY2ltZW5fSUQiLCAiTl9zcG9yZXMiKSkNCiNuYW1lcyh0cmFpdHNfbWVyZ2VkX2ZvckNvcnIpIHRoaXMgaXMgdG8gZ2V0IHRoZSBhY3R1YWwgbmFtZXMgYW5kIHRvIGNvc3R1bWl6ZSB0aGVtIG1hbnVhbGx5IGFzIEkgZG8gYmVsb3cNCm5hbWVzKHRyYWl0c19tZXJnZWRfZm9yQ29ycikgPC0gYygiUEMxIHN5bW1ldHJpYyIsICJQQzEgYXN5bW1ldHJpYyIsICJQQzIgYXN5bW1ldHJpYyIsIA0KICAgICAgICAgICAgICAgICAgICAgICAgICAgICAgICAgICJQQzMgYXN5bW1ldHJpYyIsICJQQzEgZ2xvYmFsIiwgIlBDMiBnbG9iYWwiLA0KICAgICAgICAgICAgICAgICAgICAgICAgICAgICAgICAgICJMZW5ndGgiLCAiV2lkdGgiLCAiTGVuZ3RoIHRvIHdpZHRoIHJhdGlvIikNCk0gPC0gY29yKHRyYWl0c19tZXJnZWRfZm9yQ29yciwgbWV0aG9kPSJzcGVhcm1hbiIpDQpgYGANCg0KQ29tcHV0aW5nIHRoZSBwLXZhbHVlIG9mIGNvcnJlbGF0aW9ucyAgDQpUbyBjb21wdXRlIHRoZSBtYXRyaXggb2YgcC12YWx1ZSwgYSBjdXN0b20gUiBmdW5jdGlvbiBpcyB1c2VkLiBTb3VyY2UgaXMgW2hlcmVdKGh0dHA6Ly93d3cuc3RoZGEuY29tL2VuZ2xpc2gvd2lraS92aXN1YWxpemUtY29ycmVsYXRpb24tbWF0cml4LXVzaW5nLWNvcnJlbG9ncmFtKQ0KYGBge3J9DQojIG1hdCA6IGlzIGEgbWF0cml4IG9mIGRhdGENCiMgLi4uIDogZnVydGhlciBhcmd1bWVudHMgdG8gcGFzcyB0byB0aGUgbmF0aXZlIFIgY29yLnRlc3QgZnVuY3Rpb24NCmNvci5tdGVzdCA8LSBmdW5jdGlvbihtYXQsIC4uLikgew0KICAgIG1hdCA8LSBhcy5tYXRyaXgobWF0KQ0KICAgIG4gPC0gbmNvbChtYXQpDQogICAgcC5tYXQ8LSBtYXRyaXgoTkEsIG4sIG4pDQogICAgZGlhZyhwLm1hdCkgPC0gMA0KICAgIGZvciAoaSBpbiAxOihuIC0gMSkpIHsNCiAgICAgICAgZm9yIChqIGluIChpICsgMSk6bikgew0KICAgICAgICAgICAgdG1wIDwtIGNvci50ZXN0KG1hdFssIGldLCBtYXRbLCBqXSwgLi4uKQ0KICAgICAgICAgICAgcC5tYXRbaSwgal0gPC0gcC5tYXRbaiwgaV0gPC0gdG1wJHAudmFsdWUNCiAgICAgICAgfQ0KICAgIH0NCiAgY29sbmFtZXMocC5tYXQpIDwtIHJvd25hbWVzKHAubWF0KSA8LSBjb2xuYW1lcyhtYXQpDQogIHAubWF0DQp9DQpgYGANCg0KR2V0IHAgKHNpZ25pZmljYW5jZSkgdmFsdWVzIGZvciBTcGVhcm1hbiBjb3JyZWxhdGlvbiBjb2VmZmljaWVudA0KVGhlc2UgY291bGQgbm90IGJlIGVzdGltYXRlZCBmb3IgYWxsIGNyb3NzLWNvbXBhcmlzb25zLCBhcHBhcmVudGx5IGR1ZSB0byBzbWFsbCBkYXRhIHNpemUuDQpgYGB7cn0NCnAubWF0IDwtIGNvci5tdGVzdChNLCBtZXRob2Q9InNwZWFybWFuIiwgZXhhY3Q9RkFMU0UpDQpgYGANCg0KUGxvdCBjb3JyZWxhdGlvbiB2YWx1ZXMgd2l0aCBoaWdobGlnaHRpbmcgb25seSBzaWduaWZpY2FudCAoYXQgcD0wLjA1KSBjb3JyZWxhdGlvbiB2YWx1ZXMNCmBgYHtyfQ0KbGlicmFyeShjb3JycGxvdCkNCmNvbCA8LSBjb2xvclJhbXBQYWxldHRlKGMoIiM0NDc3QUEiLCAiIzc3QUFERCIsICIjRkZGRkZGIiwgIiNFRTk5ODgiLCAiI0JCNDQ0NCIpKQ0KDQoNCnRpZmYoZmlsZSA9IGhlcmU6OmhlcmUoIjNfcmVzdWx0cyIsICJmaWdfY29yci50aWZmIiksIGhlaWdodCA9IDE2LjUsIHdpZHRoID0gMTYuNSwgdW5pdHMgPSAnY20nLCByZXMgPSA2MDAsDQogICAgIGNvbXByZXNzaW9uID0gImx6dyIsIGZhbWlseSA9ICJzYW5zIikNCmNvcnJwbG90KE0sIG1ldGhvZD0iY29sb3IiLCBjb2w9Y29sKDIwMCksICANCiAgICAgICAgIHR5cGU9InVwcGVyIiwgDQogICAgICAgICBhZGRDb2VmLmNvbCA9ICJibGFjayIsICMgQWRkIGNvZWZmaWNpZW50IG9mIGNvcnJlbGF0aW9uDQogICAgICAgICB0bC5jb2w9ImJsYWNrIiwgdGwuc3J0PTQ1LCAjVGV4dCBsYWJlbCBjb2xvciBhbmQgcm90YXRpb24NCiAgICAgICAgICMgQ29tYmluZSB3aXRoIHNpZ25pZmljYW5jZQ0KICAgICAgICAgcC5tYXQgPSBwLm1hdCwgc2lnLmxldmVsID0gMC4wNSwgaW5zaWcgPSAiYmxhbmsiLCANCiAgICAgICAgICMgaGlkZSBjb3JyZWxhdGlvbiBjb2VmZmljaWVudCBvbiB0aGUgcHJpbmNpcGFsIGRpYWdvbmFsDQogICAgICAgICBkaWFnPUZBTFNFKQ0KZGV2Lm9mZigpDQoNCg0KIyAjIHBkZg0KIyBwZGYoZmlsZSA9IGhlcmU6OmhlcmUoIjNfcmVzdWx0cyIsICJmaWdfY29yci5wZGYiKSwgaGVpZ2h0PTYuNSwgd2lkdGg9Ni41KQ0KIyBjb3JycGxvdChNLCBtZXRob2Q9ImNvbG9yIiwgY29sPWNvbCgyMDApLCAgDQojICAgICAgICAgIHR5cGU9InVwcGVyIiwgDQojICAgICAgICAgIGFkZENvZWYuY29sID0gImJsYWNrIiwgIyBBZGQgY29lZmZpY2llbnQgb2YgY29ycmVsYXRpb24NCiMgICAgICAgICAgdGwuY29sPSJibGFjayIsIHRsLnNydD00NSwgI1RleHQgbGFiZWwgY29sb3IgYW5kIHJvdGF0aW9uDQojICAgICAgICAgICMgQ29tYmluZSB3aXRoIHNpZ25pZmljYW5jZQ0KIyAgICAgICAgICBwLm1hdCA9IHAubWF0LCBzaWcubGV2ZWwgPSAwLjA1LCBpbnNpZyA9ICJibGFuayIsIA0KIyAgICAgICAgICAjIGhpZGUgY29ycmVsYXRpb24gY29lZmZpY2llbnQgb24gdGhlIHByaW5jaXBhbCBkaWFnb25hbA0KIyAgICAgICAgICBkaWFnPUZBTFNFKQ0KIyBkZXYub2ZmKCkNCiMgDQojIA0KIyAjIHBuZw0KIyBwbmcoZmlsZSA9IGhlcmU6OmhlcmUoIjNfcmVzdWx0cyIsICJmaWdfY29yci5wbmciKSwgaGVpZ2h0ID0gMTYuNSwgd2lkdGggPSAxNi41LCB1bml0cyA9ICdjbScsIHJlcyA9IDUwMCkNCiMgY29ycnBsb3QoTSwgbWV0aG9kPSJjb2xvciIsIGNvbD1jb2woMjAwKSwgIA0KIyAgICAgICAgICB0eXBlPSJ1cHBlciIsIA0KIyAgICAgICAgICBhZGRDb2VmLmNvbCA9ICJibGFjayIsICMgQWRkIGNvZWZmaWNpZW50IG9mIGNvcnJlbGF0aW9uDQojICAgICAgICAgIHRsLmNvbD0iYmxhY2siLCB0bC5zcnQ9NDUsICNUZXh0IGxhYmVsIGNvbG9yIGFuZCByb3RhdGlvbg0KIyAgICAgICAgICAjIENvbWJpbmUgd2l0aCBzaWduaWZpY2FuY2UNCiMgICAgICAgICAgcC5tYXQgPSBwLm1hdCwgc2lnLmxldmVsID0gMC4wNSwgaW5zaWcgPSAiYmxhbmsiLCANCiMgICAgICAgICAgIyBoaWRlIGNvcnJlbGF0aW9uIGNvZWZmaWNpZW50IG9uIHRoZSBwcmluY2lwYWwgZGlhZ29uYWwNCiMgICAgICAgICAgZGlhZz1GQUxTRSkNCiMgZGV2Lm9mZigpDQpgYGANCg0KIyMjIyBXYXkgMg0KTXVsdGljb2xsaW5lYXJpdHkgY2hlY2s6IG9yaWdpbmFsIGRhdGE6IHBzeWNoDQpgYGB7cn0NCnBzeWNoOjpwYWlycy5wYW5lbHModHJhaXRzX21lcmdlZFssNDoxMl0sIGdhcCA9IDAsIGJnID0gYygxOjEwKVtmYWN0b3IodHJhaXRzX21lcmdlZCRTcGVjaWVzX0lEKV0sIHBjaCA9IDIxKQ0KYGBgDQoNCk11bHRpY29sbGluZWFyaXR5IGNoZWNrOmxvZy10cmFuc2Zvcm1lZCBkYXRhOiBwc3ljaDogIGRvZXMgbm90IGNoYW5nZSBtdWNoIGFuZCBrZXB0IGp1c3QgZm9yIGRvY3VtZW50YXRpb24NCmBgYHtyfQ0KIyBwc3ljaDo6cGFpcnMucGFuZWxzKGxvZzIodHJhaXRzX21lcmdlZFssNDoxMl0pLCBnYXAgPSAwLCBwY2ggPSAyMSwgYmcgPSBjKDE6MTApW2ZhY3Rvcih0cmFpdHNfbWVyZ2VkJFNwZWNpZXNfSUQpXSkNCmBgYA0KDQojIyMjIFdheSAzDQpNdWx0aWNvbGxpbmVhcml0eSBjaGVjazogb3JpZ2luYWwgZGF0YTogZ2dwbG90IHZlcnNpb24NCmBgYHtyIG1lc3NhZ2U9RkFMU0V9DQojIEdHYWxseTo6Z2dwYWlycyh0cmFpdHNfbWVyZ2VkWyw0OjEyXSkNCmBgYA0KDQoNCg0KIyMgRGlzY3JpbWluYW50IGFuYWx5c2lzDQoNClRoZSBub24tbm9ybWFsIGRpc3RyaWJ1dGlvbiBhbmQgdW5lcXVhbCB2YXJpYW5jZSBhcmUgcHJlc2VudC4gVGhpcyBwcmV2ZW50cyBmcm9tIHVzaW5nIGxpbmVhciBkaXNjcmltaW5hbnQgYW5hbHlzaXMgb3IgcXVhZHJhdGljIGRpc2NyaW1pbmFudCBhbmFseXNpcy4gSSB3aWxsIHVzZSBmbGV4aWJsZSBkaXNjcmltaW5hbnQgYW5hbHlzaXMuDQoNCk15IGNvZGUgaXMgaW5zcGlyZWQgYnkgW3RoaXMgcG9zdF0oaHR0cHM6Ly9yc3R1ZGlvLXB1YnMtc3RhdGljLnMzLmFtYXpvbmF3cy5jb20vMzU4MTdfMjU1MmUwNWYxZDRlNGRiOGJhODdiMzM0MTAxYTQzZGEuaHRtbCkNCg0KDQpVbml2ZXJzYWwgZnVuY3Rpb24gZm9yIG15IGRpc2NyaW1pbmFudCBhbmFseXNpcyBvZiBmdW5nYWwgc3BvcmVzOg0KYGBge3J9DQpteUZEQSA8LSBmdW5jdGlvbih5LCB4LCBteWRhdGEsIHBhcnQsIHJlcCl7DQogICMgc2VlZCBmb3IgcmVwcm9kdWNpYmlsaXR5DQogIHNldC5zZWVkKDEyMzQ1KQ0KIA0KICBzdWNjIDwtIGRpbShyZXApDQogIGZvciAoayBpbiAxOnJlcCkgew0KICAgIA0KICAjIGRlZmluZSB0cmFpbiBhbmQgdGVzdCBkYXRhDQogIHRyYWluaW5nX3NhbXBsZXMgPC0gY2FyZXQ6OmNyZWF0ZURhdGFQYXJ0aXRpb24obXlkYXRhWywgeV0sIHAgPSBwYXJ0LCBsaXN0ID0gRkFMU0UpICANCiAgDQogIHRyYWluX2RhdGEgPC0gbXlkYXRhW3RyYWluaW5nX3NhbXBsZXMsIF0NCiAgdGVzdF9kYXRhIDwtIG15ZGF0YVstdHJhaW5pbmdfc2FtcGxlcywgXQ0KICANCiAgIyBmbGV4aWJsZSBkaXNjcmltaW5hbnQgYW5hbHlzaXMNCiAgbSA8LSBtZGE6OmZkYShwYXN0ZSh5LCAnficsICB4KSwgZGF0YSA9IHRyYWluX2RhdGEpDQogICMgdGFibGUgd2l0aCBwcmVkaWN0aW9ucyBmb3IgdGVzdCBkYXRhDQogIHRhYmxpbiA8LSB0YWJsZShmYWN0b3IodGVzdF9kYXRhWywgeV0pLCBwcmVkaWN0KG0sIHRlc3RfZGF0YSkpDQogIA0KICAjIGNvdW50IGNvcnJyZWN0IHByZWRpY3Rpb25zID0gdmFsdWVzIHRoYXQgYXJlIG5vdCBpbiB0aGUgZGlhZ29uYWwgb2YgInRhYmxpbiINCiAgc3VjY1trXSA8LSBzdW0oZGlhZyh0YWJsaW4pKS9ucm93KHRlc3RfZGF0YSkNCiAgfQ0KICByZXR1cm4gKG1lYW4oc3VjYykpDQp9DQpgYGANCg0KTm93IGFwcGx5IHRoZSBmdW5jdGlvbiB0byBwYXJ0aWN1bGFyIHNwb3JlIHRyYWl0czoNCmBgYHtyfQ0KIyBTeW1tZXRyaWMgc2hhcGUgdmFyaWF0aW9uDQpzdWNjX1MgPC0gbXlGREEoJ1NwZWNpZXNfSUQnLCAnUEMxX3N5bW1fbWVhbicsIG15ZGF0YSA9IHRyYWl0c19tZXJnZWQsIHBhcnQgPSAwLjcsIHJlcCA9IDEwMDApDQoNCiMgQXN5bW1ldHJpYyBzaGFwZSB2YXJpYXRpb24NCnN1Y2NfQSA8LSBteUZEQSgnU3BlY2llc19JRCcsICdQQzFfYXN5bV9tZWFuICsgUEMyX2FzeW1fbWVhbiArIFBDM19hc3ltX21lYW4nLCBteWRhdGEgPSB0cmFpdHNfbWVyZ2VkLCBwYXJ0ID0gMC43LCByZXAgPSAxMDAwKQ0KDQojIEdsb2JhbCBzaGFwZSB2YXJpYXRpb24NCnN1Y2NfRyA8LSBteUZEQSgnU3BlY2llc19JRCcsICdQQzFfZ2xvYl9tZWFuICsgUEMyX2dsb2JfbWVhbicsIG15ZGF0YSA9IHRyYWl0c19tZXJnZWQsIHBhcnQgPSAwLjcsIHJlcCA9IDEwMDApDQoNCiMgTGVuZ3RoICsgd2lkdGgNCnN1Y2NfTFcgPC0gbXlGREEoJ1NwZWNpZXNfSUQnLCAnTGVuZ3RoX21lYW4gKyBXaWR0aF9tZWFuJywgbXlkYXRhID0gdHJhaXRzX21lcmdlZCwgcGFydCA9IDAuNywgcmVwID0gMTAwMCkNCg0KIyAgTGVuZ3RoIHRvIHdpZHRoIHJhdGlvDQpzdWNjX1EgPC0gbXlGREEoJ1NwZWNpZXNfSUQnLCAnTGVuZ3RoX3RvX3dpZHRoX3JhdGlvX21lYW4nLCBteWRhdGEgPSB0cmFpdHNfbWVyZ2VkLCBwYXJ0ID0gMC43LCByZXAgPSAxMDAwKQ0KDQojIFRvdGFsIHZhcmlhdGlvbiBhcyBRICYgc2l6ZQ0Kc3VjY19RTFcgPC0gbXlGREEoJ1NwZWNpZXNfSUQnLCAnTGVuZ3RoX3RvX3dpZHRoX3JhdGlvX21lYW4gKyBMZW5ndGhfbWVhbiArIFdpZHRoX21lYW4nLCBteWRhdGEgPSB0cmFpdHNfbWVyZ2VkLCBwYXJ0ID0gMC43LCByZXAgPSAxMDAwKQ0KDQojIFRvdGFsIHZhcmlhdGlvbiBhcyBnbG9iYWwgc2hhcGUgKyBzaXplDQpzdWNjX0dMVyA8LSBteUZEQSgnU3BlY2llc19JRCcsICdQQzFfZ2xvYl9tZWFuICsgUEMyX2dsb2JfbWVhbiArIExlbmd0aF9tZWFuICsgV2lkdGhfbWVhbicsIG15ZGF0YSA9IHRyYWl0c19tZXJnZWQsIHBhcnQgPSAwLjcsIHJlcCA9IDEwMDApDQoNCiMgVG90YWwgdmFyaWF0aW9uIHNoYXBlIHN5bW1ldHJpYyArIGFzeW1tZXRyaWMgJiBzaXplDQpzdWNjX1NBTFcgPC0gbXlGREEoJ1NwZWNpZXNfSUQnLCAnUEMxX3N5bW1fbWVhbiArIA0KICAgICAgICAgICAgICAgUEMxX2FzeW1fbWVhbiArIFBDMl9hc3ltX21lYW4gKyBQQzNfYXN5bV9tZWFuICsgDQogICAgICAgICAgICAgICBMZW5ndGhfbWVhbiArIFdpZHRoX21lYW4nLCBteWRhdGEgPSB0cmFpdHNfbWVyZ2VkLCBwYXJ0ID0gMC43LCByZXAgPSAxMDAwKQ0KYGBgDQoNCkFsbCBzdWNjZXNzIHJhdGVzIGluIGEgc2luZ2xlIHRhYmxlDQpgYGB7cn0NCnN1Y2NfYWxsIDwtIHQoZGF0YS5mcmFtZShzdWNjX1MsDQogICAgICAgICAgICAgICAgICAgICAgICAgc3VjY19BLA0KICAgICAgICAgICAgICAgICAgICAgICAgIHN1Y2NfRywNCiAgICAgICAgICAgICAgICAgICAgICAgICBzdWNjX0xXLA0KICAgICAgICAgICAgICAgICAgICAgICAgIHN1Y2NfUSwNCiAgICAgICAgICAgICAgICAgICAgICAgICBzdWNjX1FMVywNCiAgICAgICAgICAgICAgICAgICAgICAgICBzdWNjX0dMVywNCiAgICAgICAgICAgICAgICAgICAgICAgICBzdWNjX1NBTFcpKQ0Kc3VjY19hbGwgICAgICAgICAgICAgICAgICAgICAgICAgIA0KYGBgDQoNClJlLWZvcm1hdCBhbmQgcGxvdCB0aGUgaWRlbnRpZmljYXRpb24gc3VjY2VzcyByYXRlcw0KYGBge3J9DQpzdWNjX2FsbF9kZiA8LSBkYXRhLmZyYW1lKHN1Y2NfYWxsKQ0KIyByb3cubmFtZXMoZGF0YS5mcmFtZShzdWNjX2FsbCkpICMgdG8gcmV0cmlldmUgdGhlIG5hbWVzIG9mIHRoZSBwcmVkaWN0b3JzIGFuZCBhZGp1c3QgdGhlbQ0KIyBzdWNjX2FsbF9kZiRQcmVkaWN0b3JzIDwtIHJvdy5uYW1lcyhkYXRhLmZyYW1lKHN1Y2NfYWxsKSkNCnN1Y2NfYWxsX2RmJFByZWRpY3RvcnMgPC0gYygnUycsICdBJywgJ0cnLCAnTFcnLCAnUScsICdRTFcnLCAgJ0dMVycsICdTQUxXJykNCg0KbmFtZXMoc3VjY19hbGxfZGYpWzFdIDwtICdTdWNjZXNzJyANCnN1Y2NfYWxsX2RmWzFdIDwtIHJvdW5kKHN1Y2NfYWxsX2RmWzFdKjEwMCwgMSkgIyBzd2l0Y2ggdG8gcGVyY2VudHMNCg0KIyBtYWtlIHByZWRpY29ycyB0byBiZSBmYWN0b3JzLCBpbnN0ZWFkIG9mIGNoYXJhY3RlcnMuIFRoaXMgd2lsbCBrZWVwIHRoZWlyIG9yaWdpbmFsIG9yZGVyIHdoaWxlIHBsb3R0aW5nLiANCnN1Y2NfYWxsX2RmJFByZWRpY3RvcnMgPC0gZmFjdG9yKHN1Y2NfYWxsX2RmJFByZWRpY3RvcnMsIGxldmVscyA9IHN1Y2NfYWxsX2RmJFByZWRpY3RvcnMpDQpwIDwtIGdncGxvdChzdWNjX2FsbF9kZiwgYWVzKHg9cmVvcmRlcihQcmVkaWN0b3JzLCBTdWNjZXNzKSwgeT1TdWNjZXNzKSkgKw0KICBnZW9tX2JhcihzdGF0PSJpZGVudGl0eSIsIGZpbGw9InN0ZWVsYmx1ZSIpICsNCiAgeGxhYignUHJlZGljdG9ycycpICsNCiAgeWxhYignSWRlbnRpZmljYXRpb24gc3VjY2VzcyByYXRlLCAlJykgKyANCiAgY29vcmRfZmxpcCh5bGltID0gYyg0NSwgNjUpKSArIA0KICBnZW9tX3RleHQoYWVzKGxhYmVsPVN1Y2Nlc3MpLCB2anVzdD0wLjUsIGhqdXN0ID0gMS40LCBzaXplID0gNCwgY29sb3I9IndoaXRlIikNCiAgdGhlbWUoYXhpcy50aXRsZSA9IGVsZW1lbnRfdGV4dChzaXplID0gMTYpLCBheGlzLnRleHQgPSBlbGVtZW50X3RleHQoc2l6ZSA9IDEyKSkNCnANCmBgYA0KDQpTYXZlIGlkIHN1Y2Nlc3MgcmF0ZXMgcGxvdA0KYGBge3J9DQojIHRpZmYNCnRpZmYoZmlsZSA9IGhlcmU6OmhlcmUoIjNfcmVzdWx0cyIsICJmaWdfc3VjY2Vzc18xMDAwcmVwLnRpZmYiKSwgaGVpZ2h0PSA5LCB3aWR0aD0xMiwgdW5pdHMgPSAnY20nLCByZXMgPSA2MDAsDQogICAgIGNvbXByZXNzaW9uID0gImx6dyIsIGZhbWlseSA9ICJzYW5zIikNCnByaW50KHApDQpkZXYub2ZmKCkNCg0KIyBwbmcoZmlsZSA9IGhlcmU6OmhlcmUoIjNfcmVzdWx0cyIsICJiYXJfZGlzLnBuZyIpLCBoZWlnaHQ9IDksIHdpZHRoPTEyLCB1bml0cyA9ICdjbScsIHJlcyA9IDMwMCkNCiMgcHJpbnQocCkNCiMgZGV2Lm9mZigpDQpgYGANCg0KDQoNCiMjIFBoeWxvZ2VuZXRpYyB0cmVlIHRoYXQganVzdGlmaWVzIHRoZSBhc3NpZ25tZW50IG9mIHNwZWNpbWVucyB0byBzcGVjaWVzICANCg0KIyMjIExvYWQgRE5BIHNlcXVlbmNlcyB0byBSIG1lbW9yeQ0KYGBge3IgbWVzc2FnZSA9IEZBTFNFfQ0KbGlicmFyeShCaW9zdHJpbmdzKQ0KY29uZmxpY3RlZDo6Y29uZmxpY3RfcHJlZmVyKCJzdHJzcGxpdCIsICJCaW9zdHJpbmdzIikNCnNlcSA8LSByZWFkRE5BU3RyaW5nU2V0KGhlcmU6OmhlcmUoIjJfMV9kYXRhX2RuYSIsICIyMDIxMDMiLCAiaXRzXzMwc2VxLmZhc3RhIikpDQojIHNlcUByYW5nZXNATkFNRVMgIyBzZXEgbmFtZXMgYXJlIGhlcmUsIHdvcmsgb24gbmFtZXMgbGF0ZXIgaWYgbmVjZXNzYXJ5DQpgYGANCg0KDQojIyMgTXVsdGlwbGUgc2VxdWVuY2UgYWxpZ25tZW50IChNU0EpDQpgYGB7cn0NCmxpYnJhcnkobXNhKQ0KYWxpX1hTdHJpbmcgPC0gbXNhOjptc2Eoc2VxLCB0eXBlPSJkbmEiLCBtZXRob2Q9Ik11c2NsZSIpDQojdGhlIHJ1biB0b29rIDExLjQ1IHNlY29uZHMgZm9yIDQ4IElUUyBzZXF1ZW5jZXMgb24gbXkgRGVsbCBsYXB0b3AgaW4gQXVnIDIwMjANCg0KbGlicmFyeShhcGUpDQojIGNvbnZlcnQgdGhlIGFsaWdubWVudCB0byBETkFiaW4NCmFsaV9iaW4gPC0gYXMuRE5BYmluKGFsaV9YU3RyaW5nKQ0KYGBgDQoNCg0KIyMjIFBsb3QgTVNBDQojIyMjIFVudHJpbW1lZCBNU0ENCmBgYHtyfQ0KcGFyKG1hcj1jKDMsNywzLDMpKQ0KaW1hZ2UoYWxpX2JpbiwgY2V4LmxhYiA9IDAuOCkNCmBgYA0KDQojIyMjIFRyaW1tZWQgTVNBDQpUcmltbSB0aGUgZW5kcyBvZiBhbGlnbWVudCBhbmQgcGxvdCBhZ2Fpbg0KYGBge3J9DQojIHBhY2thZ2UgaXBzIGlzIHJlcXVpcmVkDQojIHRvIG1ha2UgaXBzIHdvcmtpbmcgcHJvcGVybHksIEkgaGFkIHRvIGluc3RhbGwgWE1MIHBhY2thZ2UgZnJvbSBiaW5hcnkgaW4gdGhlIGZvbGxvd2luZyB3YXk6DQojIGluc3RhbGwucGFja2FnZXMoIlhNTCIsIHR5cGUgPSAiYmluYXJ5IikNCmxpYnJhcnkoaXBzKQ0KYWxpX3RyaW1tZWQgPC0gdHJpbUVuZHMoYWxpX2JpbiwgbWluLm4uc2VxID0gMTUpDQpwYXIobWFyPWMoMywxMCwzLDkpKQ0KaW1hZ2UoYWxpX3RyaW1tZWQsIGNleC5sYWIgPSAwLjgpDQpgYGANCg0KIyMjIE1heGltdW0gTGlrZWxpaG9vZCBwaHlsb2dlbmV0aWMgYW5hbHlzaXMNCmBgYHtyfQ0KbGlicmFyeShwaGFuZ29ybikNCmNvbmZsaWN0ZWQ6OmNvbmZsaWN0X3ByZWZlcigicmVuYW1lIiwgIlM0VmVjdG9ycyIpDQptdCA8LSBtb2RlbFRlc3QocGh5RGF0KGFsaV90cmltbWVkKSwgbW9kZWw9ImFsbCIsIGNvbnRyb2wgPSBwbWwuY29udHJvbCh0cmFjZSA9IDApKQ0KIyBjaG9vc2UgYmVzdCBtb2RlbCB0byB5b3VyIHByZWZlcnJlZCBpbmZvcm1hdGlvbiBjcml0ZXJpYQ0KYmVzdE1vZGVsIDwtIG10JE1vZGVsW3doaWNoLm1pbihtdCRBSUMpXQ0KIyAic29tZSBSIG1hZ2ljIiAtIGNpdGF0aW9uIGZyb20gS2xhdXMgU2NobGllcA0KZW52IDwtIGF0dHIobXQsICJlbnYiKQ0KZml0U3RhcnQgPC0gZXZhbChnZXQoYmVzdE1vZGVsLCBlbnYpLCBlbnY9ZW52KQ0KIyBvcHRpbWl6ZSBtb2RlbA0KZml0Lm5uaSA8LSBvcHRpbS5wbWwoZml0U3RhcnQsIHJlYXJyYW5nZW1lbnQ9Ik5OSSIsIGNvbnRyb2wgPSBwbWwuY29udHJvbCh0cmFjZSA9IDApKQ0KIyBib290c3RyYXAgYW5hbHlzaXMNCmJzIDwtIGJvb3RzdHJhcC5wbWwoZml0Lm5uaSwgYnM9MTAwMCwgb3B0Tm5pPVRSVUUsIGNvbnRyb2wgPSBwbWwuY29udHJvbCh0cmFjZSA9IDApKQ0KDQojIHNhdmUgdGhlIHRyZWUgd2l0aCBib290c3RyYXAgc3VwcG9ydHMgYXMgbm9kZSBsYWJlbHMgaW4gcGh5bG8gb2JqZWN0DQp0cmVlLmJzIDwtIHBsb3RCUyhmaXQubm5pJHRyZWUsIGJzLCB0eXBlPSJwaHlsbyIpDQpgYGANCg0KV2hhdCB3YXMgdGhlIGJlc3Qgc3Vic3RpdHV0aW9uIG1vZGVsPw0KYGBge3J9DQpiZXN0TW9kZWwNCmBgYA0KDQpTYXZlIHRoZSB0cmVlIHRvIGZpbGUNCmBgYHtyfQ0KYXBlOjp3cml0ZS50cmVlKHRyZWUuYnMsIGZpbGUgPSBoZXJlOjpoZXJlKCIzX3Jlc3VsdHMiLCAidHJlZV8xMDAwYnMubmV3aWNrIikpDQpgYGANCg0KDQojIyMgQ3VzdG9tIHBsb3R0aW5nIG9mIHRoZSB0cmVlDQpgYGB7cn0NCg0KIyByZXJvb3QgdGhlIHRyZWUNCnRyZWUuYnMgPC0gcm9vdCh0cmVlLmJzLCBjKCJLSExfMTAyNzIiLCAiS0hMXzEwNzgwIiwgIktITF8xMDgxMyIpLCByZXNvbHZlLnJvb3QgPSBUKQ0KDQpsaWJyYXJ5KGdndHJlZSkNCmxpYnJhcnkoZ2dwbG90MikNCg0KIyBhZGQgc3BlY2llcyBuYW1lcyBpZiBub3QgYWxyZWFkeSBkb25lIGluIHRoZSBtb3JwaG9tZXRyaWMgcGFydCBvZiB0aGUgc2NyaXB0DQpzcF9uYW1lcyA8LSByZWFkLmNzdihoZXJlOjpoZXJlKCIzX3Jlc3VsdHMiLCAic3BlY2llc19uYW1lcy5jc3YiKSwgIHNlcD0iLCIsIHN0cmluZ3NBc0ZhY3RvcnM9RkFMU0UpDQojIG5vdyBhIGNvbXBsZXggb3BlcmF0b3Igd2lsbCBhZGQgdGhlIHNwZWNpZXMgbmFtZXMgdG8gdGhlIG9iamVjdA0KIyBpdCBhbGlnbnMgdGhlIHNwZWNpZXMgbmFtZXMgdG8gc2VxdWVuY2UgbGFiZWxzIGNvcnJlY3RseSAtIEkndmUgY2hlY2tlZCB0aGlzIG1hbnVhbGx5DQojIHRoZXJlIHdpbGwgYmUgYW4gZXJyb3IgbWVzc2FnZSB0aGF0IHRoZSBudW1iZXIgb2YgcmVwbGFjZWQgZWxlbWVudHMgZG9lcyBub3QgZXF1YWwgdGhlDQojIHRvdGFsIG51bWJlciBvZiBlbGVtZW50cyBpbiB0aGUgbGFiZWxzLCBidXQgaXQgaXMgT0sgYmVjYXVzZSB3ZSBvbmx5IGFzc2lnbiB0aGUgc3BlY2llcyBuYW1lcyB0byB0aGUgDQojIHRlcm1pbmFsIG5vZGUgbGFiZWxzID0gdGlwIGxhYmVscywgYW5kIG5vdCB0byBpbnRlcm5hbCBub2RlIGxhYmVscyAod2hpY2ggYXJlIGJvb3RzdHJhcCBzdXBwb3J0cyBoZXJlKQ0KcDAgPC0gZ2d0cmVlKHRyZWUuYnMpICU8KyUgc3BfbmFtZXMNCmBgYA0KDQpOb3cgc2V0dGluZyBhbGwgYWVzdGV0aWNzIA0KYGBge3J9DQpwMSA8LSBwMCArIA0KICBnZW9tX3RyZWUoc2l6ZT0wLjEpICsNCiAgZ2VvbV90aXBsYWIoYWVzKGNvbG9yID0gU3BlY2llc19JRCksIGtleV9nbHlwaCA9IGRyYXdfa2V5X3BvaW50LCBzaXplPTIsIGFsaWduPUYsIGhqdXN0PS0wLjAxKSArIA0KICBnZW9tX3RleHQyKGFlcyhzdWJzZXQgPSAhaXNUaXAsIGxhYmVsID0gcm91bmQoYXMubnVtZXJpYyhsYWJlbCksIGRpZ2l0cyA9IDApKSwgc2l6ZSA9IDIsIHZqdXN0ID0gLTAuNywgaGp1c3QgPSAxLjIpICsgDQogIGdlb21fdHJlZXNjYWxlKGxpbmVzaXplID0gMC41LCBmb250c2l6ZSA9IDIsIHggPSAwLjg1LCB5ID0gMTYuNSkgKyANCiAgZ2VvbV90ZXh0KGxhYmVsPSJzdWJzdGl0dXRpb25zIHBlciBzaXRlIiwgeD0xLCB5PTE2LjAsIA0KICAgICAgICAgICAgc2l6ZSA9IDYgLyAucHQsIGZvbnRmYWNlID0gJ3BsYWluJywgZmFtaWx5ID0gJ3NhbnMnLCBzdGF0ID0gInVuaXF1ZSIpICsgDQogICMgdGhpcyAnc3RhdCA9ICd1bmlxdWUnJyBtYWtlcyB0aGUgdGV4dCBmb250IHRoZSBzYW1lIGFzIGlubmVyIGdncGxvdGluZw0KICB4bGltKC0wLjA0LCAxLjIpICsgDQogIHRoZW1lKGxlZ2VuZC5wb3NpdGlvbj1jKDAuOCwgMC44MSksDQogICAgICAgIGxlZ2VuZC5iYWNrZ3JvdW5kID0gZWxlbWVudF9yZWN0KCksIA0KICAgICAgICBsZWdlbmQua2V5ID0gZWxlbWVudF9ibGFuaygpLCAjIHJlbW92ZXMgdGhlIGJvcmRlcg0KICAgICAgICBsZWdlbmQua2V5LnNpemUgPSB1bml0KDAuNCwgJ2NtJyksIyBzZXRzIG92ZXJhbGwgYXJlYS9zaXplIG9mIHRoZSBsZWdlbmQgDQogICAgICAgIGxlZ2VuZC50ZXh0ID0gZWxlbWVudF90ZXh0KHNpemUgPSA2KSwgIyB0ZXh0IHNpemUgDQogICAgICAgIHRpdGxlID0gZWxlbWVudF90ZXh0KHNpemUgPSA3KSkgKyANCiAgdGhlbWUocGxvdC5tYXJnaW4gPSB1bml0KGMoMC4xLCAwLCAwLjEsIDAuMiksICJjbSIpKSANCiAgIyArbGFicyhjb2w9IlN1YnVsaWN5c3RpZGl1bSBcbiBzcGVjaWVzIikgIyB3b3VsZCBjaGFuZ2UgbGVnZW5kIHRpdGxlDQpgYGANCg0KUHJpbnQgdGhlIHRyZWUgdG8gYSBmaWxlKHMpDQpgYGB7cn0NCiMgdGlmZiB2aWEgdGlmZigpDQp0aWZmKGZpbGVuYW1lID0gaGVyZTo6aGVyZSgiM19yZXN1bHRzIiwgImZpZ190cmVlXzEwMDBicy50aWZmIiksIGhlaWdodD0gMTIsIHdpZHRoPTksIHVuaXRzID0gJ2NtJywgcmVzID0gNjAwLCANCiAgICAgY29tcHJlc3Npb24gPSAibHp3IiwgZmFtaWx5ID0gInNhbnMiKQ0KcHJpbnQocDEpDQpkZXYub2ZmKCkNCg0KIyB0aWZmIHZpYSBnZ3NhdmUNCiMgZ2dzYXZlKGZpbGVuYW1lID0gaGVyZTo6aGVyZSgiM19yZXN1bHRzIiwgImZpZ190cmVlXzIwQlMudGlmZiIpLCBwbG90ID0gcDEsIGhlaWdodD0gMTIsIHdpZHRoPTksIHVuaXRzID0gJ2NtJywgZHBpID0gNDAwKQ0KDQojICMgcGRmDQojIHBkZihmaWxlID0gaGVyZTo6aGVyZSgiM19yZXN1bHRzIiwgImZpZ190cmVlLnBkZiIpLCBoZWlnaHQ9NS4wLCB3aWR0aD0zLjUpDQojIHByaW50KHAxKQ0KIyBkZXYub2ZmKCkNCiMgDQojICMgcG5nDQojIHBuZyhmaWxlID0gaGVyZTo6aGVyZSgiM19yZXN1bHRzIiwgImZpZ190cmVlLnBuZyIpLCBoZWlnaHQ9IDEyLCB3aWR0aD05LCB1bml0cyA9ICdjbScsIHJlcyA9IDUwMCkNCiMgcHJpbnQocDEpDQojIGRldi5vZmYoKQ0KYGBgDQojIyBSZWZlcmVuY2VzDQoNCkdlbmVyYXRlcyB0aGUgYmlibGlvZ3JhcGh5IG9mIGFsbCB1c2VkIFIgcGFja2FnZXMgYW5kIFIgdmVyc2lvbiwgYW5kIGEgcGFyYWdyYXBoIGZvciB0aGUgTWV0aG9kcyBwYXJ0IG9mIHRoZXNpcyAob3IgcGFwZXIpDQpgYGB7cn0NCiMgcGFja2FnZSAicmVwb3J0IiBoYXMgdG8gYmUgaW5zdGFsbGVkIGh0dHBzOi8vZWFzeXN0YXRzLmdpdGh1Yi5pby9yZXBvcnQvYXJ0aWNsZXMvcmVwb3J0Lmh0bWwNCnJlcG9ydDo6cmVwb3J0KHNlc3Npb25JbmZvKCkpDQpgYGANCiAgDQpFbmQgb2YgdGhlIHNjcmlwdC4NCg==
