# Supplementary figures and images for: Geometric morphometric analysis of spore shapes improves identification of fungi
Source: PLoS One. 2021 Aug 5;16(8):e0250477. doi: 10.1371/journal.pone.0250477 (PMC8341628; doi:10.1371/journal.pone.0250477)

-2S.D.

Mean

+2S.D.

PC1

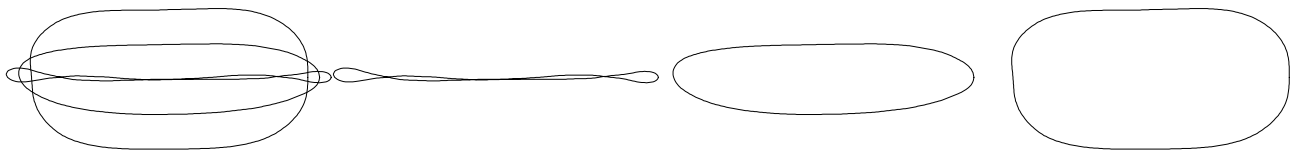

Supplement: S1 Appendix — (ZIP) [file pone.0250477.s002.zip › 3_latest/2_3_data_shape/pca_202102/1_symm/30specimens_clean_symm.pdf]

-2S.D.

Mean

+2S.D.

PC1

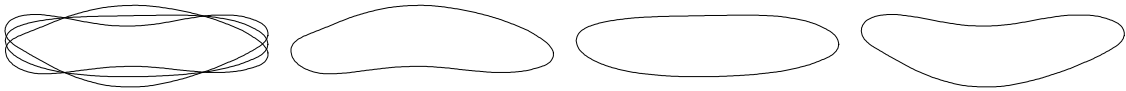

PC2

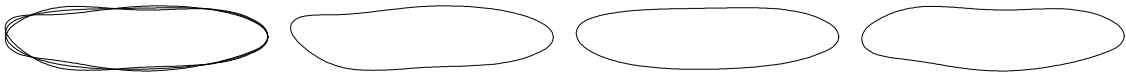

PC3

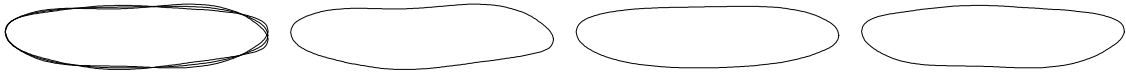

Supplement: S1 Appendix — (ZIP) [file pone.0250477.s002.zip › 3_latest/2_3_data_shape/pca_202102/2_asym/30specimens_clean_asym.pdf]

-2S.D.

Mean

+2S.D.

PC1

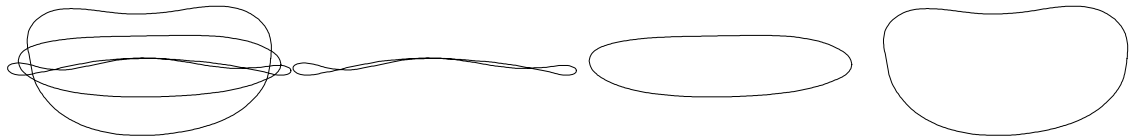

PC2

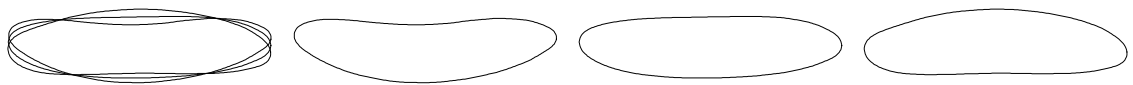

Supplement: S1 Appendix — (ZIP) [file pone.0250477.s002.zip › 3_latest/2_3_data_shape/pca_202102/3_glob/30specimens_clean_glob.pdf]

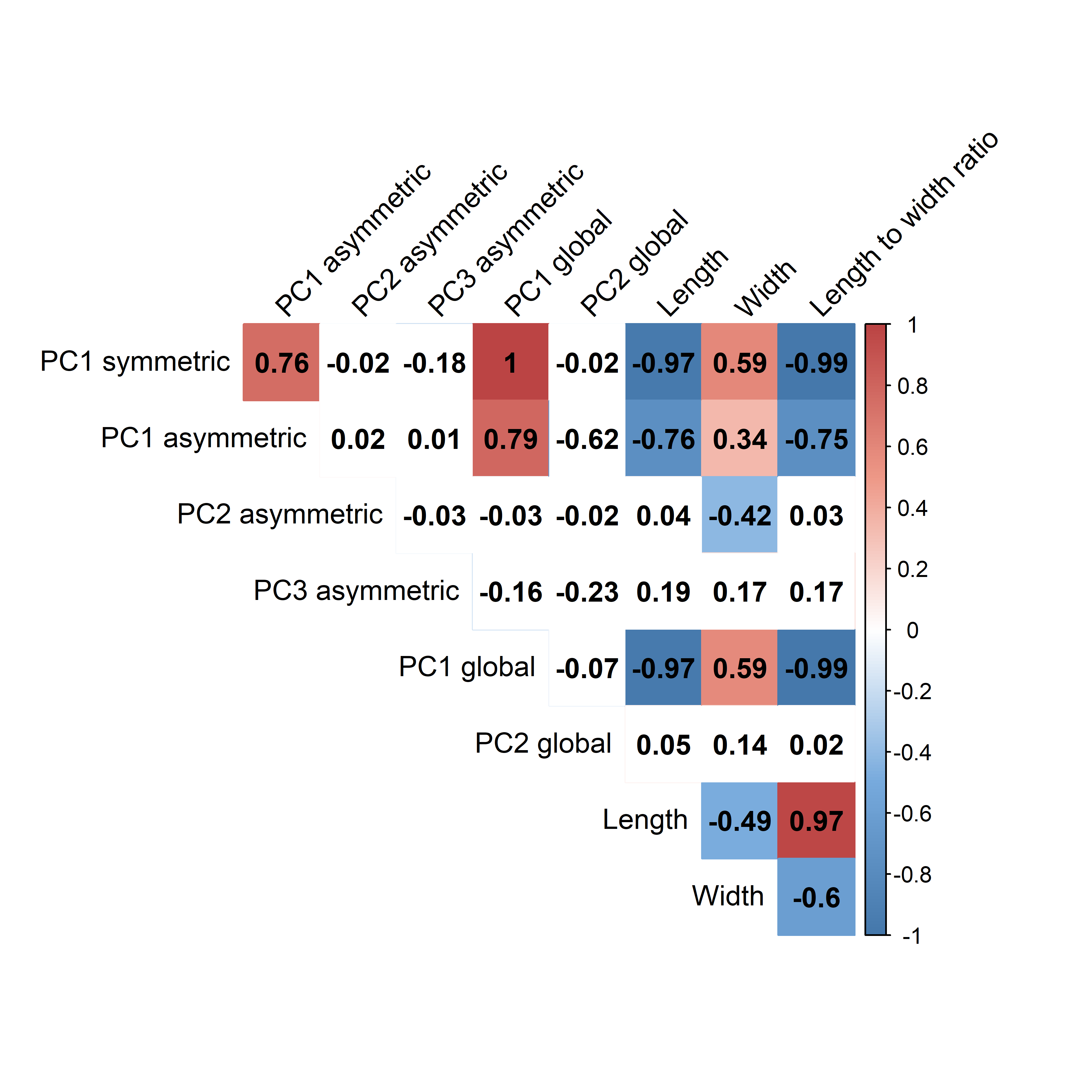

Supplement: S1 Appendix — (ZIP) [file pone.0250477.s002.zip › 3_latest/3_results/fig_corr.tiff]

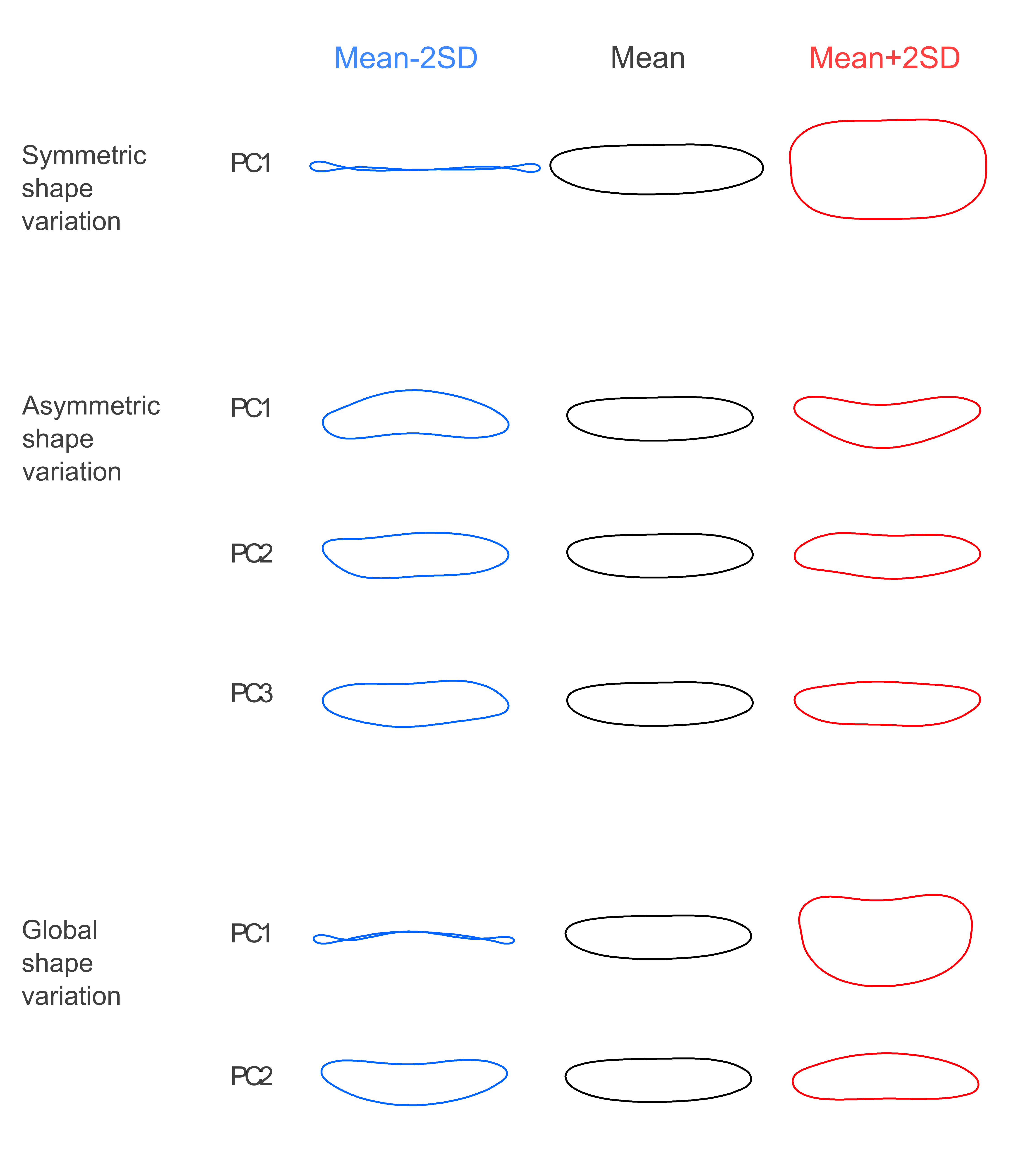

Supplement: S1 Appendix — (ZIP) [file pone.0250477.s002.zip › 3_latest/3_results/fig_outlines_prinprint_inkscape/fig_outlines.png]

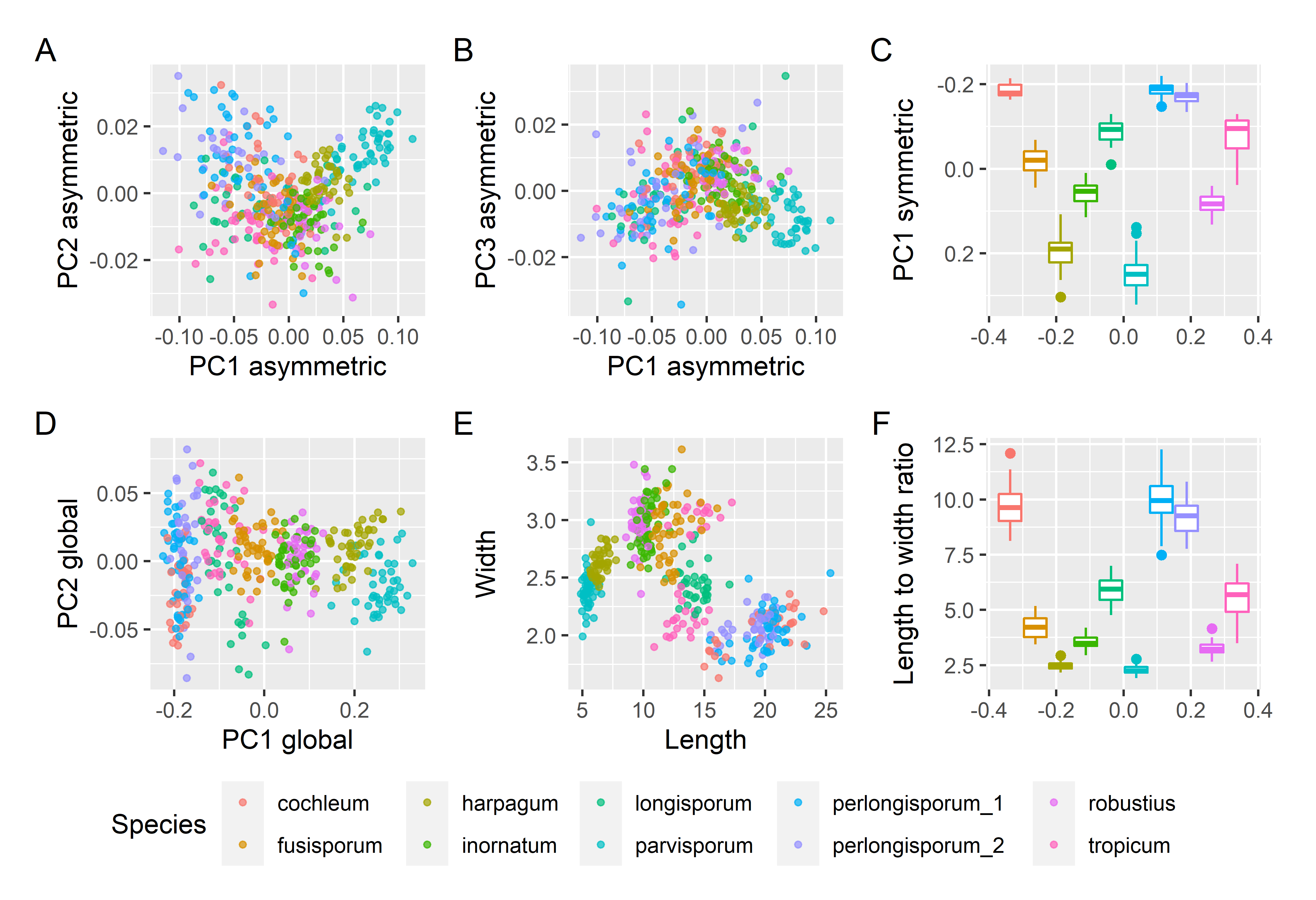

Supplement: S1 Appendix — (ZIP) [file pone.0250477.s002.zip › 3_latest/3_results/fig_pca.tiff]

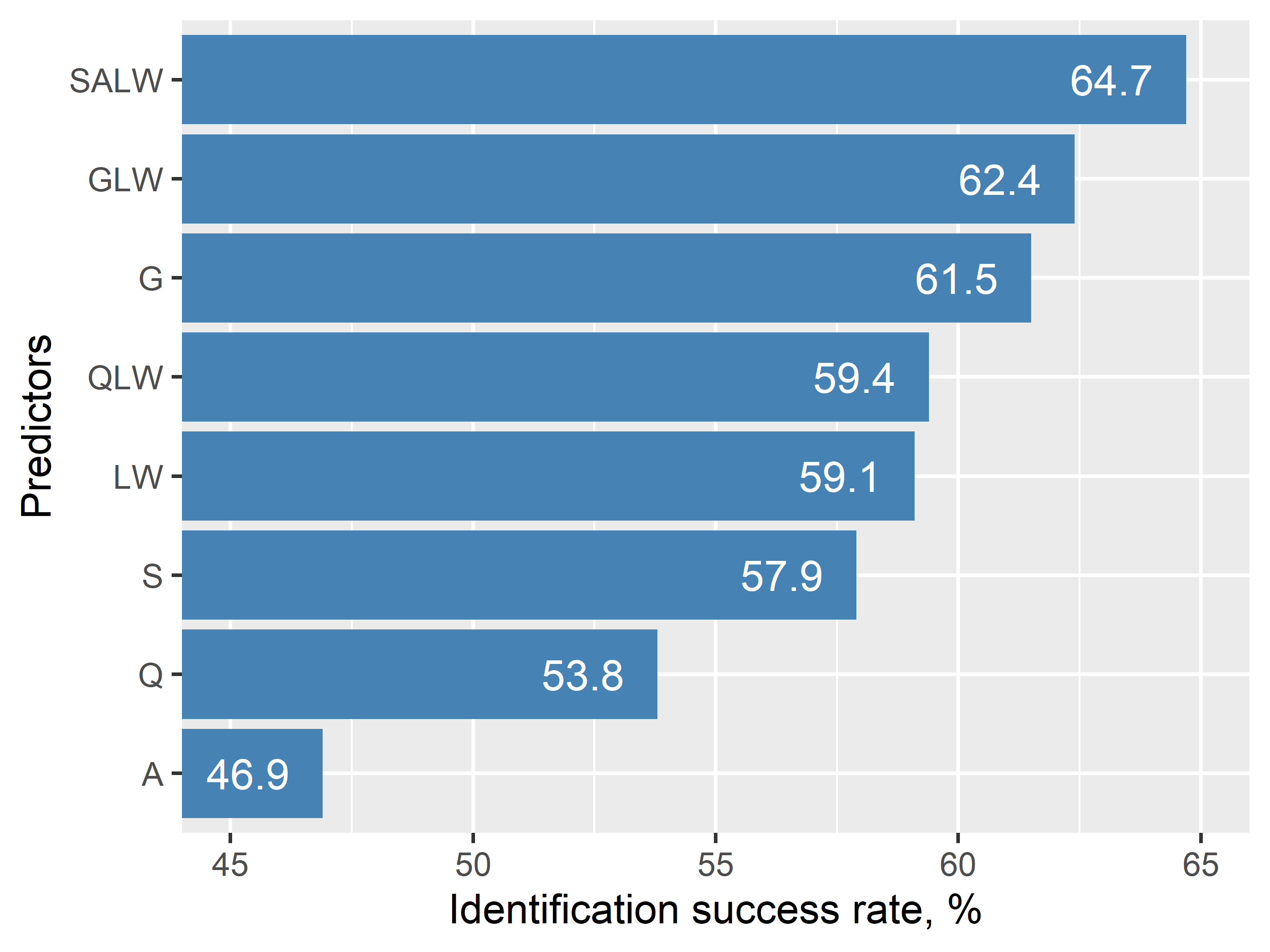

Supplement: S1 Appendix — (ZIP) [file pone.0250477.s002.zip › 3_latest/3_results/fig_success_1000rep.tiff]

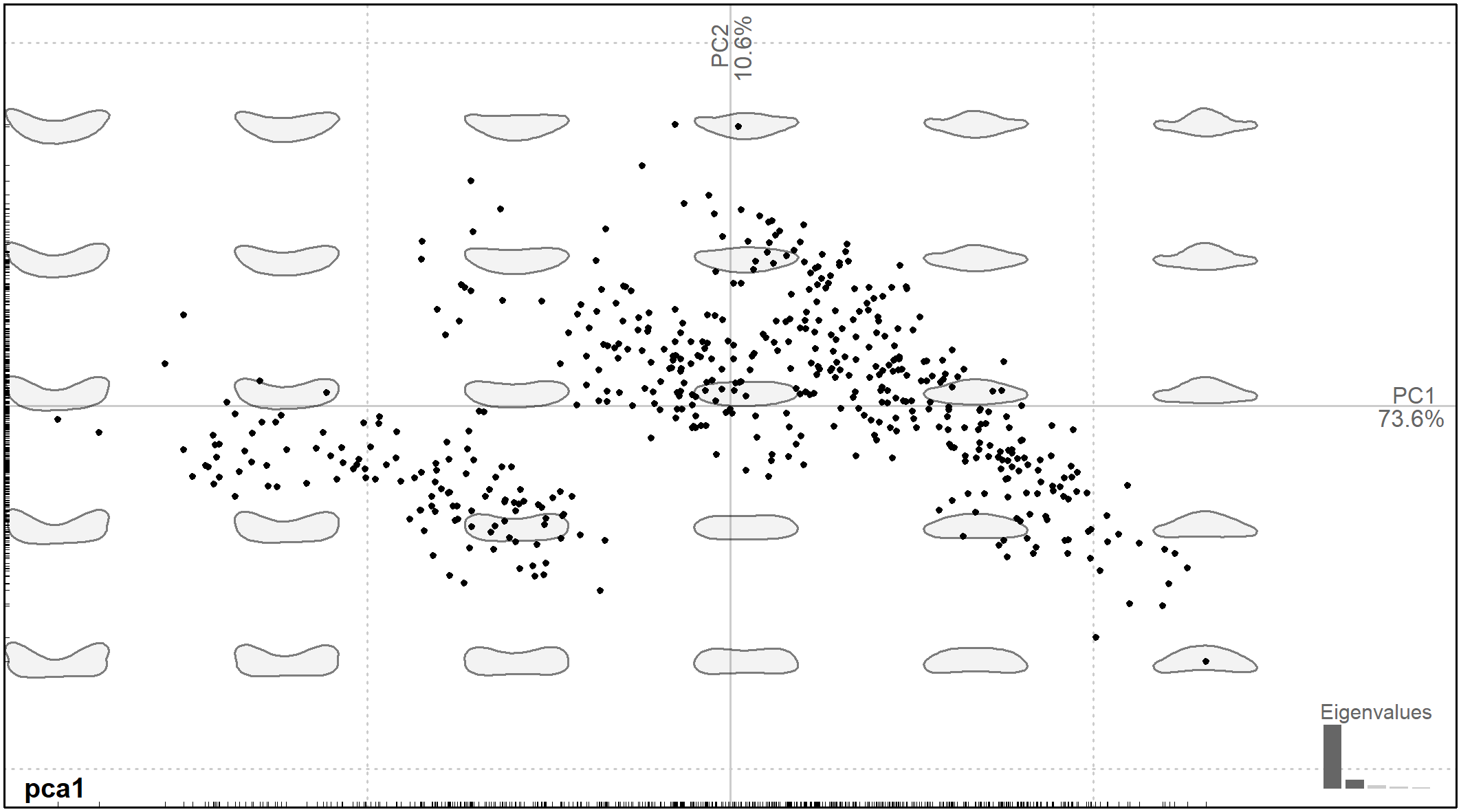

Supplement: S1 Appendix — (ZIP) [file pone.0250477.s002.zip › 3_latest/3_results/fig_suppl2_1pc_no.tiff]

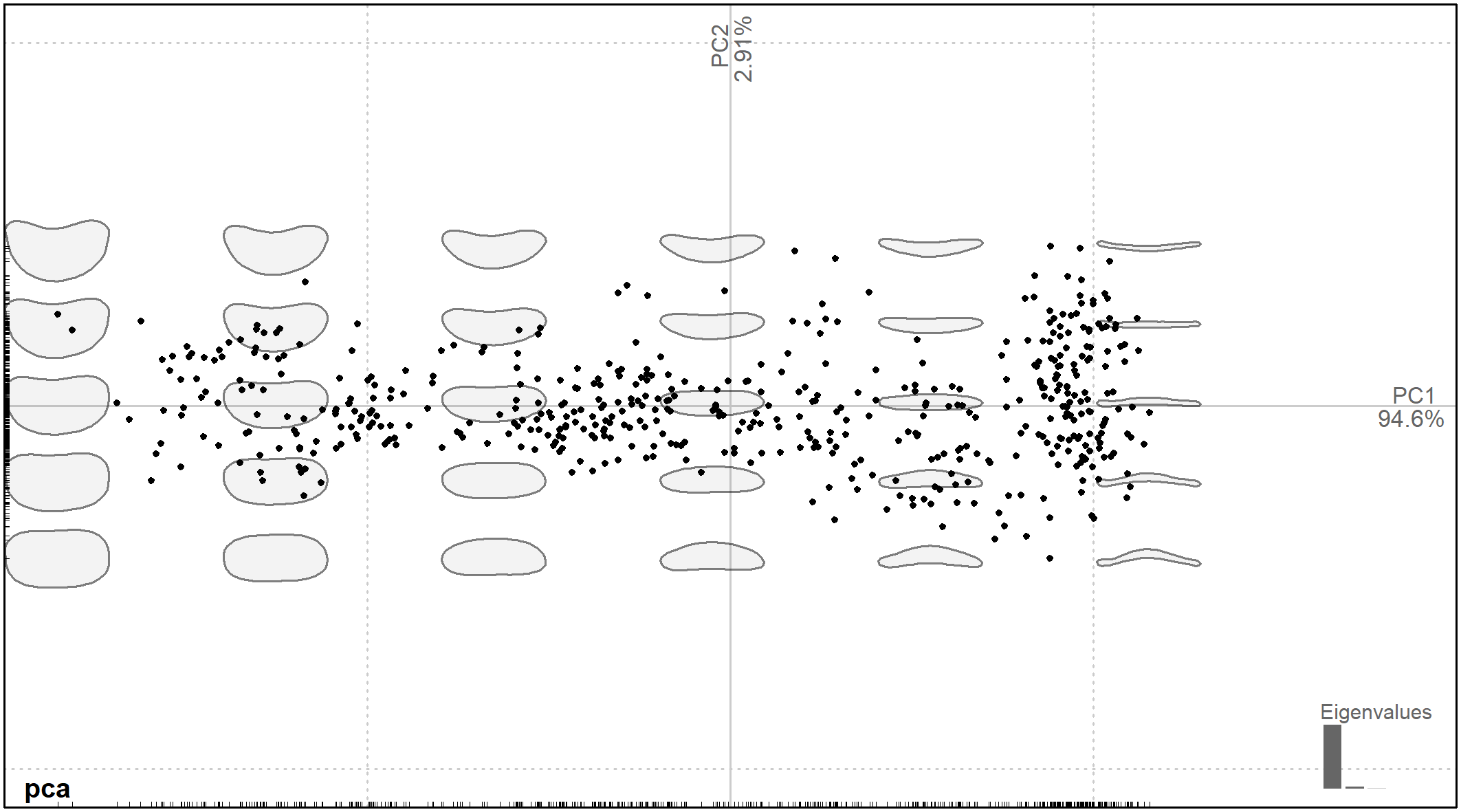

Supplement: S1 Appendix — (ZIP) [file pone.0250477.s002.zip › 3_latest/3_results/fig_suppl2_1pc_yes.tiff]

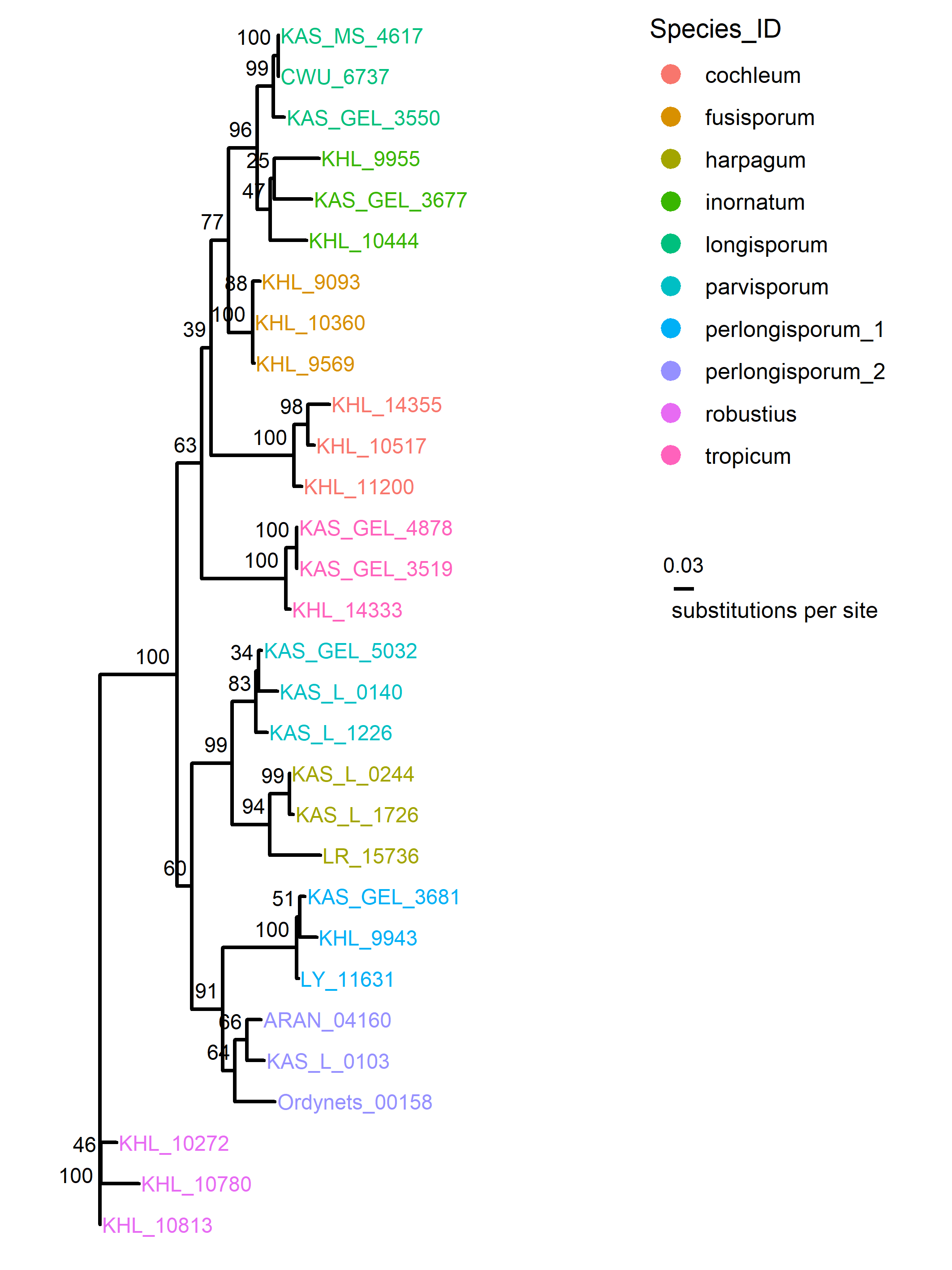

Supplement: S1 Appendix — (ZIP) [file pone.0250477.s002.zip › 3_latest/3_results/fig_tree_1000bs.tiff]
