# Supplementary material for: Geometric morphometric analysis of spore shapes improves identification of fungi
Source: PLoS One. 2021 Aug 5;16(8):e0250477. doi: 10.1371/journal.pone.0250477 (PMC8341628; doi:10.1371/journal.pone.0250477)
Supplement: Attachment — Submitted filename: PONE-D-21-10269_reviewer_rev.pdf [file pone.0250477.s003.pdf]

PLOS ONE

Quantitative analysis of spore shapes improves identification of fungi

--Manuscript Draft--

**Manuscript Number:**	PONE-D-21-10269
**Article Type:**	Research Article
**Full Title:**	Quantitative analysis of spore shapes improves identification of fungi
**Short Title:**	Shape versus size in fungal spores
**Corresponding Author:**	Alexander Ordynets<br>Universitat Kassel FB 10 Mathematik und Naturwissenschaften<br>Kassel, Hesse GERMANY
**Keywords:**	geometric morphometrics; classification; contour; fungi; outline; traditional morphometrics
**Abstract:**	Morphology of organisms is an important source of evidence for biodiversity assessment, taxonomic decisions, and understanding of evolution. Shape information about zoological and botanical objects is often treated quantitatively and in this form improves species identification. In studies of fungi, quantitative shape analysis was almost ignored. The disseminated propagules of fungi, the spores, are crucial for their taxonomy – currently in the form of linear measurements or subjectively defined shape categories. It remains unclear how much quantifying spore shape information can improve species identification. In this study, we tested the hypothesis that shape, as a richer source of information, overperforms size when performing automated identification of fungal species. We used the fungi of the genus <i> Subulicystidium </i> (Agaricomycetes, Basidiomycota) as a study object. We analysed 2D spore shape data via elliptic Fourier and Principal Component analyses. With flexible discriminant analysis, we achieved a slightly higher species identification success rate for shape predictors (61.5%) than for size predictors (59.1%). However, we achieved the highest rate for a combination of both (64.7%). We conclude that quantifying fungal spore shapes is worth the effort. We provide an open access protocol which, we hope, will stimulate a broader use of quantitative shape analysis in fungal taxonomy. We also discuss the challenges of such analyses that are specific to fungal spores.
**Order of Authors:**	Alexander Ordynets
	Sarah Keßler
	Ewald Langer
**Additional Information:**	
**Question**	**Response**
**Financial Disclosure**<br><br>Enter a financial disclosure statement that describes the sources of funding for the work included in this submission. Review the submission guidelines for detailed requirements. View published research articles from *PLOS ONE* for specific examples.<br><br>This statement is required for submission and **will appear in the published article** if the submission is accepted. Please make sure it is accurate.	The authors received no specific funding for this work.

Powered by Editorial Manager® and ProduXion Manager® from Aries Systems Corporation

**Unfunded studies**<br>Enter: *The author(s) received no specific funding for this work.*<br><br>**Funded studies**<br>Enter a statement with the following details:<br>• Initials of the authors who received each award<br>• Grant numbers awarded to each author<br>• The full name of each funder<br>• URL of each funder website<br>• Did the sponsors or funders play any role in the study design, data collection and analysis, decision to publish, or preparation of the manuscript?<br>• **NO** - Include this sentence at the end of your statement: *The funders had no role in study design, data collection and analysis, decision to publish, or preparation of the manuscript.*<br>• **YES** - Specify the role(s) played.<br><br>* typeset	
**Competing Interests**<br><br>Use the instructions below to enter a competing interest statement for this submission. On behalf of all authors, disclose any competing interests that could be perceived to bias this work—acknowledging all financial support and any other relevant financial or non-financial competing interests.<br><br>This statement is required for submission and **will appear in the published article** if the submission is accepted. Please make sure it is accurate and that any funding sources listed in your Funding Information later in the submission form are also declared in your Financial Disclosure statement.<br><br>View published research articles from *PLOS ONE* for specific examples.	The authors have declared that no competing interests exist.

**NO authors have competing interests**<br><br>Enter: *The authors have declared that no competing interests exist.*<br><br>**Authors with competing interests**<br><br>Enter competing interest details beginning with this statement:<br><br>*I have read the journal's policy and the authors of this manuscript have the following competing interests: [insert competing interests here]*<br><br>* typeset	
**Ethics Statement**<br><br>Enter an ethics statement for this submission. This statement is required if the study involved:<br><br>• Human participants<br>• Human specimens or tissue<br>• Vertebrate animals or cephalopods<br>• Vertebrate embryos or tissues<br>• Field research<br><br>Write "N/A" if the submission does not require an ethics statement.<br><br>General guidance is provided below. Consult the submission guidelines for detailed instructions. **Make sure that all information entered here is included in the Methods section of the manuscript.**	N/A

**Format for specific study types**<br><br>**Human Subject Research (involving human participants and/or tissue)**<br>• Give the name of the institutional review board or ethics committee that approved the study<br>• Include the approval number and/or a statement indicating approval of this research<br>• Indicate the form of consent obtained (written/oral) or the reason that consent was not obtained (e.g. the data were analyzed anonymously)<br><br>**Animal Research (involving vertebrate animals, embryos or tissues)**<br>• Provide the name of the Institutional Animal Care and Use Committee (IACUC) or other relevant ethics board that reviewed the study protocol, and indicate whether they approved this research or granted a formal waiver of ethical approval<br>• Include an approval number if one was obtained<br>• If the study involved *non-human primates*, add *additional details* about animal welfare and steps taken to ameliorate suffering<br>• If anesthesia, euthanasia, or any kind of animal sacrifice is part of the study, include briefly which substances and/or methods were applied<br><br>**Field Research**<br><br>Include the following details if this study involves the collection of plant, animal, or other materials from a natural setting:<br>• Field permit number<br>• Name of the institution or relevant body that granted permission	
**Data Availability**<br><br>Authors are required to make all data underlying the findings described fully available, without restriction, and from the time of publication. PLOS allows rare exceptions to address legal and ethical concerns. See the PLOS Data Policy and FAQ for detailed information.	Yes - all data are fully available without restriction

A Data Availability Statement describing where the data can be found is required at submission. Your answers to this question constitute the Data Availability Statement and **will be published in the article**, if accepted.<br><br>**Important:** Stating 'data available on request from the author' is not sufficient. If your data are only available upon request, select 'No' for the first question and explain your exceptional situation in the text box.<br><br>Do the authors confirm that all data underlying the findings described in their manuscript are fully available without restriction?	
**Describe where the data may be found in full sentences. If you are copying our sample text, replace any instances of XXX with the appropriate details.**<br><br>• If the data are **held or will be held in a public repository**, include URLs, accession numbers or DOIs. If this information will only be available after acceptance, indicate this by ticking the box below. For example: *All XXX files are available from the XXX database (accession number(s) XXX, XXX.*).<br>• If the data are all contained **within the manuscript and/or Supporting Information files**, enter the following: *All relevant data are within the manuscript and its Supporting Information files.*<br>• If neither of these applies but you are able to provide **details of access elsewhere**, with or without limitations, please do so. For example:<br><br>*Data cannot be shared publicly because of [XXX]. Data are available from the XXX Institutional Data Access / Ethics Committee (contact via XXX) for researchers who meet the criteria for access to confidential data.*<br><br>*The data underlying the results presented in the study are available from (include the name of the third party*	Metadata on studied voucher specimens is provided in supporting information S1. DNA sequences newly generated for this study are available from GenBank as accessions MW711723-MW711729. Raw and pre-processed images and all information extracted from them are available as a published dataset (Ordynets et al. 2021b, https://doi.org/10.15156/BIO/807451). The R project with the code, input data, and results of analyses is provided in supporting information S2 and as a repository on GitHub (https://github.com/ordynets/size_vs_shape).

and contact information or URL).<br>• This text is appropriate if the data are owned by a third party and authors do not have permission to share the data.<br><br>* typeset	
Additional data availability information:	

Powered by Editorial Manager® and ProduXion Manager® from Aries Systems Corporation

Manuscript Click here to access/download;Manuscript;ms_v20210326.docx

Quantitative analysis of spore shapes improves identification of fungi

Alexander Ordynets[1]*, Sarah Keßler[1], Ewald Langer[1]

[1] Department of Ecology, Faculty of Mathematics and Natural Sciences, University of Kassel, Germany

* Corresponding author

E-Mail: a.ordynets@uni-kassel.de

Abstract

Morphology of organisms is an important source of evidence for biodiversity assessment, taxonomic decisions, and understanding of evolution. Shape information about zoological and botanical objects is often treated quantitatively and in this form improves species identification. In studies of fungi, quantitative shape analysis was almost ignored. The disseminated propagules of fungi, the spores, are crucial for their taxonomy – currently in the form of linear measurements or subjectively defined shape categories. It remains unclear how much quantifying spore shape information can improve species identification. In this study, we tested the hypothesis that shape, as a richer source of information, overperforms size when performing automated identification of fungal species. We used the fungi of the genus *Subulicystidium* (Agaricomycetes, Basidiomycota) as a study object. We analysed 2D spore shape data via elliptic Fourier and Principal Component analyses. With flexible discriminant analysis, we achieved a slightly higher species identification success rate for shape predictors (61.5%) than for size predictors (59.1%). However, we achieved the highest rate for a combination of both (64.7%). We conclude that quantifying fungal spore shapes is worth the effort. We provide an open access protocol which, we hope, will stimulate a broader use of quantitative shape analysis in fungal taxonomy. We also discuss the challenges of such analyses that are specific to fungal spores.

Keywords

geometric morphometrics, classification, contour, fungi, outline, traditional morphometrics

Introduction

In eucaryotic organisms, morphology is an important source of evidence for biodiversity assessment, taxonomic decisions, and understanding of evolutionary and ecological processes. Morphological information is quickly accessible and can be processed at relatively low costs. Therefore it is broadly used by both researchers and citizen scientists. Morphological information, on the one hand, provides a starting point for molecular analyses, and on the other hand, serves as reference data to validate the molecular results [1].

Morphology covers two principal concepts: size and shape. The former is easier to measure and was dominating for more than a century in the quantitative analyses known as traditional morphometrics [2]. Although being important, the size alone is often insufficient for the delimitation of species or populations. For example, within a single taxonomic group of diatom algae (Bacillariophyceae), linear measurements allowed to delimit species in some genera [3] but not in others [4]. The authors of the latter study concluded that involving quantitative shape descriptors, in addition to size, would make delimitation of taxa more efficient. It is a discipline of geometric morphometrics that aims to quantify a shape, geometric information about the object that remains after removing the effects of location, rotation, and scale [5]. Tools of geometric morphometrics allow splitting the shape information into symmetric and asymmetric components and analyse them separately [6]. Besides dealing with richer data of numeric nature, geometric morphometric allows reconstruction of the original look of the object after analyses – a valuable property that traditional morphometrics does not offer [1,2].

Fungi are among the species-richest organism groups on Earth [7]. Generally, for morphology-based fungal taxonomy, the features of the disseminated propagules, the spores, are of the highest priority among phenotypic characters [8]. However, there is a difference in how the size and shape of spores are usually treated. The spore size is routinely used for species delimitations, mostly in the form of quantitative traits such as length and width [9,10]. Spore shape also plays a big role but has been treated differently. The length to width ratio is used frequently as a proxy of the spore shape [9].

However, it is based on linear measurements and has little use for reconstructing the full shape. In most cases, spore shape is treated as a qualitative trait. Spore shape terminology in mycology is a traditional system with dozens of terms that use common geometric shape categories (“globose”, “cylindric”) or similarity to some natural or cultural objects (“ovoid”, “filiform”). The picture becomes further complicated when subcategories are arbitrarily introduced, e.g. via adding prefix “sub-” or epithets “slightly” or “almost”. The subjectivity of this system represents a problem for reproducible research. Furthermore, such an approach makes impossible an assessment of the variation of a trait on an individual, populational, or species level. In their review, [1] concluded that “geometric morphometrics in the study of fungal shapes was far less employed compared to other microscopic organisms”.

To our knowledge, only two studies analysed the shape of fungal spores quantitively. Study [11] found differences in the spore shape between the populations of one species and study [12] – between the populations as well as different species. However, neither of these mycological studies nor most other morphometric studies questioned in a quantitative way how the object’s shape performs compared to size when identifying species. An answer to this question would help to decide how much effort should be invested into digitizing shape information for such small objects as fungal spores. In this study, we test the hypothesis that shape, as a richer source of information, overperforms size when performing automated identification of fungal species. If it is the case, fungal spore shapes are worth the digitizing effort and integration into taxonomic workflows. To test this hypothesis, we are going to answer the following questions:

(i) Is it possible to adequately extract the shape information from images of fungal spores with the software that was developed for macroscopic objects?

(ii) Can the shape differences found in multivariate analyses be reconstituted as the outlines and recognized by a human eye?

(iii) Is it justified to split the spore shape information into symmetric and asymmetric portions or analysis of the overall shape variation would suffice?

As a test system for our study, we will use the genus *Subulicystidium* Parmasto (Hydnodontaceae, Trechisporales, Agaricomycetes, Basidiomycota, Fungi). It is a genus of fungi with 22 known species [13]. All known *Subulicystidium* species have smooth crust-shaped (corticioid) fruiting bodies and occur as saprotrophs on moderately or strongly decayed wood and are common in many forest ecosystems, especially tropical ones. For this genus, we own a large set of spore images after our previous study [14]. In that study, we performed traditional morphometric analysis of the spores while treating spore shapes only qualitatively. An additional advantage of our test system is the availability of DNA sequences for numerous specimens and the availability of the detailed genus-level phylogenetic tree. The DNA-based species assignments will serve as reference information to compare the performance of shape versus size data for automated species identification.

Methods

DNA data

We used a balanced set of 30 herbarium specimens which included ten species of *Subulicystidium* and where each species was equally represented by three specimens. We treated two clades of *S. perlongisporum* described in [15] as two separate species "*S. perlongisporum* 1" and "*S. perlongisporum* 2". For all 30 specimens, we isolated and sequenced the internal transcribed spacer (ITS) of the nuclear ribosomal DNA as described in protocol 1 in [15] or used the sequences we produced earlier [14]. We processed raw sequence data with Geneious version 5.6.7 [16]. We imported the edited sequences to R version 4.0.3 [17] with the package "Biostrings" version 2.58.0 [18] and performed the multiple sequence alignment with the package "msa" version 1.18.0 [19] using MUSCLE algorithm [20] and other settings as default. We then customly trimmed the ends of the alignment with the package "ips" version 0.0.11 [21] to the length when 15 of 30 sequences had non-ambiguous base characters in the first and last position of the alignment. The resulting

alignment had 734 nucleotide positions. We used the alignment viewer available in the package "ape" version 5.4.1 [22] to check visually the sequence alignment. According to the Akaike criterion [23], we identified "TrN+G+I" as the best-fitting nucleotide substitution model. We seacrhed for the best-scoring maximum likelihood phylogenetic tree with the nearest neighbor interchange strategy and performed bootstrap analysis (1000 replicates) with the "phangorn" package version 2.5.5 [24]. We visualized the result with the R package "ggtree" version 2.4.1 [25].

We submitted the newly generated DNA sequences to GenBank [26]. We provide the full list of the used DNA sequences with GenBank accessions and metadata on voucher specimens in supporting information S1.

Morphological data

Spore terminology

To describe the spore morphology, we used the terms as they are found in [27] and [28]. The spores in *Subulicystidium*, as in all Basidiomycota, are produced externally on a sporangium called basidium and remain attached to it till they become mature and ready for discharge (Fig 1). It is the proximal part of the spore that directly contacts the basidium. The distal part of the spore is found on the opposite side of its long axis. The spore has an adaxial side, i.e. turned to the main axis of basidium, and opposite to it an abaxial side. On the proximal part of the spore, there is a projection called hilar appendix that is involved in the spore discharge from a basidium [27]. Observing hilar appendix on the adaxial side of the spore means the spore is seen in the lateral face. Observing hilar appendix directly on the main axis of the spore means the spore is seen in the frontal face. It is correct to compare the shapes of the spores within the same face. In our study, we focus on the spore's lateral face which is more informative in the case of *Subulicystidium*.

Fig 1. Crucial terms for describing a spore of the member of Agaricomycetes

Image acquisition and pre-processing

We acquired and pre-processed images from light microscopy as described in detail in our online protocol [29]. In this paper, we highlight the most essential steps and we illustrate a workflow of image processing in Fig 2. We performed all work on images on a desktop computer with 64-bit Windows 10 operating system (build 19041). We obtained images of spores from squash preparations of fungal herbarium specimens examined at 1000× magnification (Fig 2A). The size of the captured images (JPEG files) was 1024 × 768 or 2048 ×1536 pixels while the resolution was always 96 dpi. We performed bulk image renaming with Bulk Rename Utility version 3.3.1.0 [30] and conversion from JPEG to BMP graphic format with ImageMagick version 7.0.10- [31].

Fig 2. Workflow showing the extraction of the shape and size information from a fungal spore. (A) Image capture and pre-processing, (B) Acquiring linear measurements (C) Acquiring chain codes (D) Transforming chain codes to Normalized Elliptic Fourier Descriptors (E) Checking quality and orientation of the outlines (F) Possibility to open and manage outline descriptors in a text editor. The tools and programs used at each step are named on the left side of each colored panel.

For further processing, we selected those images that contained one or several healthy (not broken, with intact cell wall) mature (not attached to basidia) and well-focused spores [29]. We selected also only the spores that were laying clearly in the lateral face. Furthermore, for meaningful shape analysis, spores had to be alignable, i.e. with hilar appendix stretching out to the same direction if the spore were placed in the same orientation. Some images had to be mirrored to meet this criterion. In some cases, we applied additional manual adjustments, namely painting white lines around some parts of the spore outline, to enhance the clarity of spore outlines and to ensure the

absence of contact with the structures that are other spores or not spores (crystals or hyphae), similar to [10] and [32]. These adjustments did not affect the geometric properties of the spore outlines but enabled a correct outline extraction. Raw and pre-processed images and all information extracted from them are available as a published dataset [33].

Processing shape information

Fungal spores are practically devoid of landmarks, i.e. distinct points that mark angles or cavities and can be unambiguously assigned to an object. Therefore, for fungal spores, methods aiming to reconstruct the complete outline are advantageous. Among such methods, Elliptic Fourier analysis [34] is most commonly employed. It uses an ellipse as a starting shape and searches for the coefficient values that transform the ellipse into the shape that reproduces the original outline of the object. These coefficients are called Elliptic Fourier Descriptors. After normalization, i.e. removing the effects of size, rotation, shift, and starting point of outline recording, they are called Normalized Elliptic Fourier Descriptors (NEFDs) and are used in statistical analysis as variables describing the shape [1].

We performed Elliptic Fourier analysis in the software package SHAPE version 1.3 [35] (http://lbm.ab.a.u-tokyo.ac.jp/~iwata/shape/). We used consequently each of the following programs from this package for different analysis steps: ChainCoder, Ch2Nef, NefViewer, PrinComp, and PrinPrint [29]. We started with grayscaling the BMP images in the program ChainCoder (Fig 2C). Then we converted the images to binary (assigning white pixels to spores and black to background) based on a threshold that was mostly automatically selected by ChainCoder and was only rarely adjusted manually. To remove the shape artifacts, we applied an erosion-dilation filter (that excludes the noisy pixels from the outline) and rarely also a dilation-erosion filter (to fill in artifact cavities). Then a chain code, i.e. a sequence of x and y coordinates describing the outline, was taken for each spore.

We imported the chain codes to Ch2Nef program [35] and checked the orientation of all spores to be the same, i.e. spore hilar appendix placed in the upper left quarter of the image (Fig 2D). When necessary, we applied 180-degree rotation to some of the spores. Ch2Nef then transformed the chain code into the Normalized Elliptic Fourier Descriptors (NEFDs). As a depth of detalisation of the contour information, we opted for 20 levels that are called harmonics in the elliptic Fourier analysis [34]. In general, other studies of organism shapes used from 15 to 30 harmonics [36,37], and using 20 harmonics was sufficient in the classical study from authors of the SHAPE package [6,35]. As each harmonic is described by four coefficients (= Elliptic Fourier Descriptors), there were 80 coefficients that characterised the shape of each spore. As the first three coefficients are always constant, there were 77 coefficients serving as shape variables for the next analysis step. To normalize the Elliptic Fourier Descriptors, we used the approach based on the first harmonic [35]. We checked that NEFDs are alignable with the NefViewer program (Fig 2E).

We combined manually NEFDs for individual specimens into a single .txt file (Fig 2F). To reduce the multidimensionality of NEFDs data, we run on it the Principal Component Analysis (PCA) in PrinComp program [35]. We used the flexibility of PrinComp and performed in total three principal component analyses (PCAs): i) considering only the symmetric shape variation, ii) considering only the asymmetric shape variation, and iii) considering overall shape variation not differentiated into symmetric and asymmetric (hereinafter called global shape variation). As a result of each PCA, we retained those principal components (PC) that explained a substantial amount of variation and were marked by PrinComp as having an eigenvalue >1. Such PCs were called “effective” in PrinComp. We inspected visually the shape variation accounted for by each effective PC with PrinPrint program [29]. The latter reconstructed the mean shape and plus and minus two times the standard deviation shape for each principal component [35].

Processing size information

For exactly those spores for which NEFDs were obtained, we took also the linear measurement: length (the longest dimension) and width (the broadest dimension) according to the standard accepted in mycology [9]. We used Fiji distribution of ImageJ version 1.53c [38] as shown in Fig 2B and described in [29]. Fiji does not differentiate whether the measurement is of a category “length” or “width”. Therefore, we measured constantly firstly length and then width or each spore. Though these values were originally placed in a single column, we split them into length and width columns in R using our protocol [29]. Based on length and width values, we also derived their ratio and used it as an additional variable in statistical analyses.

Comparative analyses

While obtaining the shape descriptors and linear measurements, we had control of the number of spores used per specimen and image. However, if the image contained several spores, we were not able to align the shape descriptors with linear measurements for each particular spore. Even though the input was the same .bmp image, the software for obtaining the shape data (SHAPE) and size data (Fiji) named individual measurements slightly differently. We could overcome it only by producing average-per-image trait values and using them as observations in correlations and discriminant analysis [29]. To produce average-per-image trait values, we used regular expressions in R as well as packages “stringr” version 1.4.0 [39] and “dplyr” version 1.3.0 [40].

For comparison, we assigned the morphometric traits to one of the five categories:

i) principal components of symmetric shape variation
ii) principal components of asymmetric shape variation
iii) principal components of global shape variation
iv) linear measurements (length and width)

v) length to width ratio

We explored how similar were all morphometric traits between themselves by Spearman correlations. To answer which morphometric traits allow more efficient automated identification of fungal species, we applied discriminant analysis. We assessed how accurate the individual traits and their combinations can identify (classify) to species level the observations whose species identity is not known to the statistical model. We found that trait values within species are not necessarily normally distributed and between the species do not have equal variance. For details, see supporting information S2 with the results of Shapiro-Wilk tests (made with R package “RVAideMemoire” version 0.9-79, [41]) and Levene tests (made with R package “heplots” version 1.3-8 [42]). Therefore, we applied an appropriate for such cases flexible discriminant analysis [43] implemented in “mda” R package version 05-2 [44]. We were randomly subsetting the data into the train (70%) and test (30%) portions attempting to balance the presence of data for different species with R package “caret” version 6.0-86 [45]. We repeated to subset the data into train and test portions 1000 times and then calculated the average identification (i.e. correct species assignment) success rate across subsettings using the R code adjusted from [46].

We managed data in R with the packages “here” version 1.0.1 [47], “conflicted” version 1.0.4 [48], “readr” version 1.4.0 [49], “data.table” version 1.13.4 [50], “dplyr” version 1.3.0 [40], and “report” version 0.2.0 [51]. We visualized the results with the packages „ggplot2“ version 3.3.3 [52], “ggpubr” version 0.4.0 [53], function “cor.mtest” [54] and package “corrplot” version 0.84 [55]. We edited Figs 1 and 4 with Inkscape version 0.92.5 [56] and Fig 2 also with Miro online whiteboard (https://miro.com/). We provide the R project with the code, input data and results of analyses in supporting information S2 and as a repository on GitHub (https://github.com/ordynets/size_vs_shape).

Results

Maximum likelihood phylogenetic analysis showed all ten *Subulicystidium* species on the phylogenetic tree as clearly separate clades, mostly with high bootstrap supports (Fig 3).

Fig 3. Phylogenetic relationship of 10 *Subulicystidium* species treated in this study

After all quality filtering steps, we were able to analyse between 10 and 37 spores per specimen which totaled 511 spores from 30 specimens. These spores were captured in 401 images and each image contained one to four spores (supporting information S2). Therefore we considered 401 average-per-image values as observations for analyses.

PCA of symmetric shape variation identified only the first axis as effective (capturing 98,7% of the variation in data) while PCA of asymmetric variation three first axes (capturing 83.03, 6.63 and 4.11% of variation), and PCA of global variation two axes (capturing 94.57 and 2.91% of variation). Together with the spore length, spore width, and length to width ratio, this summed up to nine traits which we compared in terms of efficiency for automated species identification (supporting information S2).

By inspecting visually the shape variation accounted for by each principal component we found that the 1st PC of symmetric variation reflected well the relative thickness of spores (Fig 4). The 1st PC of asymmetric shape variation reflected the direction of single curving of the long axis of the spore. The 2nd and 3rd PCs of asymmetric shape variation reflected the variation in curvature of the proximal and distal end of the spore, respectively. The effects of the 1st and 2nd PCs of the global shape variation corresponded to the 1st PC of symmetric and 1st PC of asymmetric shape variation, respectively.

Fig 4. Ranges of shape variation that separate principal components account for.

When correlating the individual trait variables, we found that 1st PCs of symmetric, asymmetric, and global shape variation correlated strongly positively with each other (Fig 5). All they correlated strongly negatively with the length and moderately positively with the width of the spores. The 2nd PC of asymmetric shape variation correlated moderately negatively with the spore width while the 3rd PC of asymmetric shape variation and 2nd PC of the global shape variation did not correlate significantly with any other trait. Length and width correlated moderately negatively between themselves and each correlated very differently with the length to width ratio, though the correlation was stronger between the length and length to width ratio.

Fig 5. Spearman correlations of spore traits.

Colors show the direction of correlation and highlight only significant correlation values (at level p=0.05)

In the discriminant analysis, the species identification success rate for the individual trait group was highest for the global shape variation (61.5%, Fig 6) while was slightly lower for length and width (59.1%). Symmetric shape variation identified the fungal species better than the length to width ratio (57.9% vs. 53.8%). The asymmetric shape variation had the lowest identification success rate, viz. 46.9%. When combining the traits of different groups, the highest identification success rate was achieved for the combination of symmetric, asymmetric shape variation and linear measurements (64.7%). By using the global shape variation instead of separate symmetric and asymmetric, the success rate was slightly lower (62.4%).

Fig 6. Species identification success rates in discriminant analysis for separate spore traits and their combinations.

Abbreviations on y axis: S = symmetric shape variation (first principal component), A = asymmetric shape variation (three first principal components), G = global shape variation (two first principal components), LW = length + width, Q = length to width ratio, QLW = length to width ratio + length + width, GLW = global shape variation + length + width, SALW = symmetric shape variation + asymmetric shape variation + length + width.

Discussion

Quantitative analysis of shapes helps to better identify and describe the organisms. In studies of fungi, despite their immense morphological diversity, quantitative shape analysis was almost ignored. In this study, we confirmed the hypothesis that shape, as a richer source of information, outperforms size during automated identification of fungal species by their spores. The highest identification success rate was achieved in a discriminant model that combined shape and size descriptors. The symmetric shape variation outperformed the classical length to width ratio. In general, we found that

(i) It is possible to adequately extract the shape information from microscopy images of fungal spores;

(ii) It is possible to recognise by a human eye the spore shape differences reconstituted after the multivariate analyses;

(iii) It is justified to split the spore shape information into symmetric and asymmetric portions for separate analyses.

As quantitative shape analysis has been barely applied in mycology, we first had to ensure that available tools for extracting shape information can be used for our goal. Many of the tools were developed to work on the high-quality images of macroscopic organisms, often photographed as a single object per image in the desired orientation [57–59]. We opted for the program package SHAPE [35] which allows extracting several outlines from the same image, has flexibility for performing PCA and in general, has a convenient interface and detailed manual. Despite this package was originally designed for images of macroscopic objects, we successfully applied it to the outline extraction from the images with a modest resolution made by our microscope camera. The disadvantage of using SHAPE is a need to switch between the separate programs, meaning handling several outcomes and an extra effort in data management. Furthermore, the graphical user interface of SHAPE (or any other GUI program) means investing massive time effort when repeating the analysis and increased risk of producing an error. Therefore, in the future, it is advisory to work in a single environment with all analysis steps written as a code. This will simplify the data management and enhance the reproducibility of the analyses. Functions in R language designed by [60] and further developed by [61] and [62] are promising for this.

With our study design, we observed high correlations between several trait variables. The most important to note is the correlation between the shape descriptors (PC1 of symmetric and asymmetric variation) and size descriptors (especially length). This is due to our choice to represent the size as linear measurements, which are known to be geometrically dependent on a shape [2]. We kept linear measurements as size descriptors to demonstrate their properties and to explore the performance of these classical variables for species identification. We also generated the size variable that is a combination of separate linear measurements and is less independent of shape (square root of the product of length time width, as one possible option according to Claude 2008). This size variable indeed correlated less with shape variables but in discriminant model predicted the species much poorer than the length and width together (38.9% vs 59.1%, see supplementary information S2). In geometric morphometrics, there are also other measures of the size that are

independent of shape and are worth the look by fungal taxonomists [60,63]. Among other trait variables, the correlation between PC1 of global and symmetric shape variation, PC1 of global and asymmetric shape variation, and length and length to width ratio are easily explained by their nested nature. Therefore we did not add these highly correlating variable pairs to the same discriminant models. On the other hand, we struggled to explain the rather high correlation between PC1 of symmetric and asymmetric shape variation. We guess that it is due to a strongly dominating character of PC1 of symmetric shape variation, which was constructed with an account of the first-level elliptic Fourier harmonic (D1).

The identification success rate in our discriminant analyses reached the maximum value of 64.69%. It is hard to compare our results with other studies where authors used different traits and/or different classification methods. Benyon et al. (1999) achieved an identification success rate of 66.2% using seven traits and a higher rate (74.4%) using 20 traits of fungal spores (mostly size descriptors). Authors of [4] obtained similar to our results in discriminant analyses of size and texture traits in diatom algae (55.1% correctly identified items). Authors of [65] reached 100% or nearly such rates for species of diatom algae by shape and texture descriptors. However, they used discriminant analysis as a dimensionality reduction tool and above its result applied other classification techniques. Authors of [66] evaluated their analyses of diatom cell shapes visually, i.e. as strength of separation of observations in the space of discriminant functions. They concluded that taxa with distinct shapes were separated well while the taxa with similar shapes were separated to a less extent. The identification success rates in [32] for statoliths in cubomedusae were comparable, after cross-validation, with ours, but were highly dependent on the number of observations per group. Due to technical reasons explained in Methods section, we performed analyses on average-per-image data. We admit that working with spore-level data would provide a more precise estimate of the correct identification rate.

Future work on quantitative shape analysis of fungal spores should cope with several challenges. Fungal spores may have very different outline properties in lateral versus frontal face. In the current study, we focused on the lateral face which is more peculiar in *Subulicystidium* and allows to capture the curvature along the spores' long axis (while the frontal face masks this feature). However, in other fungal taxa, the lateral and frontal faces are both important [28] and their shapes should be analysed with the same attention. Authors of [32] combined three faces of the objects to achieve a high identification success rate. In fungi, it may be easy to identify the spore as exposed in the lateral face if its hilar appendix is large enough. Unfortunately, this is not the case for all species. Special care should be taken to identify the position of the hilar appendix in the needle-like spores (as in *S. cochleum* and *S. perlongisporum* in our dataset). Applying scanning electron microscopy may help to identify correctly the hilar appendix but also to get a detailed picture of the surface of the fungal spore. While our study focused on the fungi with smooth spores, the latter in many taxa bear additional projections like warts or ridges or distinct spines. These elements would be difficult to capture with the elliptic Fourier descriptors because of the mathematical properties of the method [1]. Therefore, ornamented spores of fungi give a chance to bring another approach, landmark-based methods of geometric morphometric, to mycology. These can be implemented in a two-dimensional and even three-dimensional space. Finally, different properties of the spores in lateral versus frontal face, as well as the availability of the several spore types per species in many fungal lineages, offer a possibility to bring the machine learning techniques to mycology.

We conclude that quantifying fungal spore shapes is worth the effort. We provide an open access protocol to propagate a broader use of quantitative shape analysis in fungal taxonomy and to stimulate the development of more efficient solutions to address the challenges we discussed above.

Data Availability

Metadata on studied voucher specimens is provided in supporting information S1. DNA sequences newly generated for this study are available from GenBank as accessions MW711723-MW711729. Raw and pre-processed images and all information extracted from them are available as a published dataset (Ordynets et al. 2021b, https://doi.org/10.15156/BIO/807451). The R project with the code, input data, and results of analyses is provided in supporting information S2 and as a repository on GitHub (https://github.com/ordynets/size_vs_shape).

Acknowledgements

We acknowledge the curators of fungal collections in ARAN, CWU, GB, LY, and O for providing specimens for our study, and personally Karl-Henrik Larsson, Janett Riebesehl, and Manuel Striegel who collected most of the specimens. David Scherf, Ludmila Lysenko, Ilka Kellner, Robert Liebisch, and Jonathan Denecke performed the DNA lab work and helped with the microscopy of the collections. Anton Savchenko, Oleh Prylutskyi, and Iryna Yatsiuk provided feedback on the earlier version of the protocol for extracting the shape and size information from the spores.

References

1. Fodor E, Hâruța OI. Geometric Morphometrics and the Shape of Microscopic Organisms. Modern Trends in Diatom Identification. 2020; 197–217. doi:10.1007/978-3-030-39212-3_12
2. Zelditch M, Swiderski DL, Sheets HD. Geometric morphometrics for biologists: a primer. Second edition. Amsterdam: Elsevier/Academic Press; 2012.
3. Paull TM, Hamilton PB, Gajewski K, LeBlanc M. Numerical analysis of small Arctic diatoms (Bacillariophyceae) representing the Staurosira and Staurosirella species complexes. Phycologia. 2008;47: 213–224. doi:10.2216/07-17.1
4. Blanco S, Borrego-Ramos M, Olenici A. Disentangling diatom species complexes: does morphometry suffice? PeerJ. 2017;5: e4159. doi:10.7717/peerj.4159

5. Kendall DG. The Diffusion of Shape. Advances in Applied Probability. 1977;9: 428–430. doi:10.2307/1426091

6. Iwata H, Niikura S, Matsuura S, Takano Y, Ukai Y. Evaluation of variation of root shape of Japanese radish (Raphanus sativus L.) based on image analysis using elliptic Fourier descriptors. Euphytica. 1998;102: 143–149. doi:10.1023/A:1018392531226

7. Mora C, Tittensor DP, Adl S, Simpson AGB, Worm B. How Many Species Are There on Earth and in the Ocean? Mace GM, editor. PLoS Biology. 2011;9: e1001127. doi:10.1371/journal.pbio.1001127

8. Sieber TN, Petrini O, Greenacre MJ. Correspondence Analysis as a Tool in Fungal Taxonomy. Systematic and Applied Microbiology. 1998;21: 433–441. doi:10.1016/S0723-2020(98)80053-2

9. Parmasto E, Parmasto I, Möls T. Variation of basidiospores in the hymenomycetes and its significance to their taxonomy. Bresinsky A, Butin H, Schwantes HO, editors. Berlin, Stuttgart: J. Cramer; 1987.

10. Anikster Y, Eilam T, Bushnell WR, Kosman E. Spore dimensions of Puccinia species of cereal hosts as determined by image analysis. Mycologia. 2005;97: 474–484. doi:10.1080/15572536.2006.11832823

11. Infantino A, Zaccardelli M, Costa C, Ozkilinc H, Habibi A, Peever T. A new disease of grasspea (Lathyrus sativus) caused by Ascochyta lentis var. lathyri. Journal of Plant Pathology. 2016;98: 541–548. doi:10.4454/JPP.V98I3.008

12. Fodor E, Hâruţa O, Milenković I, Lyubenova A, Tziros G, Keča N, et al. Geometric morphometry of Phytophthora plurivora sporangia. Annals of Forest Research. 2015;58: 275-294–294. doi:10.15287/afr.2015.411

13. Index Fungorum. Index Fungorum. 2021. Available: http://www.indexfungorum.org

14. Ordynets A, Scherf D, Pansegrau F, Denecke J, Lysenko L, Larsson K-H, et al. Short-spored Subulicystidium (Trechisporales, Basidiomycota): high morphological diversity and only partly clear species boundaries. MycoKeys. 2018;35: 41–99. doi:10.3897/mycokeys.35.25678

15. Ordynets A, Liebisch R, Lysenko L, Scherf D, Volobuev S, Saitta A, et al. Morphologically similar but not closely related: the long-spored species of Subulicystidium (Trechisporales, Basidiomycota). Mycol Progress. 2020;19: 691–703. doi:10.1007/s11557-020-01587-3

16. Kearse M, Moir R, Wilson A, Stones-Havas S, Cheung M, Sturrock S, et al. Geneious Basic: an integrated and extendable desktop software platform for the organization and analysis of sequence data. Bioinformatics. 2012;28: 1647–1649. doi:10.1093/bioinformatics/bts199

17. R Core Team. R: A Language and Environment for Statistical Computing. Vienna, Austria: R Foundation for Statistical Computing; 2020. Available: https://www.r-project.org/

18. Pagès H, Aboyoun P, Gentleman R, DebRoy S. Biostrings: Efficient manipulation of biological strings. 2019. Available: https://bioconductor.org/packages/Biostrings

19. Bodenhofer U, Bonatesta E, Horejš-Kainrath C, Hochreiter S. msa: an R package for multiple sequence alignment. Bioinformatics. 2015;31: 3997–3999. doi:10.1093/bioinformatics/btv494

20. Edgar RC. MUSCLE: multiple sequence alignment with high accuracy and high throughput. Nucleic Acids Research. 2004;32: 1792–1797. doi:10.1093/nar/gkh340

21. Heibl C, Cusimano N, Krah F-S. ips: Interfaces to Phylogenetic Software in R. 2019. Available: https://CRAN.R-project.org/package=ips

22. Paradis E, Schliep K. ape 5.0: an environment for modern phylogenetics and evolutionary analyses in R. Bioinformatics. 2019;35: 526–528. doi:10.1093/bioinformatics/bty633

23. Akaike H. Information theory and an extension of the maximum likelihood principle. In: Petrov B, Csaki B, editors. Second International Symposium on Information Theory. Budapest: Academiai Kiado; 1973. pp. 267–281.

24. Schliep KP. phangorn: phylogenetic analysis in R. Bioinformatics. 2011;27: 592–593. doi:10.1093/bioinformatics/btq706

25. Yu G, Smith DK, Zhu H, Guan Y, Lam TT. ggtree: an R package for visualization and annotation of phylogenetic trees with their covariates and other associated data. Methods in Ecology and Evolution. 2017;8: 28–36. doi:10.1111/2041-210X.12628

26. Benson DA, Cavanaugh M, Clark K, Karsch-Mizrachi I, Lipman DJ, Ostell J, et al. GenBank. Nucleic Acids Res. 2013;41: D36-42. doi:10.1093/nar/gks1195

27. Ingold CT, Hudson HJ. Basidiomycotina. In: Ingold CT, Hudson HJ, editors. The Biology of Fungi. Dordrecht: Springer Netherlands; 1993. pp. 75–108. doi:10.1007/978-94-011-1496-7_5

28. Kõljalg U. Tomentella (Basidiomycota) and related genera in temperate Eurasia. Oslo: Fungiflora; 1996.

29. Ordynets A, Keßler S, Willemsens C. Extracting shape and size information from fungal spores. protocols.io. 2021. doi:dx.doi.org/10.17504/protocols.io.bdeii3ce

30. TGRMN Software Company. Bulk Rename Utility. TGRMN Software Company; 2019. Available: https://www.bulkrenameutility.co.uk/

31. The ImageMagick Development Team. ImageMagick. 2021. Available: https://imagemagick.org

32. Mooney CJ, Kingsford MJ. Statolith Morphometrics Can Discriminate among Taxa of Cubozoan Jellyfishes. PLOS ONE. 2016;11: e0155719. doi:10.1371/journal.pone.0155719

33. Ordynets A, Lysenko L, Kellner I, Scherf D, Liebisch R, Denecke J, et al. Data for the study "Quantitative analysis of spore shapes improves identification of fungi." Universität Gesamthochschule Kassel; 2021. doi:10.15156/BIO/807451

34. Kuhl FP, Giardina CR. Elliptic Fourier features of a closed contour. Computer Graphics and Image Processing. 1982;18: 236–258. doi:10.1016/0146-664X(82)90034-X

35. Iwata H, Ukai Y. SHAPE: A Computer Program Package for Quantitative Evaluation of Biological Shapes Based on Elliptic Fourier Descriptors. Journal of Heredity. 2002;93: 384–385. doi:10.1093/jhered/93.5.384

36. Rohlf FJ, Archie JW. A Comparison of Fourier Methods for the Description of Wing Shape in Mosquitoes (Diptera: Culicidae). Syst Biol. 1984;33: 302–317. doi:10.2307/2413076

37. Yang H-P, Ma C-S, Wen H, Zhan Q-B, Wang X-L. A tool for developing an automatic insect identification system based on wing outlines. Sci Rep. 2015;5: 1–11. doi:10.1038/srep12786

38. Schindelin J, Arganda-Carreras I, Frise E, Kaynig V, Longair M, Pietzsch T, et al. Fiji: an open-source platform for biological-image analysis. Nature Methods. 2012;9: 676–682. doi:10.1038/nmeth.2019

39. Wickham H. stringr: Simple, Consistent Wrappers for Common String Operations. 2019. Available: https://CRAN.R-project.org/package=stringr

40. Wickham H, François R, Henry L, Müller K. dplyr: A Grammar of Data Manipulation. 2021. Available: https://CRAN.R-project.org/package=dplyr

41. Hervé M. RVAideMemoire: Testing and Plotting Procedures for Biostatistics. 2021. Available: https://CRAN.R-project.org/package=RVAideMemoire

42. Fox J, Friendly M, Monette G. heplots: Visualizing Tests in Multivariate Linear Models. 2021. Available: https://CRAN.R-project.org/package=heplots

43. Hastie T, Tibshirani R, Buja A. Flexible Discriminant Analysis by Optimal Scoring. Journal of the American Statistical Association. 1994;89: 1255–1270. doi:10.1080/01621459.1994.10476866

44. Hastie T, Tibshirani R, Leisch F, Hornik K, Ripley BD, Narasimhan B. mda: Mixture and Flexible Discriminant Analysis. 2020. Available: https://CRAN.R-project.org/package=mda

45. Kuhn M, Wing J, Weston S, Williams A, Keefer C, Engelhardt A, et al. caret: Classification and Regression Training. 2020. Available: https://CRAN.R-project.org/package=caret

46. Martos G. Discriminant Analysis in R. 2020 [cited 6 Nov 2020]. Available: https://rstudio-pubs-static.s3.amazonaws.com/35817_2552e05f1d4e4db8ba87b334101a43da.html

47. Müller K. here: A Simpler Way to Find Your Files. 2020. Available: https://CRAN.R-project.org/package=here

48. Wickham H. conflicted: An Alternative Conflict Resolution Strategy. 2019. Available: https://CRAN.R-project.org/package=conflicted

49. Wickham H, Hester J. readr: Read Rectangular Text Data. 2020. Available: https://CRAN.R-project.org/package=readr

50. Dowle M, Srinivasan A. data.table: Extension of `data.frame`. 2020. Available: https://CRAN.R-project.org/package=data.table

51. Makowski D, Ben-Shachar MS, Patil I, Lüdecke D. Automated reporting as a practical tool to improve reproducibility and methodological best practices adoption. CRAN. 2020. Available: https://github.com/easystats/report

52. Wickham H. ggplot2: elegant graphics for data analysis. New York: Springer; 2016. Available: https://ggplot2.tidyverse.org

53. Kassambara A. ggpubr: “ggplot2” Based Publication Ready Plots. 2020. Available: https://CRAN.R-project.org/package=ggpubr

54. Anonym. Visualize correlation matrix using correlogram - Easy Guides - Wiki - STHDA. 2020 [cited 6 Nov 2020]. Available: http://www.sthda.com/english/wiki/visualize-correlation-matrix-using-correlogram

55. Wei T, Simko V. R package "corrplot": Visualization of a Correlation Matrix. 2017. Available: https://github.com/taiyun/corrplot

56. Inkscape Project. Inkscape. 2020. Available: https://inkscape.org

57. Libungan LA, Pálsson S. ShapeR: An R package to study otolith shape variation among fish populations. PLoS ONE. 2015;10. doi:10.1371/journal.pone.0121102

58. Rohlf FJ. The tps series of software. Hystrix It J Mamm. 2015;26: 9–12. doi:10.4404/hystrix-26.1-11264

59. Chuanromanee TS, Cohen JI, Ryan GL. Morphological Analysis of Size and Shape (MASS): An integrative software program for morphometric analyses of leaves. Applications in Plant Sciences. 2019;7: e11288. doi:10.1002/aps3.11288

60. Claude J. Morphometrics with R. New York, NY: Springer New York; 2008. doi:10.1007/978-0-387-77789-4

61. Bonhomme V, Picq S, Gaucherel C, Claude J. Momocs: outline analysis using R. Journal of Statistical Software. 2014;56. doi:10.18637/jss.v056.i13

62. Stela B, Monleón Getino T. Facilitating the automatic characterisation, classification and description of biological images with the VisionBioShape package for R. Open Access Library Journal. 2016;3. doi:10.4236/oalib.1103108

63. Rohlf FJ. Morphometrics. Annual Review of Ecology and Systematics. 1990;21: 299–316.

64. Benyon FHL, Jones AS, Tovey ER, Stone G. Differentiation of allergenic fungal spores by image analysis, with application to aerobiological counts. Aerobiologia. 1999;15: 211–223. doi:10.1023/A:1007501401024

65. Sánchez C, Cristóbal G, Bueno G. Diatom identification including life cycle stages through morphological and texture descriptors. PeerJ. 2019;7. doi:10.7717/peerj.6770

66. Wishkerman A, Hamilton PB. Shape outline extraction software (DiaOutline) for elliptic Fourier analysis application in morphometric studies. Applications in Plant Sciences. 2018;6. doi:10.1002/aps3.1204

Supporting information

S1 Table. GenBank accessions of used DNA sequences and metadata on voucher specimens

S2 Appendix. R project with the code, input data, and results of analyses.

Click here to access/download;Figure;Fig1.eps

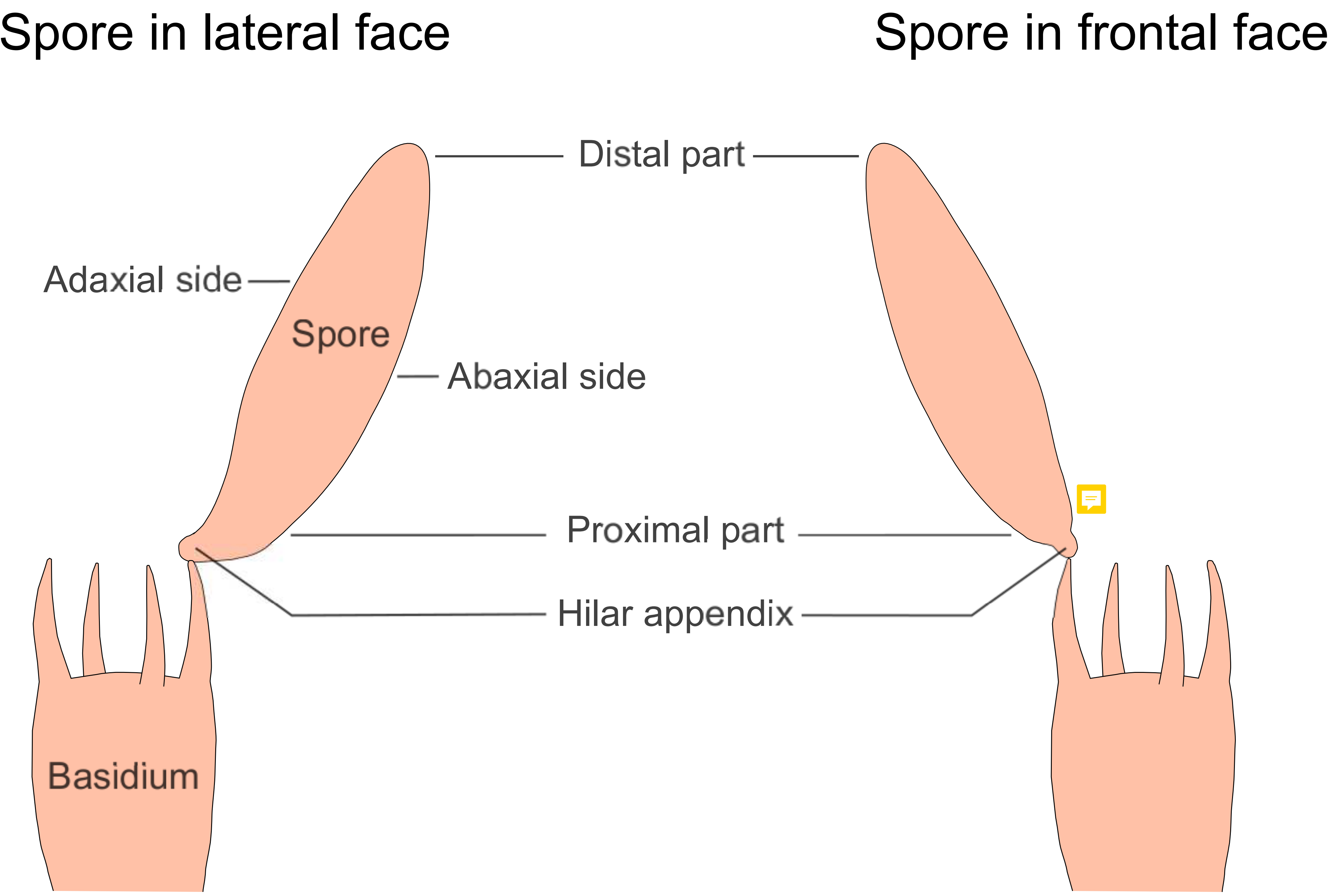

Fig 2

Click here to access/download;Figure;Fig2.tiff

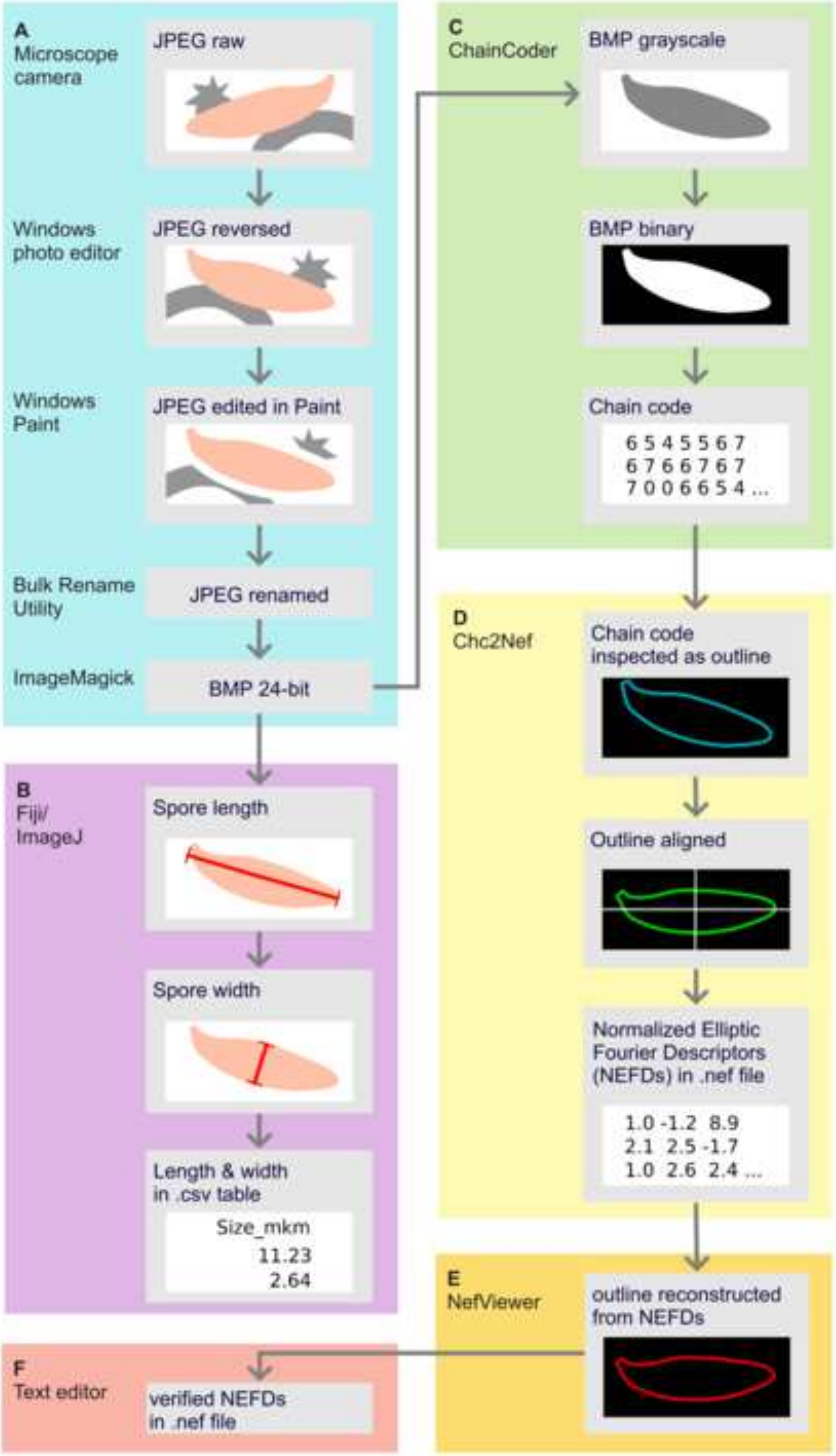

Fig 3

Click here to access/download;Figure;Fig3.tiff

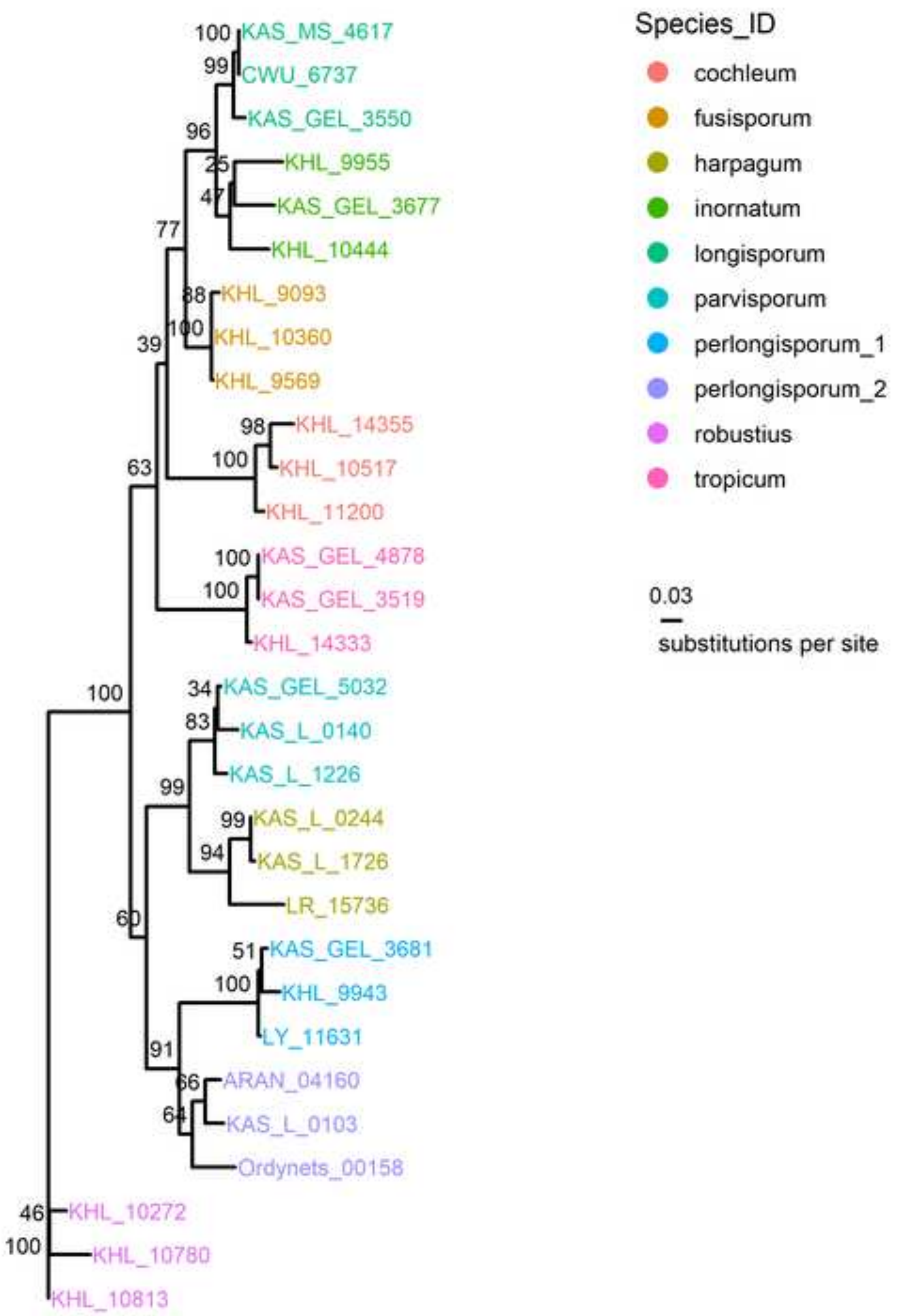

Fig 4

Click here to access/download;Figure;Fig4.eps

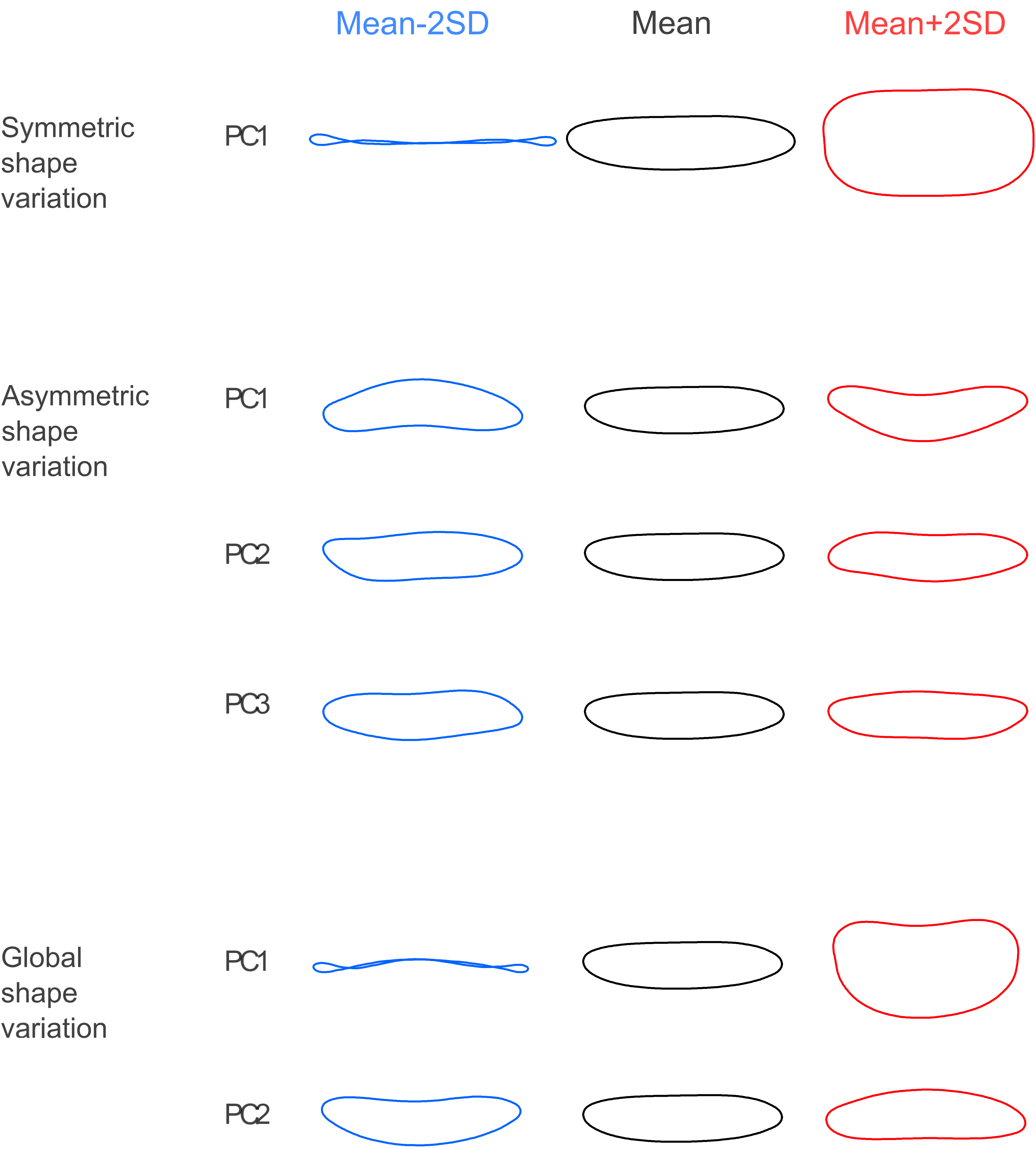

Fig 5

Click here to access/download;Figure;Fig5.tiff

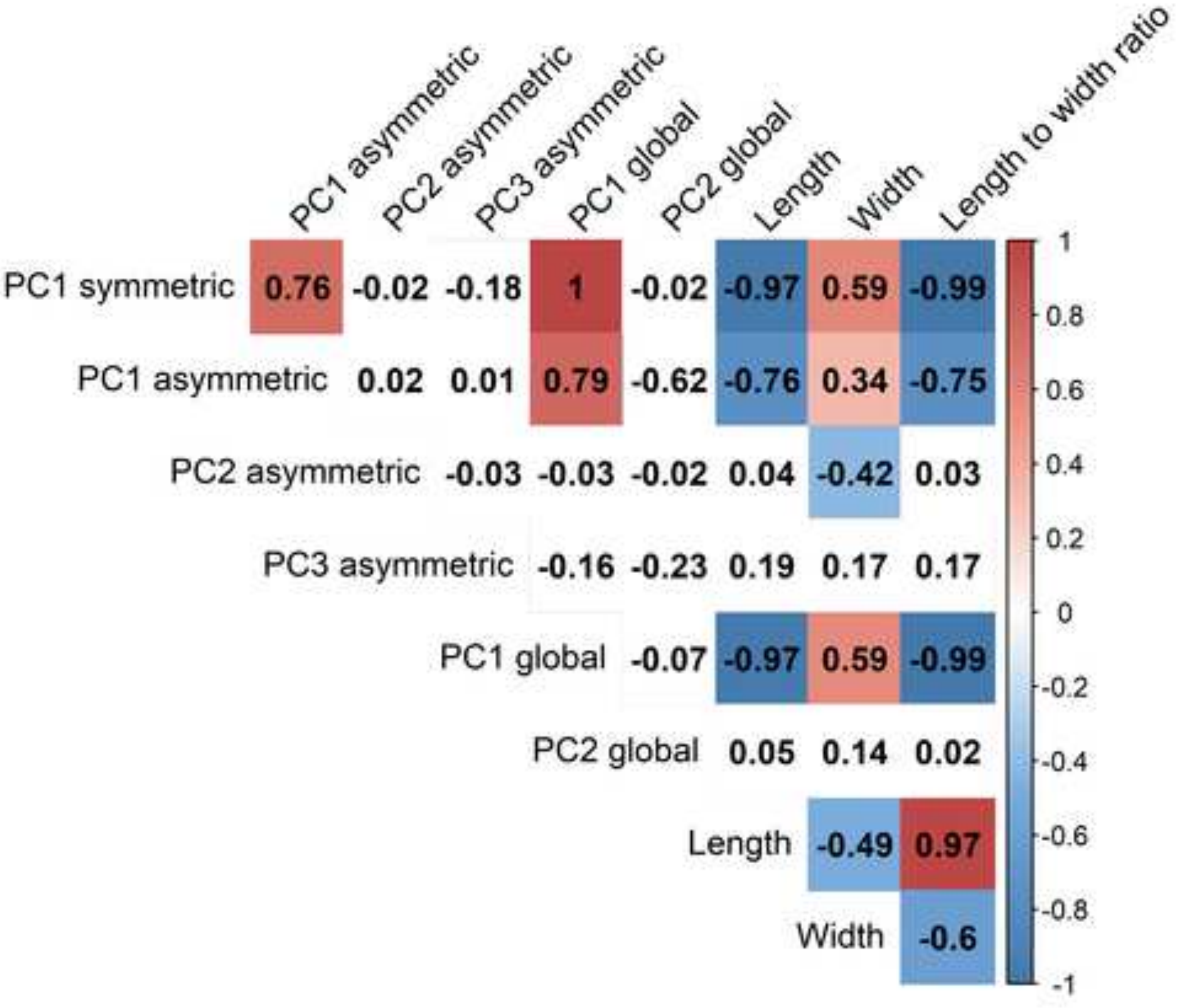

Fig 6

Click here to access/download;Figure;Fig6.tiff

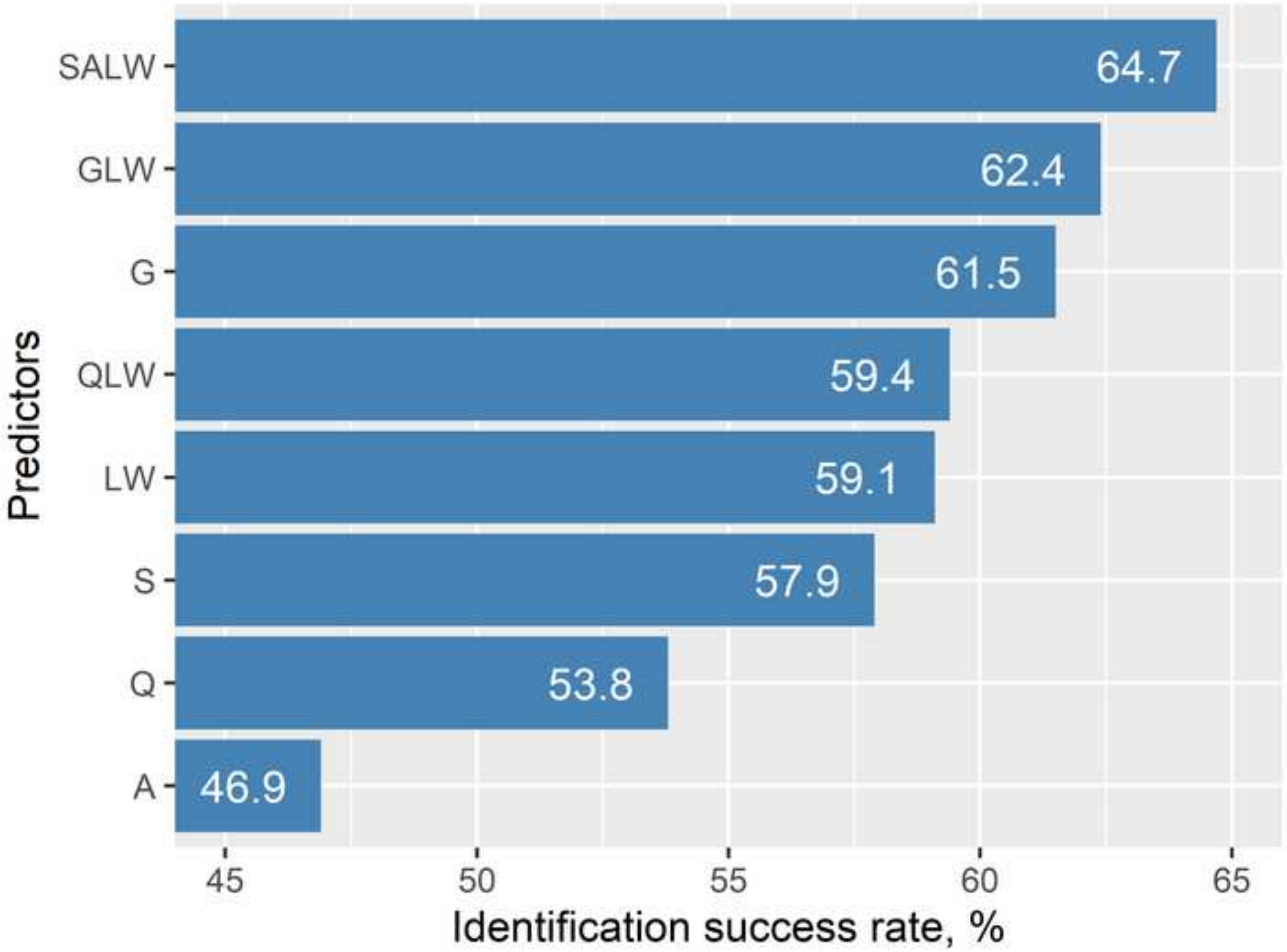

S1 Table

Click here to access/download
Supporting Information
S1 Table.tsv

Click here to access/download
Supporting Information - Compressed/ZIP File Archive
S2 Appendix.zip
