# Supplementary material for: Geometric morphometric analysis of spore shapes improves identification of fungi
Source: PLoS One. 2021 Aug 5;16(8):e0250477. doi: 10.1371/journal.pone.0250477 (PMC8341628; doi:10.1371/journal.pone.0250477)
Supplement: Attachment — Submitted filename: Response to Reviewers.docx [file pone.0250477.s004.docx]

PONE-D-21-10269
Quantitative analysis of spore shapes improves identification of fungi
PLOS ONE

**Review Comments and Responses**

Reviewer‘s comments are provided in italics, and our responses in regular font. If available, the number in front of the reviewer’s comment refers to the line numbers in the original (not revised) manuscript.

### Reviewer 1

*The manuscript "Quantitative analysis of spore shapes improves identification of fungi" is technically sound and all data support the conclusions. Applied methodology is very innovative in the field of mycology, especially because it is used for the purposes of distinguishing different fungal species more easily and correctly. In my opinion statistical analysis is performed appropriately. Authors ensured that all data from the manuscript are available to the potential readers. English is quite good but I would suggest additional final proofing by a native English speaker if possible. As well, I would suggest that you consider my comments and apply necessary changes to improve the manuscript. My highest concern is that you didn't find many mycological articles dealing with spore shapes even though there are many of them. I proposed some of them for you to read and cite if you find them appropriate (I think they are).*

We thank Reviewer 1 for the evaluation of the manuscript. It helped to broaden our somewhat “biased” Basidiomycota-centric view on the fungal spore morphology. We revised the manuscript with copyediting help from a colleague experienced in scientific English writing. Additionally, we used a commercial digital writing assistant software pre-set for scientific English writing.

We re-wrote the Introduction and Discussion to make clear we meant few studies that treated spore shapes as purely numeric information that was captured by digitizing the outline coordinates. We thank for pointing to important literature sources that we brought into the manuscript, although partly in a different way than proposed by reviewer.

*Please sort keywords alphabetically*

In the example given in instructions for authors of PLOS ONE, the keywords are not listed alphabetically. We also preferred to sort them by priority (the earlier mentioned = more important). We also added “size” to the keywords. The final set of keywords is:

“geometric morphometrics; Fungi; contour; outline; size; classification; traditional morphometrics”

PLOS also informs that keywords are not a part of a paper and are used only during the editorial process. Therefore we keep them only in the metadata and not the manuscript itself.

*[comments for the lines 63 and 65 are related and will be addressed below together]*

*63. Consult the article of Kušan et al. (2014) Biogeographical variability and re-description of an imperfectly known species Hamatocanthoscypha rotundispora (Helotiales, Hyaloscyphaceae). Phytotaxa https://doi.org/10.11646/phytotaxa.170.1.1*

*It should be cited here.*

*As well this is important article: Kauserud et al. (2008) https://doi.org/10.1016/j.funeco.2007.12.001*

*and this one:*

*Corner E. 1947 – Variation in the size and shape of spores, basidia and cystidia in Basidiomycetes. New Phytologist 46(2), 195–228 etc.*

*+*

*65. The work of Huhtinen (1990) A monograph of Hyaloscypha and allied genera. Karstenia*

*https://doi.org/10.29203/ka.1989.274*

*also studied differences among species and within population of the same species.*

*There is also: Matočec, N. (1998) The genus Scutellinia (Pezizales, Ascomycotina) in Croatia. II.: Scutellinia barlae and S. minor. Natura Croatica 7(2): 91-105.*

*and many others. Please repeat your search within mycological literature, you will find many examples*

*similar to those mentioned.*

Reviewer’s criticism is valid when applied to our original statement “To our knowledge, only two studies analyzed the shape of fungal spores quantitatively”. Therefore, we re-formulated the statement as “Unfortunately, there were few geometric studies of fungal spores and no quantitative comparison between shape and size descriptors (Fodor et al. 2015, Infantino et al. 2016)” (lines 83-84).

The studies of Kušan et al. (2014), Matočec (1998) and Huhtinen (1989) are high-quality taxonomic treatments. Like ours, these studies appeal to a more standardized treatment of spore shape information. Still, these studies deal with classical morphometrics because neither complete outline nor landmarks were captured from the spores. Nevertheless, these studies are relevant to ours in the context of reproducible taxonomy. They are cited when discussing future challenges of spore morphometric analyses (lines 391-394):

“For future studies, we recommend working with exclusively mature spores from spore prints as implemented in (Matočec 1998) and ensure an equal sample size as in (Corner 1947, Parmasto et al. 1987). Additionally, possible measurements biases from measuring fresh vs. dried spores and spores in different mounting media should be kept in mind (Matočec 1998, Kušan et al. 2014)”.

Kauserud et al. (2008), like the authors mentioned above, used length to width ratio as a proxy of spore shape. However, together with another study (Calhim et al. 2018), it is now cited in the Discussion as a groundstone for future ecological works (lines 413-417):

„Few attempts were made to link dispersal and deposition properties and a nutritional mode of fungi with the shape of their spores. However, the shape was expressed as a categorical variable (Calhim et al. 2018) or as a length to width ratio that provides limited geometric information (Kauserud et al. 2008). Therefore, we believe that including quantitative shape descriptors from the geometric morphometric analysis will provide a deeper understanding of the relationship between morphological and ecological traits in fungi“.

*105. searched*

The word was corrected.

*134. You should mention in which herbaria are your collections housed / from which herbaria you borrowed collections for your analysis*

Yes, we had to. We listed the herbaria acronyms, although earlier in the Methods where we believe it fits better. On lines 108-109, there is now a new sentence:

“The studied specimens belong to the herbaria ARAN, CWU, GB, LY, O, and KAS (abbreviations according to [27])”.

*137. Is here one number missing at the end of software version?*

Yes, the end of the version name was missing and therefore added. It was ImageMagick version 7.0.10-Q16.

*146. What was the number of the spores in the analysis? This information is very important.*

We agree that this info is very important. Already in the first submission, we provided all details at the beginning of Results. In the current revised version, these are lines 259-262:

“After all quality filtering steps, between 10 and 37 spores per specimen were available, totaling 511 spores from 30 specimens. These spores were captured in 401 images, and each image contained one to four spores (supporting information S2). Therefore we considered 401 average-per-image values as observations for the analyses”.

*208. In mycology this ratio (length/width) is called Q*

We are well aware that the symbol Q is often used to refer to length/width ratio in mycology. However, it can be named also in another way, like E in the work of Corner (1947) that reviewer recommends to consider. In several papers on protists that we have checked, no specific symbol was used (just words) or “L:W”. Irrespective of the symbol for referring to a trait, we feel that at this line of the manuscript, this technical detail is not relevant. It may not interest a broad audience of PLOS ONE journal. On the other hand, we clarify this symbol (Q), as well as all others, as soon as it becomes necessary – in the legend for Figure 6, lines 308-311.

*226. Citation missing!*

We now cite the work of Spearman (1904).

275. All of them ?

We changed the sentence to clarify that it applies to all three variables mentioned in the previous sentence. The new version is (lines 281-283):

“These three variables correlated strongly negatively with the length and moderately positively with the width of the spores”.

*307-308. This is not completely true, see the reviewer's comments in the Introduction chapter*

We agree with the reviewer and re-formulate the statement to make it valid as (lines 323-324):

“Concerning fungal spores, whose morphology is highly relevant for fungal taxonomy, geometric morphometrics was rarely applied so far”.

*373. Large number of fungal taxa, specially from the phyllum Ascomycota have radially symmetric spores where we cannot distinguish lateral and frontal view*

We thank reviewer for bringing forward this idea. We include it in lines 401-404: “On the other hand, there are numerous fungal taxa (e.g., from the phylum Ascomycota) with radially symmetric spores that are formed inside sporangia and lack any attachment loci. In this case, selecting the spores for outline extraction and processing digitized outlines will be more straightforward”.

*375. In Basidiomycota (not all fungi!)*

We removed this statement. In general, in the revised Discussion, we tried to talk on microscopic biological objects in general and avoid specific taxonomic cases as much as possible to keep the text easily readable by PLOS readers with a different scientific background.

*384. Unclear*

Corrected to “geometric morphometrics” (now line 409). We defined this term earlier on lines 39-41 and another term “landmark” on lines 43-44.

*Fig 1. Regarding the basidium which is the same as the one on the left side of the picture, this spore (frontal face, meaning adaxial side toward the viever) should be placed on the second sterigma (not on the first one) because of the 3D position of basidial sterigmata.*

We have changed Fig. 1 according to reviewer’s comment. We re-located basidiospore in frontal projection to another sterigma to account for anatomical correctness.

### Reviewer 2

*The authors should be commended for using NEFD to study spore morphology - it is an up and coming method for which there are few studies focused on fungi. The authors' procedural and statistical approaches are thorough and appropriate.*

We thank reviewer for the evaluation of the manuscript and a series of comments and suggestions. Below we describe how we addressed each of them.

*The manuscript would benefit from a greater narrative. The methods are very strong but they get lost without greater intellectual context. It was often unclear why this study was conducted, why the particular genus was selected, and what is the broader scientific question.*

We have substantially revised the Introduction to provide a better narrative. We have provided a clearer logical thread towards the large potential of geometric morphometrics for increasing efficacy of work with morphological traits and enhancing reproducibility in taxonomic research. We end Introduction by explaining selecting *Subulicystidium* as a study system, for which comprehensive (in terms of sample coverage) genetic data are available, along with the large set of images of sufficient quality for spore outline extraction. We explain that due to diverse spore shapes within *Subulicystidium,* they serve as representatives of spores in other fungal lineages and microscopic structures of other organisms.

*I found the hypotheses to be overly presumptuous - one employs NEFD precisely to answer the sorts of questions posed by the three hypothesis the authors give; so asking if NEFD works for that purpose, when it obviously does (otherwise the authors wouldn't have chosen to use NEFD) seems circular.*

Reviewer’s comment indicates that we separated the hypothesis of the study and the objectives not clearly enough. We also agree that the objectives about the appropriateness of NEFD were overly presumptuous. In the revised manuscript, we omit the objective and re-formulate hypothesis in a broader context of the usefulness of outline geometric information for species prediction. The paragraph with motivation and hypothesis (lines 62-69) now looks like:

„In the study of somewhat larger objects than fungal spores (genitalia in Lepidoptera), morphometric analyses resulted in more accurate taxon identification than subjective visual judgments (Mutanen and Pretorius 2007). Furthermore, the most accurate identifications were achieved for a combination of landmark-based geometric information and linear measurements. This is likely due to complementing two mathematically independent sources of evidence (Mutanen and Pretorius 2007, Zelditch et al. 2012). A digitized outline provides even richer geometric information than the landmark configuration. This study hypothesizes that combining linear measurements with geometric information on the outline results in better species identification than either evidence alone“.

*I was especially confused by Figure 6 - I had trouble understanding what that figure represents and how it was calculated. If the authors mean to show that NEFD can be used to predict species identity from spore shape I would be very surprised by that claim given that almost all of the shape variation could be explained by the first PC for symmetrical and asymmetrical analyses (which seemed to correspond to features that are not unique to Subulicystidium and I am doubtful that this method would work beyond the idiosyncrasies of the dataset the authors employed).*

We thank reviewer for this comment that made us to re-think and substantially improve the Discussion. Fig 6 summarises the results of discriminant analysis, where models with different compositions of predictors (some of “size” class and some of “shape”) are compared in terms of prediction of species identity. *Reviewer’s interpretation of the key message “NEFD can be used to predict species identity from spore shape“* agrees with ours only partly. We see the key message rather as “numeric shape descriptors improve species prediction”. With linear measurements only, species are predicted correctly in 59.1% of cases. When the shape descriptors are added to the model, species are predicted correctly in 64.7% of cases. Although little, this is an improvement that we are willing to communicate to the public. It is our understanding of how the science works, i.e., proposing a hypothesis for subsequent verification on different cases.

We agree that the first and strongest PCs of symmetric or asymmetric shape variation describe features that are not unique for this taxon (*Subulicystidium*). However, this is a well-known feature of geometric morphometric analyses, which can be attributed to the strong biological relationship between the shape and linear size of the organisms (Zelditch et al. 2012). Nevertheless, the way the discriminant model was built shows that when linear measurements from the object are supplemented by shape descriptors, the identification success rate increases.

We address reviewer’s comment with the following paragraph in Discussion, lines 350-367:

“While linear measurements predicted species correctly in 59.1% cases (LW model), adding shape descriptors increased the prediction rate to 64.7% (SALW model). The increase of nearly 5% may first appear as not prominent. We explain this pattern by including in the analyses the species with very different spores (e.g., from needle-shaped to short-cylindric). It made the weight of the first PCs (of symmetric and asymmetric variation) very high and caused them to correlate strongly with the linear measurements. A strong correlation of the first PC of shape variation with the linear size is a known phenomenon in geometric morphometrics (Zelditch et al. 2012). In our case, it could have contributed to overfitting the discriminant model and result in only a slight increase of success of identification in the SALW model compared to LW model. If the range of spore shape variation between the taxa were narrower, we expect the first PC to correlate weaker with the linear measurements and result in higher identification success rates in the SALW model. In the simulation with removing the 1^st^ PC of global variation from PCA scores for NEFD data, we found the subsequent PCs a bit more equalized in the amount of reflected variation (see supplementary information S2). In general, the success of automated identification depends on the range of variation in the data (Wishkerman and Hamilton 2018). Therefore, we argue it is essential not to focus on a bold number but rather to be able to disentangle the contribution of separate descriptors to the discriminant model. We are looking forward to seeing the results of discriminant analyses when applying our approach to other microscopic objects with different ranges of morphological variation”.

Reviewer can access the figure from the simulation with excluding the first PC directly on GitHub repository with the link <https://github.com/ordynets/size_vs_shape/blob/main/3_results/fig_suppl2_1pc_no.tiff>

As for other figures, in Fig 6, we upgraded the title by making it descriptive. The new title is: “Discriminant models including both size and shape descriptors outperform those with either predictor type”. We also upgraded the legend by adding an additional sentence in the beginning: “For each value on y axis, species identification success rate from discriminant analysis is displayed as an average value from 1000 replicates of cross-validation”.

*I thought the figures need a lot of work and greater explanation in the legends.*

We checked our figures against the PACE tool and ensured that they technically meet PLOS requirements.

In addition to upgrading the title and legend in Fig 6, we went through these elements in all other figures. We changed the titles to descriptive ones. On the other hand, we kept them concise and <15 words long according to PLOS instructions. To better understand the figures, we added the legend to Fig 1 and extended legends for Figs 4-6. On the other hand, when writing or adjusting the legends, we kept in mind the recommendation of PLOS to “avoid lengthy descriptions of methods“. We believe we provided enough details in Methods to understand the figures’ content.

*I would have liked to have seen more explanation on the imaging protocols besides a citation to past work…*

We would like to say that we also do not like the approach of shortening the Methods by simply referring to an earlier publication. However, in our case, we cannot name Ordynets et al. 2021 (on protocols.io) just a “past work”. It is a citable protocol specifically developed for this research paper. It is also a part of current PLOS policy – to publish methods as a standalone outcome and refer to them in the Methods part of the paper (see paragraph “Protocols associated with Research Articles” at <https://journals.plos.org/plosone/s/submission-guidelines#loc-parts-of-a-submission>).

We went once more through Methods part describing image acquisition and pre-processing (lines 130-159), shape data extraction and processing (lines 161-200), and size data extraction and processing (lines 202-211). We ensured that all steps are sufficiently explained in the manuscript, in the form of text and also graphical summary in Fig 2.

*…as well as an explanation as to why different numbers of images were used per species and how sample size differences were handled.*

We agree sample size difference is an important issue that should be more thoroughly discussed. Therefore, we added the following paragraph to the Discussion, lines 376-394:

“In another morphometric analysis, i.e., of statolith shapes in Cubomedusae, the identification success rate was highly dependent on the number of observations per group [33]. Due to technical reasons explained in the Methods section, we performed analyses on the average-per-image data. We admit that working with spore-level data would provide a more precise estimate of the correct identification rate. To include ten species and three specimens for each species into analyses, we acquired images of spores from squash preparations of dried herbarium specimens collected between 1978 and 2016. Such spore samples may include unmature spores that can affect measurements results [66]. We believe we could essentially minimize this risk due to our previous fungal taxonomy experience and merely by considering floating (not attached to basidia) basidiospores. Squash preparations from herbarium specimens do not always provide the same number of well-preserved spores. We were able to measure 10 and 37 spores per specimen while it was recommended to measure at least 10 spores but better 30 for the study of spore size [8]. We counteracted the sample size difference by the design of discriminant analysis. There, we took a subset of 70% of measurements for each species for building the training data. Furthermore, in each of 1000 iterations of cross-validation, different subsamples of spores per species were drawn for building the training and test data. For future studies, we recommend working with exclusively mature spores from spore prints as implemented in [66] and ensure an equal sample size as in [8,67]. Additionally, possible measurements biases from measuring fresh vs. dried spores and spores in different mounting media should be kept in mind [10,66,68]”.

*Linguistic usage use also requires a fair amount of work, especially in the introduction and conclusion.*

We revised the manuscript with copyediting help from a colleague experienced in scientific English writing. Additionally, we used a commercial digital writing assistant software pre-set for scientific English writing.
